# Quantifying Correlogram Shape to Analyze Neuronal Firing Dynamics Recorded in TBI-on-a-Chip

**DOI:** 10.1007/s12021-026-09770-9

**Published:** 2026-04-27

**Authors:** Casey Erin Adam, Shatha J. Mufti, Jhon Martinez, Edmond A. Rogers, Martina Dalolio, Nikita Krishnan, Timothy Beauclair, Riyi Shi

**Affiliations:** 1 Center for Paralysis Research, Purdue University, West Lafayette, IN 47907 USA; 2 Department of Basic Medical Sciences, School of Veterinary Medicine, Purdue University, West Lafayette, IN 47907 USA; 3 Weldon School of Biomedical Engineering, Purdue University, West Lafayette, IN 47907 USA; 4 Indiana University School of Medicine, 340 West 10 StreetSuite 6200, Fairbanks HallIndianapolis, IN 46202-3082 USA

**Keywords:** Raster, Correlogram, Bicuculline, Alkalosis, Injury, Software

## Abstract

Changes in neuronal network dynamics in response to different treatments or conditions underly brain function and pathology. A multitude of tools, including electroencephalography, voltage or calcium imaging, and microelectrode array (MEA) recordings, exist to record signals from neuronal firing at different length scales. Correlograms are a standard analysis tool to study the relationship between a pair of recorded neuronal signals. Correlogram shape can provide information about signal independence, firing pattern, and firing order. However, such analysis is performed manually and qualitatively, limiting the amount of information gained. To overcome this limitation, a MATLAB algorithm was developed to automate correlogram shape quantification by calculating correlogram uniformity, peak count and location, and area left of zero, which respectively quantify signal independence/dependence, firing pattern, and firing order. Algorithm outputs were validated using three different MEA recordings during which cells were exposed to bicuculline methiodide, pH shock, or impact injury. Algorithm outputs described signaling changes in all three recordings, bridged changes in individual signal pairings to changes in the entire signal population, and agreed with literature studies. Therefore, this algorithm serves as a useful means of automatically quantifying changes in signal dependence, firing pattern, and firing order across time within a single recording, and across different recordings. These features are also common to all recording techniques, and therefore can be compared across different types of recordings. Therefore, the MATLAB algorithm described in this article can help provide insight into how neural network dynamics are altered by different drugs or conditions.

**Supplementary Information:**

The online version contains supplementary material available at 10.1007/s12021-026-09770-9.

## Highlights

Quantifying correlogram uniformity (signal independence/dependence), peak count and location (firing pattern diversity), and area left of zero (firing order) provides information on network dynamics.

Compared to standard approaches that estimate functional connectivity from correlograms, the above quantification comprises an alternative approach that…

Quantifies network dynamics without risk of mistaking correlations as cause

Uses metrics easier to compare across different length scales or recording types

However, provides less connectivity insight without additional quantification or measurements.

A MATLAB algorithm that automates this alternative approach is provided and described.

The algorithm is validated with three different microelectrode array recordings during which cells were exposed to bicuculline methiodide, pH shock, or impact injury.

Algorithm outputs bridge specific signals to the population of signals and do not lose or conceal information on heterogenous network responses.

**Supplementary Information:**

The online version contains supplementary material available at 10.1007/s12021-026-09770-9.

## Introduction

Neuron signaling is essential for maintaining organism health, sensory processing, muscle contraction, circadian rhythms, and cognition (Akay, 2023). Conditions such as injury alter how neurons relay signals, which can lead to pathological brain rewiring (Ahmed et al., 2017; Akay, 2023; Bramlett & Dietrich, 2015; Frankowski et al., 2022; Shah et al., 2022). For example, traumatic brain injury (TBI) can enhance neuronal excitability, change neuronal connectivity, and increase the long-term risk of developing neurodegeneration, depression, psychological conditions, and epilepsy (Ahmed et al., 2017; Bramlett & Dietrich, 2015; Shah et al., 2022). Therefore, recording and quantifying how signaling between neurons changes in a variety of conditions or treatments could help clarify how various pathologies evolve and thereby improve understanding, treatment, and early detection of neurological conditions.

Growing interest in mechanistic studies of neuronal firing has not only advanced the capabilities of whole-brain recordings in living individuals, but also prompted development of in vitro platforms (Alaylioglu et al., 2020; Belle et al., 2018; Kolev, 2011; Ren et al., 2024; Vertes & Stackman, 2011; Wickenden, 2000). While in vitro platforms do not always predict in vivo effects, fundamental responses are often similar between the two platforms (Alaylioglu et al., 2020; Belle et al., 2018). Therefore, in vitro platforms are employed in simplified mechanistic studies to facilitate cell access and eliminate confounding variables arising from tissue architecture and other physiological processes (Alaylioglu et al., 2020; Belle et al., 2018). For example, TBI-on-a-Chip, in which primary neuronal networks are cultured on microelectrodes, is an in vitro platform to study how TBI alters cell function and causes different brain pathologies (Beauclair et al., 2024; Rogers & Gross, 2019; Rogers et al., 2022, 2023, 2025). TBI-on-a-Chip has already been employed to understand how injury links to morphological changes in cells, chronic inflammation, oxidative stress, and neurodegeneration (Beauclair et al., 2024; Rogers & Gross, 2019; Rogers et al., 2022, 2023, 2025). In TBI-on-a-Chip, different chemicals or drugs, as well as in vitro injuries, including blast pressure waves (Beauclair et al., 2024) or impact injuries (Rogers & Gross, 2019; Rogers et al., 2022, 2023, 2025), can be applied to the cultures to simulate brain injury. Different pathologies introduced by injury can then be readily investigated by measuring changes in cell firing, protein expression, and cell morphology with greater access than that available in an in vivo brain (Beauclair et al., 2024; Rogers & Gross, 2019; Rogers et al., 2022, 2023, 2025). However, to effectively compare in vivo and in vitro results, data analysis frameworks capable of adapting to different recording techniques, length scales, and experimental settings are required.

Firing in neuronal populations can be experimentally measured via a variety of techniques that record voltage or fluorescence through time for each unique signal. Techniques that record voltage through time include microelectrode array (MEA, used both in vitro and in vivo) (Obien et al., 2015) or electroencephalogram (EEG, used in vivo) (Michel & Brunet, 2019) recordings. In both techniques, each signal corresponds to a particular electrode or unit (Michel & Brunet, 2019; Obien et al., 2015). Note that, for MEA recordings only, units can also be signals with discriminable voltage waveforms detected by the same electrode (Obien et al., 2015). In MEA recordings, each signal corresponds to a single cell or a small group of cells, depending on electrode size (Obien et al., 2015). In EEGs, each signal corresponds to a summation of postsynaptic potentials from populations of neurons within a particular brain locality (Michel & Brunet, 2019). Similarly, techniques such as calcium (Grienberger & Konnerth, 2012) or membrane voltage (Peterka et al., 2011) imaging record fluorescence through time, can be used both in vitro and in vivo, and each signal corresponds to a particular cell or a small group of cells. Measurements obtained using different techniques are related (Fedele et al., 2020), but linking recordings at different length scales is difficult because the nature of the recorded signals differs.

Regardless of the recording technique used and whether firing is measured at a group (EEG and some MEA) (Michel & Brunet, 2019; Obien et al., 2015) or individual (imaging and some MEA) (Grienberger & Konnerth, 2012; Obien et al., 2015; Peterka et al., 2011) level, recording data are processed in a similar manner. Neuronal firing events are extracted from the raw signal by identifying where a particular signal shape (for example, an epileptiform or k-complexes in EEGs) or sharp changes in the measured voltage signal, called a spike, occur (Bénar et al., 2007; Cheveigné & Arzounian, 2015; Iannello et al., 2024). The times at which each firing event occurred for each signal are saved as a list called a raster (Bénar et al., 2007; Cheveigné & Arzounian, 2015; Iannello et al., 2024). If *N* signals were recorded, *N* rasters exist for the recording. Rasters are then used in many ways to perform subsequent data analysis (Bénar et al., 2007; Cheveigné & Arzounian, 2015; Iannello et al., 2024).

One established method of analyzing rasters, employed since the 1960s, is crosscorrelation analysis (Awiszus, 1992; Durstewitz, 2017; Eggermont, 2014; Gerstein & Perkel, 1972; Iannello et al., 2024; Kobayashi et al., 2019; Melssen & Epping, 1987; Moore et al., 1966, 1970; Narayanan & Laubach, 2009; Pereda et al., 2005; Perkel et al., 1967; Saberi-Moghadam et al., 2018; Shao & Tsau, 1996; Viejo et al., 2023). In crosscorrelation analysis, a conditional probability distribution called a correlogram is created to describe the probability a comparison signal occurs given that a reference signal occurred at a specific time (Nex Perkel et al., 1967; Technologies, 2024). To generate correlograms, the raster of a single signal is compared against a reference raster (Awiszus, 1992; Eggermont, 2014; Gerstein & Perkel, 1972; Iannello et al., 2024; Kobayashi et al., 2019; Melssen & Epping, 1987; Moore et al., 1966, 1970; Narayanan & Laubach, 2009; Nex Technologies, 2024; Perkel et al., 1967; Saberi-Moghadam et al., 2018; Shao & Tsau, 1996). A correlogram is referred to as an autocorrelogram if the reference and comparison signal are the same, a crosscorrelogram if the reference and comparison signals are different, or a perievent histogram (also called a peristimulus or poststimulus time histogram) if the reference raster indicates when a particular event, such as drug treatment, occurred (Moore et al., 1966; Nex Technologies, 2024). Autocorrelograms, crosscorrelograms, and perievent histograms respectively address how a signal varies with respect to itself, to another signal, or to an event such as a stimulus (Moore et al., 1966; Nex Technologies, 2024). Regardless of type, correlograms are histograms of relative signal timings (Moore et al., 1966; Nex Technologies, 2024; Perkel et al., 1967). When normalized, the correlogram distribution represents the probability of a comparison event (such as a spike/firing) at time $t$ given a reference event occurred at $t=0$ (Nex Technologies, 2024). The shape of a correlogram indicates features about the relationship between the comparison and reference rasters (Eggermont, 2014; Gerstein & Perkel, 1972; Moore et al., 1966, 1970; Perkel et al., 1967). In particular, uniform (flat) correlograms indicate that the reference and comparison rasters fire independently of one another, correlogram peak count relates to firing pattern, and distribution of the correlogram relative to $t=0$ reflects firing order (Eggermont, 2014; Gerstein & Perkel, 1972; Moore et al., 1966, 1970; Perkel et al., 1967; Rogers & Gross, 2019).

In many cases, crosscorrelograms are employed to understand dynamics throughout a recording of neuronal firing (Awiszus, 1992; Durstewitz, 2017; Eggermont, 2014; Gerstein & Perkel, 1972; Iannello et al., 2024; Kobayashi et al., 2019; Melssen & Epping, 1987; Moore et al., 1966, 1970; Narayanan & Laubach, 2009; Pereda et al., 2005; Perkel et al., 1967; Saberi-Moghadam et al., 2018; Shao & Tsau, 1996; Viejo et al., 2023). Most of the previous work in quantifying correlogram properties has focused on using correlograms to infer neuronal connectivity (Awiszus, 1992; Bartram et al., 2024; Durstewitz, 2017; Eggermont, 2014; Gerstein & Perkel, 1972; Hu et al., 2022; Ito et al., 2014; Kobayashi et al., 2019; Melssen & Epping, 1987; Moore et al., 1966, 1970; Narayanan & Laubach, 2009; Pereda et al., 2005; Perkel et al., 1967; Saberi-Moghadam et al., 2018; Shao & Tsau, 1996; Wang et al., 2024) and underlying postsynaptic currents (Awiszus, 1992). While such approaches can identify likely neuronal connections, it is important to consider that functional connectivity inferred from correlograms is ultimately based on correlations in relative signal timings and may therefore not be causal (Awiszus, 1992; Durstewitz, 2017; Eggermont, 2014; Gerstein & Perkel, 1972; Kobayashi et al., 2019; Melssen & Epping, 1987; Moore et al., 1966, 1970; Narayanan & Laubach, 2009; Pereda et al., 2005; Perkel et al., 1967; Saberi-Moghadam et al., 2018; Shao & Tsau, 1996). Furthermore, because the nature of functional connections differs between recording techniques (Fedele et al., 2020; Grienberger & Konnerth, 2012; Michel & Brunet, 2019; Obien et al., 2015; Peterka et al., 2011), functional connectivity inferred from correlograms is difficult, but not impossible, to compare across different experimental techniques and settings. However, correlograms can also be used to obtain other useful information about neuronal firing dynamics without relying on correlations in signal timing. For example, for cultures that exhibit rhythmic and synchronous firing, a hallmark of mature neuronal networks (Beggs & Plenz, 2003, 2004), correlogram peak times have been used to quantify the frequency of the rhythmic firing activity (Ito et al., 2014; Rogers & Gross, 2019).

While correlogram metrics such as uniformity, peak count, and distribution relative to $t$ = 0 do not assume that the firing of one signal causes another, relatively few studies have used these metrics to assess changes in signal firing. For example, uniformity, peak count, and distribution relative to $t=0$ are often qualitatively summarized or mentioned in passing before a different metric is quantified (Bartram et al., 2024; Ito et al., 2014; Nex Technologies, 2024; Rogers & Gross, 2019). Additionally, some software platforms plot, but do not quantify correlograms so users can tailor correlogram quantification to the specific application (Nex Technologies, 2024; Viejo et al., 2023). Other studies qualitatively compare auto- and cross-correlogram shapes to ensure that signal sorting is not detecting the same signal from different electrodes (Rathbun et al., 2024). Most studies do not mention these three metrics, or use correlogram peaks to infer functional connectivity (Bartram et al., 2024; Eggermont, 2014; Hu et al., 2022; Ito et al., 2014; Kobayashi et al., 2019; Melssen & Epping, 1987; Narayanan & Laubach, 2009; Saberi-Moghadam et al., 2018; Shao & Tsau, 1996; Wang et al., 2024). Therefore, tools to quantify correlogram uniformity, peak count, and distribution relative to zero are limited. However, quantifying these metrics together would avoid correlational analysis while still identifying altered firing dynamics from correlograms with altered shape metrics in response to experimental treatments.

Typically, to perform crosscorrelation analysis in commercial software, the user manually selects several small regions of the recording (for example, four regions, each 200 s in duration (Rogers & Gross, 2019)) from which to generate and analyze correlograms (Nex Technologies, 2024). After correlograms are generated, the user must manually visualize each correlogram by eye in each analyzed region of the data. If a recording has $N$ signals, then there are ${N}^{2}$ correlograms (cross- and autocorrelograms) to examine for each region of the recording, meaning that the user often ends up manually analyzing thousands of correlograms or selecting only a subset of correlograms to consider. Correlogram uniformity, peak count, and position relative to $t$ = 0 are typically not quantified because manually quantifying such properties for thousands of correlograms in many different recording regions is too time consuming. While a multitude of alternative custom software platforms exist to view and quantify correlogram properties, these platforms also do not quantify correlogram uniformity, peak count, and distribution relative to $t=0$ (Hu et al., 2022; Ito et al., 2014; Narayanan & Laubach, 2009; Viejo et al., 2023; Wang et al., 2024). It is therefore difficult to compare correlogram shape across a single recording, biological replicates, and different experimental treatments.

This article describes the creation and validation of a custom MATLAB script designed to generate correlograms, quantify correlogram shape properties (uniformity, peak count, and distribution left of zero), and display the resulting distributions and classifications of these properties, thereby eliminating the need to perform such quantifications manually. This script is available on the GitHub page linked with this publication and in the supplementary material. The script loads a recording file, calculates basic recording metrics, separates the recording into different regions, generates correlograms in each region, then quantifies correlogram uniformity, peak count and location, and distribution relative to $t$ = 0 for each correlogram. Distributions of these metrics are then plotted to allow comparison across recording regions. The algorithm was validated on MEA recordings from TBI-on-a-Chip to ensure that these three metrics can describe changes in cell interactions and provide useful information on neuronal firing in a variety of conditions. Specifically, the script was employed to analyze neuronal firing dynamics in recordings with varying durations, unit count, and experimental treatments. The three treatments tested were: bicuculline methiodide, pH shock, and impact injury. Correlogram shape metrics generated by the algorithm exhibited the expected changes due to each treatment. Therefore, the algorithm serves as an effective means of using correlogram shape quantification, without assuming functional connectivity, to enhance understanding of how different drugs or conditions affect the firing dynamics of neuronal populations.

## Methods

**Fig. 1:**
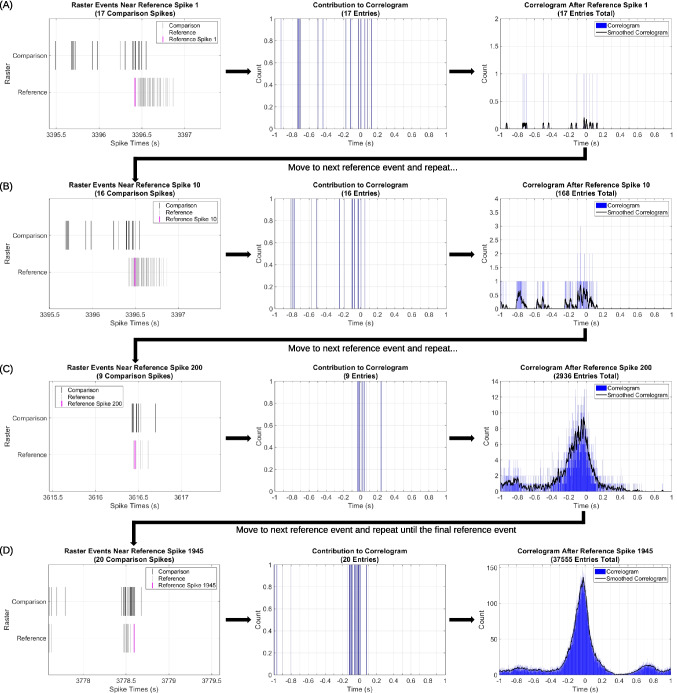
Correlogram creation schematic. Correlograms are histograms of relative event timings and correlogram creation is a well-established process (Awiszus, 1992; Durstewitz, 2017; Eggermont, 2014; Gerstein & Perkel, 1972; Iannello et al., 2024; Kobayashi et al., 2019; Melssen & Epping, 1987; Moore et al., 1966, 1970; Narayanan & Laubach, 2009; Nex Technologies, 2024; Pereda et al., 2005; Perkel et al., 1967; Saberi-Moghadam et al., 2018; Shao & Tsau, 1996). To generate a correlogram, one raster is set as a reference and the other compared against the reference. In this figure, each raster event (left column) corresponds to a spike, or firing event, from a single neuron in an MEA recording. First (**panel A**), comparison event times are shifted by subtracting the time of the first reference event (magenta bar in the left column). A histogram of shifted comparison event times within the correlogram range ($\pm$ 1 s in this figure) and bins (1 ms wide in this example) is then created (middle column). This contribution histogram is then added to the bin counts of the correlogram (right column). The entire process is repeated for each reference event. **Panel B** shows the same process for reference event 10, **panel C** for reference event 200, and **panel D** for the final reference event (1,945). Once created, the correlogram is normalized to probability. Therefore, the correlogram represents the probability of a comparison event at time $t$ given a reference event occurred at $t$ = 0 s

Correlogram creation is already well established (Awiszus, 1992; Eggermont, 2014; Gerstein & Perkel, 1972; Iannello et al., 2024; Kobayashi et al., 2019; Melssen & Epping, 1987; Moore et al., 1966, 1970; Narayanan & Laubach, 2009; Nex Technologies, 2024; Perkel et al., 1967; Saberi-Moghadam et al., 2018; Shao & Tsau, 1996). To generate a correlogram, each event in the reference raster, occurring at time ${t}_{r}$, is used to assign time zero ($t=0$) (Gerstein & Perkel, 1972; Nex Technologies, 2024; Perkel et al., 1967). Comparison events, occurring at time ${t}_{c}$, are then shifted relative to ${t}_{r}$ ($t= {t}_{c}- {t}_{r}$) (Gerstein & Perkel, 1972; Nex Technologies, 2024; Perkel et al., 1967). A comparison spike only contributes to a correlogram if $t$ falls within a specified range around 0 (Gerstein & Perkel, 1972; Nex Technologies, 2024; Perkel et al., 1967). For example, if the correlogram range is $\pm$ 200 ms, then a spike only contributes to the correlogram if $\left|t\right|\le 0.2$ s (Gerstein & Perkel, 1972; Nex Technologies, 2024; Perkel et al., 1967). All comparison spikes that fall within the range of a given ${t}_{r}$ are then assigned to time bins within the specified range. For example, if 1 ms wide bins are used, and ${t}_{c}$ occurs 2.5 ms after ${t}_{r}$, then the comparison spike ${t}_{c}$ would be assigned to time bin 2. Similarly, if ${t}_{c}$ occurs 6.5 ms before ${t}_{r}$, the spike is assigned to time bin −6. All ${t}_{c}$ within the specified range are assigned to time bins in this manner. This process is then repeated for each ${t}_{r}$, and the bin counts summed over all ${t}_{r}$ (Gerstein & Perkel, 1972; Nex Technologies, 2024; Perkel et al., 1967). This process is shown in Fig. 1. The correlogram can then be normalized to obtain probability by dividing the value in each bin by the sum of all bin counts. The center bin of the correlogram is always located at $t=0$ (Gerstein & Perkel, 1972; Nex Technologies, 2024; Perkel et al., 1967). The shape of a correlogram indicates features about the relationship between the comparison and reference rasters (Eggermont, 2014; Gerstein & Perkel, 1972; Moore et al., 1966, 1970; Perkel et al., 1967). In particular, a correlogram can provide insight into signal dependence, firing pattern, and firing order (Eggermont, 2014; Gerstein & Perkel, 1972; Moore et al., 1966, 1970; Perkel et al., 1967).

### Correlogram Shape Quantification

While multiple different types of software exist to create and analyze correlograms, it is often difficult to quantify correlogram shape properties automatically with the available tools (Awiszus, 1992; Bartram et al., 2024; Eggermont, 2014; Gerstein & Perkel, 1972; Hu et al., 2022; Ito et al., 2014; Kobayashi et al., 2019; Melssen & Epping, 1987; Moore et al., 1966, 1970; Narayanan & Laubach, 2009; Nex Technologies, 2024; Perkel et al., 1967; Saberi-Moghadam et al., 2018; Shao & Tsau, 1996; Viejo et al., 2023; Wang et al., 2024). To automate correlogram shape quantification and analysis, and thereby increase the amount of information that can be gained from a single recording, a custom script was written in MATLAB R2021b. This script is available on GitHub (https://github.com/ceadm/Correlogram-Uniformty-Peak-Count-Area-Left-of-Zero) and in the supplementary material of this publication. A summary of parameters controlled by the user to execute this script can be found in Table 1.

**Fig. 2:**
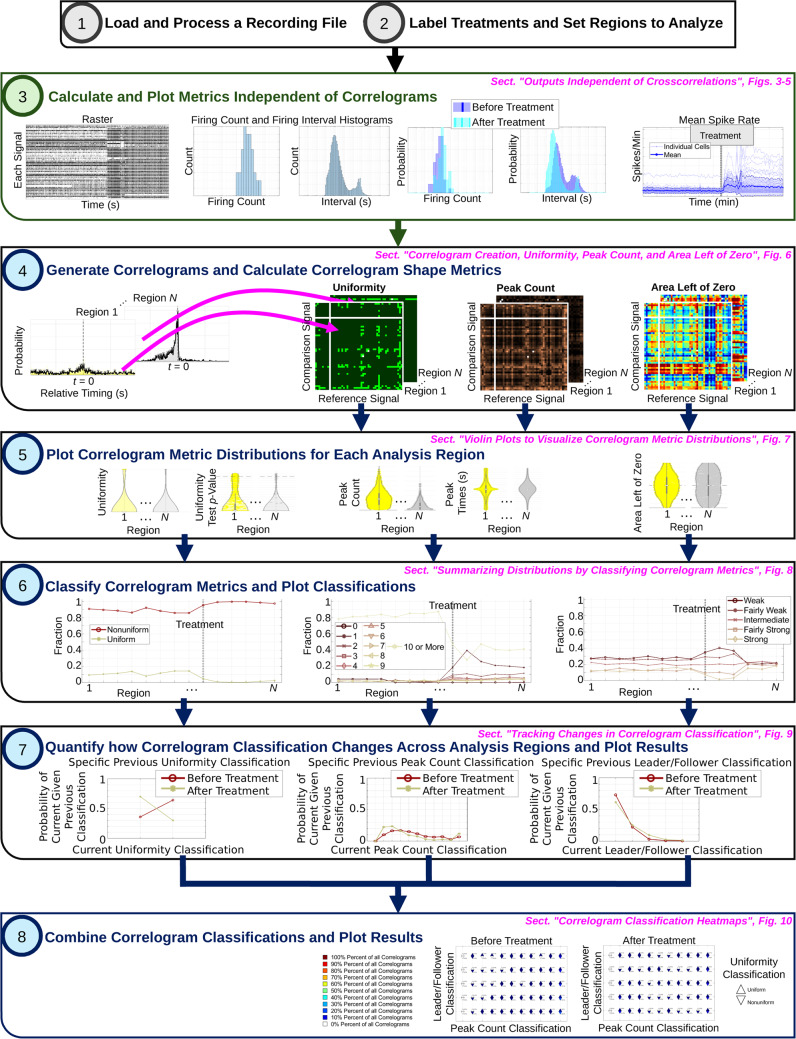
Algorithm flow chart. The algorithm described in this article consists of eight steps in which data from a single recording are loaded and processed (black/grey), then recording metrics independent (green) and dependent (blue) on correlograms are calculated and plotted. Example algorithm outputs are shown, with paper section and figure references in magenta

To generate correlograms, the script first divides the recording into user-specified regions called analysis regions. The user can manually select and label regions of interest, or allow the algorithm to automatically divide the recording into segments of a user-specified length. Regardless of how the user wishes to select regions, correlograms are generated from the rasters in each analysis region. In this article, recordings were automatically divided into seven minute segments in order to reduce the likelihood of sparse correlograms (longer regions mean more raster events in each region) while still obtaining fine time resolution through the recording. In general, analysis region duration should be selected based on the number of unique signals and how often signals fire in any given recording. Regions that are too long will contain too many raster events and thus require too much computational power to process. Regions that are too short will contain too few raster events, and correlograms will therefore be too sparse to accurately represent network dynamics. More detail on how computational complexity scales with the number of raster events can be found in Discussion Sect. "Computational Complexity". Note that plots generated by the algorithm (steps 5, 7, and 8 in Fig. 2) can either be separated by treatment, as in this article, or by analysis region, depending on user preference.

The user controls correlogram range and number of bins. If the user desires correlogram bins of a specific width, for example 1 ms, the user can calculate the number of correlogram bins by dividing the correlogram range by half the desired bin width. For example, if the correlogram ranges $\pm$ 7 s around $t$ = 0, the user can ensure 1 ms bins by setting the number of bins to *ceil(*7/.0005*)*. The *ceil()* command is to ensure that the number of bins is an integer. Correlograms with user-specified properties are then generated for all signal pairs in each analysis region. However, if the user desires, there is an option in the script to use only specific signals as reference rasters. This option is provided to facilitate perievent histogram analysis or target specific correlograms. For the recordings in this article, correlogram range was $\pm$ 1 s and each bin was 1 ms wide.

**Fig. 3:**
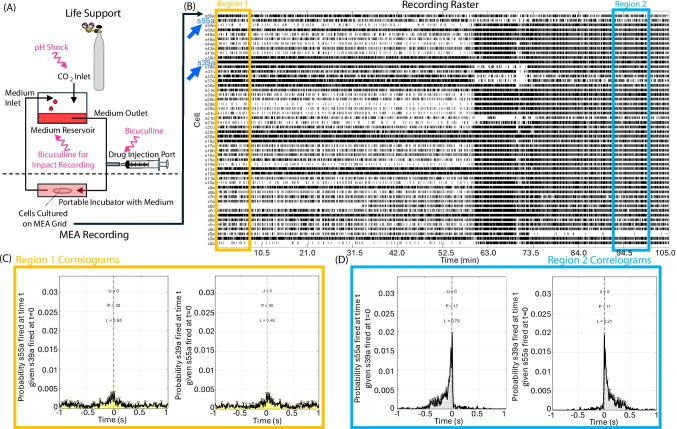
Experimental schematic and representative data. **Panel A** shows the experimental schematic for the microelectrode array (MEA) recordings used in this article. The culture was placed in a small incubator through which culture medium flowed via a peristaltic pump connected to a medium reservoir. Culture temperature was maintained at 37 °C via resistive heating. Culture pH was maintained constant via buffering provided by a CO_2_ line connected to the reservoir. Closing the CO_2_ inlet allowed pH shock to be delivered to a culture by removing buffering. Drugs could also be administered to the culture medium via a drug injection port attached to the medium flow. Treatments administered during separate recordings used in this article are indicated in pink (squiggly arrows) and include administration of bicuculline methiodide or pH shock. A third recording of a culture subjected to impact injury was also performed. Injury was administered by disconnecting the culture from the recording setup, administering tangential acceleration impacts, then reconnecting to the recording setup. The dashed line separates components of life support (above) and MEA recording (below). A representative raster from a single MEA recording is shown in **panel B**. Each row shows a unique signal, corresponding to a single cell in the culture, and black bars represent times at which the given cell fired. Correlograms are created from the rasters of two individual signals. The analysis algorithm described in this article generates and analyzes correlograms for each unique pair of signals. Correlograms of specific signal pairs can be plotted if desired by the user. Examples of such plots for correlograms created using the raster of signal s55a and s39a (dark blue arrows in panel B) at the start (**panel C**, region indicated by the yellow box in B) and end (**panel D**, region indicated by the light blue box in panel B) of a recording are shown. Correlogram uniformity (U), peak count (P), and area left of zero (L) are also displayed above each correlogram. The correlogram of s39a vs. s55a is the reflection of the correlogram of s55a vs. s39a about time = 0 s. The reflection has the same U and P, but complimentary L

If a correlogram is sparse, consisting of only a few raster events, metrics calculated from the sparse correlogram could be the result of sparsity artifacts, not real signaling relations. The user has the option to exclude sparse correlograms, if desired, by setting a threshold number of raster events below which the correlogram will not be analyzed. This threshold will vary based on the raster, correlogram range, and number of correlogram bins. To set a threshold for sparsity, the user should plot correlograms (an optional feature of the script, with outputs shown in Figs. 1D and 3C and D) and determine the sparsity threshold via visual inspection. If visual inspection reveals sparse bars, true peaks are difficult to distinguish, and overall distribution shape is unclear, the correlogram is too sparse. Examples of sparse correlograms are shown in Fig. 1A-C. The user can determine a cutoff number of raster events based on the lowest event count (displayed in the title of correlogram plots) where the correlogram distribution is easy to determine by eye. Visual inspection is the most foolproof means of setting the sparsity threshold without increasing computational complexity. Alternatively, the user can choose not to discount sparse correlograms, as in this article. The user may do so by setting the sparsity threshold to 0, which means correlograms with more than 0 raster events will be analyzed. If sparse correlograms are not discounted, it is essential to consider the number of events contributing to each correlogram when interpreting correlogram metrics.

After correlogram generation, the algorithm describes correlograms with three metrics: uniformity, peak count, and area left of zero. These metrics are automatically calculated for each correlogram, then plotted in various ways, shown in Figs. 6, 7, 8, 9, and 10. Details on how each metric is calculated are provided in Sects. "Quantifying and Classifying Signal Dependence"-"Quantifying and Classifying Firing Order".

#### Quantifying and Classifying Signal Dependence

If the comparison and reference rasters are independent of one another, *i.e.* comparison spikes occur randomly relative to reference spikes, the correlogram will be uniform (flat, without prominent peaks) (Gerstein & Perkel, 1972). If there is a relationship between the reference and comparison raster, knowledge of reference spike timings allows more accurate prediction of comparison spike timings, and the correlogram will be nonuniform (Gerstein & Perkel, 1972). Therefore, signal (in)dependence can be assessed by determining whether a correlogram is uniform (Gerstein & Perkel, 1972; Moore et al., 1966, 1970). If the correlogram is uniform, the signals are independent of each other (Gerstein & Perkel, 1972; Moore et al., 1966, 1970). If nonuniform, there is a temporal relation between the comparison and reference signals (Gerstein & Perkel, 1972; Moore et al., 1966, 1970).

It is important to note that nonuniform correlograms can be the result of many factors, including direct communication via synapses or synapse chains, synapse-free synchronization, shared input, or other correlated activity (Durstewitz, 2017; Gerstein & Perkel, 1972; Moore et al., 1966; Pereda et al., 2005). Nonuniformity does not guarantee synapsing between the cells contributing to each signal, nor does nonuniformity guarantee that one signal causes firing of the other signal (Durstewitz, 2017; Gerstein & Perkel, 1972; Moore et al., 1966, 1970; Pereda et al., 2005). For example, chemically induced synchronization or other forms of common input could cause neurons to fire at the same time, thereby showing dependence, even in the absence of interactions between the cells (Durstewitz, 2017; Gerstein & Perkel, 1972; Moore et al., 1966, 1970; Pereda et al., 2005). While additional statistical tests can be employed to increase the likelihood that there is a functional connection between the cells generating each signal, even in the event of few spikes (Shao & Tsau, 1996), it is still not guaranteed that such connection exists without additional experimental measures such as imaging. To reiterate, if two signals are not independent, there is no guarantee that one signal causes firing of the other signal (Durstewitz, 2017; Moore et al., 1966; Pereda et al., 2005). Methods to estimate causality have already been published (Awiszus, 1992; Bartram et al., 2024; Eggermont, 2014; Gerstein & Perkel, 1972; Hu et al., 2022; Ito et al., 2014; Kobayashi et al., 2019; Melssen & Epping, 1987; Moore et al., 1966, 1970; Narayanan & Laubach, 2009; Perkel et al., 1967; Saberi-Moghadam et al., 2018; Shao & Tsau, 1996; Wang et al., 2024) and are therefore not addressed here.

In the algorithm, a chi-squared test is used to determine whether a correlogram is uniform or nonuniform (Hristova & Wimley, 2023; Pearson, 1992; Shao & Tsau, 1996). The chi-squared test is ideal for binned distributions such as correlograms, as opposed to the Kolmogorov–Smirnov test for continuous distributions (Hristova & Wimley, 2023; Pearson, 1992). Note that, while the Kolmogorov–Smirnov test can be modified for discrete distributions, MATLAB’s built in Kolmogorov–Smirnov test functions assume continuous distributions (MATLAB Help Center, 2006b, 2006c). Therefore, the chi-squared test was employed instead of Kolmogorov–Smirnov. For a correlogram generated using $S$ comparison signal events, with bin centers $x$, a total of $L$ bins, and bin counts normalized to obtain probability $C(x)$, the correlogram is uniform if $C\left(x\right)=1/L$ for all $x$. The chi-squared statistic (${\chi }^{2}$) has $L-1$ degrees of freedom, and is calculated according to Eq. 1 (Hristova & Wimley, 2023; Pearson, 1992).

1 $${\chi }^{2}=\sum_{x=1}^{L}{\left(S \left[C\left(x\right)-\frac{1}{L}\right]\right)}^{2}$$

The *p*-value to determine whether $C(x)$ is significantly different from a uniform distribution can then be obtained by substituting the degrees of freedom and ${\chi }^{2}$ into the chi-squared cumulative distribution (*p* = *chi2cdf(*${\chi }^{2}$*, L-1, ‘upper’)* in MATLAB (MATLAB Help Center, 2006a)). A correlogram is classified as uniform if the *p*-value is larger than a user-specified threshold such as 0.05, or nonuniform if the *p*-value is less than or equal to the user-specified cutoff. Figures generated by the algorithm display both the *p*-value and the uniformity classification for each correlogram.

#### Quantifying and Classifying Firing Pattern

Variation in the firing pattern of two signals can be assessed by counting the number of peaks in each correlogram, with more peaks indicating more complex and varied patterns (Gerstein & Perkel, 1972; Moore et al., 1970; Perkel et al., 1967). For example, assuming the reference signal fires only at one time point, the correlogram of a comparison signal that fires in a similar manner will have only a single peak (Moore et al., 1970; Perkel et al., 1967). More variation in interspike interval results in more correlogram peaks (Moore et al., 1970; Perkel et al., 1967). However, peak count does not always directly correspond to the number of interspike intervals (Moore et al., 1970; Perkel et al., 1967). Peak count is therefore a measure of pattern diversity, where more peaks indicate more diverse patterns (Moore et al., 1970; Perkel et al., 1967). The algorithm uses the following procedure to count correlogram peaks. To reduce noise, correlograms are first smoothed via quadratic regression over a user-specified window $W$ (*smoothdata(*$C(x)$*,'loess', *$W$*)* in MATLAB). Peaks are then identified from the smoothed correlogram via MATLAB’s *findpeaks* function. Each peak must have a prominence greater than $1/L$ to be considered a peak. In other words, only peaks that stand out more than the peaks from a uniform distribution are considered. Once peaks in the correlogram are identified, the number of peaks and peak times relative to $t=0$ are saved for each correlogram.

Correlogram peak counts are classified based on a user-specified maximum for ease of data analysis and viewing. For example, if the highest peak count for all correlograms in a given dataset is 10, the user can choose a maximum value less than 10, such as 4. Correlograms will then be classified as having 0, 1, 2, 3, and 4 or more peaks, where 4 is the user-determined threshold. Peak times, located at $t$ s, are also classified by the algorithm. Both peak count and peak timing classifications are generated as output. Peak timing is classified by the absolute value of $t$ ($\left|t\right|$) or by frequency ($f=1/\left|t\right|$), as desired by the user. If peak locations are classified by $\left|t\right|$, peak times are classified into timescales: μs, ms, s,… based on the correlogram range. For example, if a correlogram ranges from $\pm$ 1 s in 1 ms bin widths, peak times will be sorted into four bins: $\left|t\right| \le$ 1 ms, 1 ms $<\left|t\right| \le$ 10 ms, 10 ms $< \left|t\right| \le$ 100 ms, and 100 ms $< \left|t\right| \le$ 1 s. This log separation for the classifications was used to avoid biasing classifications to large or small times. If peak locations are classified by $f$, $f$ is classified into EEG frequency bands with: the delta band (< 4 Hz), theta band (4–7 Hz), alpha band (8–12 Hz), beta band (13–30 Hz), and gamma band (> 32 Hz) (Nayak & Anilkumar, 2025). Note that this classification has a slightly different interpretation than EEG frequency bands. Classifying correlogram peaks by frequency in this algorithm is not the same as rhythmic activity, and therefore not directly comparable to EEG signals. However, rhythmic activity can be identified from frequencies that are multiples of one another. For example, if there are four correlogram peaks with $f=1/\left|t\right|=$ 5 Hz, 10 Hz, 15 Hz, and 20 Hz, there is rhythmic activity at a frequency of 5 Hz, which would correspond to the theta band of an EEG signal (Nayak & Anilkumar, 2025). However, this is not the first article to employ such frequency grouping (Ito et al., 2014; Rogers & Gross, 2019). Note that these previous studies used correlograms to analyze EEG frequency bands from MEA recordings, and EEG frequency bands are therefore not exclusive to EEG signals (Ito et al., 2014; Rogers & Gross, 2019).

#### Quantifying and Classifying Firing Order

How much of the correlogram located left and right of $t=0$ provides information about firing order (Eggermont, 2014; Gerstein & Perkel, 1972; Moore et al., 1970). If the comparison tends to fire before the reference, correlogram bin counts are higher left of $t=0$, and the correlogram is therefore shifted to the left (Eggermont, 2014; Gerstein & Perkel, 1972; Moore et al., 1970). Similarly, if the comparison tends to fire after the reference, correlogram bin counts are higher right of $t=0$, and the correlogram is therefore shifted to the right (Eggermont, 2014; Gerstein & Perkel, 1972; Moore et al., 1970). If there is equal probability of the comparison firing before the reference and the reference firing before the comparison, the correlogram is centered around $t=0$ (Eggermont, 2014; Gerstein & Perkel, 1972; Moore et al., 1970). Therefore, firing order of each signal pair can be assessed by determining where a correlogram is distributed relative to $t=0$ (Eggermont, 2014; Gerstein & Perkel, 1972; Moore et al., 1970). To assess firing order, the algorithm calculates correlogram area left of zero ($A$) according to Eq. 2.

2 $$A=\sum_{x<0}C\left(x\right)$$

Since $C(x)$ is normalized by the sum of all bin counts, $\sum C\left(x\right)=1$, and $0\le A\le 1$. If $A=1$, the comparison signal always fires before the reference signal. If $A=0$, the comparison signal always fires after the reference signal. If $A=0.5$, the correlogram is centered around $t$ = 0, and neither signal has a tendency to fire before the other. For example, if signal $i$ fires, then $j$, then $i$ again, the correlogram of $i$ and $j$ will have $A=0.5$ because there is no consistent leader or follower. The further $A$ is from 0.5, the more likely one signal consistently leads while the other follows. For example, if a signal leads 70% of the time before a drug is administered ($A=0.7$), and 90% of the time after ($A=0.9$), firing order is more consistent after the drug. More consistent firing order means stronger leader/follower dynamics between the two signals, even if the signals are not directly connected.

To classify leader/follower strength, it is important to note that the correlogram of signal $i$ as a comparison vs. signal $j$ as a reference will be mirrored about $t$ = 0 by the correlogram of signal $j$ as a comparison vs. signal $i$ as a reference. For example, if $A=0.85$ for the correlogram of $i$ vs. $j$, the correlogram of $j$ vs. $i$ will have $A=0.15$. In other words, for any leader/follower pair, two correlograms exist, where ${A}_{j,i}=1- {A}_{i,j}$, and these two correlograms should be assigned the same strength classification. Therefore, leader/follower strength of a correlogram is classified into five groups based on how likely two signals are to occur in a consistent order. These groups are:

(i) weak if firing order is simultaneous or inconsistent, $0.4<A<0.6$,
(ii) fairly weak if $0.6\le A<0.7$ or $0.3<A \le 0.4$,
(iii) intermediate if $0.7\le A<0.8$ or $0.2<A \le 0.3$,
(iv) fairly strong if $0.8\le A<0.9$ or $0.1<A \le 0.2$
(v) strong if signals fire in a consistent order (or $A\ge 0.9$ or $A\le 0.1)$.

### Algorithm Pipeline

All recordings pass through the same pipeline in the script. A schematic of this pipeline is shown in Fig. 2. First, the recording is loaded using MATLAB’s readPLXFileC function (Kraus, 2024). Currently, only Plexon Inc. (.plx) files are loaded by the script. Signals are identified and rasters are obtained from the .plx file. If the user wishes to analyze data from different MEA recording software or different types of neuronal recordings, the user will need to adapt the code to load the different file type and extract the raster. Next, the user is guided through a series of prompts to clean the recording if applicable. The first series of prompts is optional and allows the user to exclude regions of the recording from analysis to eliminate effects caused by the user, not an experimental treatment. For example, in MEA recordings, a user manually selects units at the beginning of the recording (Iannello et al., 2024). This process can be lengthy, and the addition or removal of signals can cause population dynamics to fluctuate, even though such fluctuations do not originate from the cells. Any activity recorded during signal selection should therefore be disregarded and can be removed via the first series of algorithm prompts. The second series of prompts is also optional and allows the user to identify and eliminate signals in the recording to test how the particular signal contributes to population dynamics, or to eliminate sparse or noisy signals. Second, the user is guided through another series of prompts that allow the user to indicate and label any treatments administered to the culture during or before the recording and select regions of the recording in which to generate correlograms, termed analysis regions. For analysis region selection, the user can manually select and label specific recording regions, or automatically generate regions of constant, user-specified, size throughout the recording. Third, the script generates outputs independent of correlograms, including: a raster plot, firing count histograms, interspike interval histograms, and average spike rate through time. Fourth, correlograms with user-specified parameters are generated for each region of the recording. For the data presented in this article, correlograms ranged $\pm$ 1 s around $t=0$ and contained 2,001 bins, each with a width of 1 ms. The algorithm automatically normalizes correlograms to obtain probability by dividing the count in each bin by the sum of all bin counts. The user also specifies the minimum number of events (product of the reference and comparison spike count) for each correlogram to be included in data analysis. If a correlogram is constructed with less than the user-specified minimum number of events, the correlogram is considered sparse, and the metrics for the given correlogram will not be analyzed. For the data in this article, all correlograms were included in analysis unless the correlogram was empty (*i.e.* contained zero events). For correlograms that are not excluded from analysis, the algorithm calculates correlogram uniformity, peak count, and area left of zero as described in Sect. "Correlogram Shape Quantification". The user has the option to plot heatmaps of these metrics for all correlograms in each analysis region. Fifth, the algorithm plots correlogram metric distributions across analysis regions. Sixth, the algorithm classifies correlograms based on uniformity, peak count, and area left of zero (see Sect. "Correlogram Shape Quantification") and plots classification results. Seventh, the script plots how likely correlograms of a given classification were to change or remain the same classification throughout the recording. Eighth, the script combines all classifications and plots how overall correlogram shape evolved between treatments or across analysis regions. For algorithm step five through eight, the user has the option to analyze only crosscorrelograms or include autocorrelograms in the analysis as well. A summary of algorithm parameters controlled by the user is provided in Table 1.

**Table 1:** Summary of variables under user control. The user controls a variety of parameters in the MATLAB algorithm. The left column describes each parameter. Notes on parameter effects are in the right column. Table subheadings indicate parameter function in the script

***Correlogram Parameters (Essential)***	
*Parameter*	*Notes*
*Correlogram range:* For this article, the range was $\pm$ 1 s (Sect. "Correlogram Shape Quantification")	The minimum and maximum bin of the correlogram. A bigger range will capture dynamics at longer time scales.
*Number of correlogram bins*: For this article, number of bins was calculated from the desired bin width of 1 ms (Sect. "Correlogram Shape Quantification")	If the user wishes to ensure that correlogram bins have a specific width, the user can calculate the number of bins by dividing the correlogram range by half the desired bin width. A smaller bin width will result in more correlogram bins and capture more dynamics at fast timescales, but will increase the risk of sparse correlograms.
*Analysis Region Duration:* Time periods in the recording from which to generate correlograms	Prompts allow the user to manually or automatically select analysis regions. If the user is manually selecting analysis regions, region duration is the difference between the region end indicated by the user and the region start indicated by the user. If the user is not manually selecting analysis regions, the algorithm automatically divides the recording into regions of a fixed duration specified by the user. For this article, analysis region duration was 7 min unless otherwise specified (Sect. "Correlogram Shape Quantification"). Longer regions will contain more spikes with which to generate correlograms. Therefore, longer region durations will decrease the likelihood of sparse correlograms, but increase computational complexity (Sect. "Computational Complexity").
***Correlogram Classification Parameters (Essential)***	
*Parameter*	*Notes*
Minimum number of entries in the correlogram before the correlogram is considered for analysis	This parameter can be used to exclude sparse correlograms. If the user wishes to include all correlograms regardless of spike count, set this number to 0. Otherwise, this threshold can be set based on visual inspection of correlograms (see Sect. "Correlogram Shape Quantification" for how to determine this threshold and "Optional Plots of Correlogram Metrics" in this table).
Option to include or exclude autocorrelograms from the violin plots of correlogram metrics and metric classification	User preference
The Chi Squared *p*-value below which a correlogram is considered nonuniform. For this article, the *p*-value was 0.05 (Sect. "Quantifying and Classifying Signal Dependence")	This parameter will affect how many correlograms are classified as uniform/nonuniform. Lower values will mean fewer nonuniform correlograms, but decreased likelihood of mistaking a uniform correlogram as nonuniform.
Smoothing window size to reduce noise in correlogram shape before locating correlogram peaks (Sect. "Quantifying and Classifying Firing Pattern")	Increasing this smoothing window size will decrease peak count, but be less likely to mistake noise in correlogram shape as a peak. If no smoothing is desired, set this parameter to 1.
Maximum peak count for peak count classification (Sect. "Quantifying and Classifying Firing Pattern")	For correlogram peak classification (Sect. "Quantifying and Classifying Firing Pattern"), the user classifies number of peaks up to this user-specified threshold. For example, if the grouping is: 0, 1, 2, 3, 4 or more peaks, this threshold was 4.
Option to classify peak times according to $\left|t\right|$ or $f=1/\left|t\right|$. Peak times will be classified regardless. This option just allows the user to control how (Sect. "Quantifying and Classifying Firing Pattern")	User preference
Option to create correlograms using only a subset of the signals as reference signals	For most applications, this option will not be needed, and all signals will be used as references. However, this option will be useful for analyzing perievent histograms or targeted crosscorrelation analysis.
***Optional Plots of Correlogram Metrics***	
*Optional Plot*	*Notes*
Option to plot heatmaps of correlogram uniformity and the Chi Squared *p*-value	One heatmap is generated for each analysis region. Therefore, to control the number of output figures, the algorithm only generates these heatmaps if desired by the user. Heatmaps are shown in Fig. 6B-D and Supplementary Movies S1-S9
Option to plot heatmaps of correlogram peak count	
Option to plot heatmaps of correlogram area left of zero	
Option to plot the difference in uniformity, peak count, and area left of zero for a correlogram between one analysis region and the next	This option applies to heatmaps and violin plots of correlogram metrics
Option to plot correlograms of specific signal pairs	Allows the user to visualize specific correlograms if desired (Fig. 2C and 2) **This option should be used for at least a few correlograms in each new recording to ensure correlograms are not sparse.**
***Controls for Comparing Correlograms in Different Regions***	
*Parameter*	*Notes*
Option to compare correlograms region by region, or by before vs. after experimental treatments	User preference
***Data Loading during Reprocessing*** *These parameters only apply if the user has already run the algorithm on a given recording and wishes to alter settings from the previous run. These options always run the first time a recording is analyzed.*	
*Option*	*Notes*
Option to ignore user-specified sections of a recording	The ignored section of the recording will not contribute to any algorithm outputs (Sect. "Algorithm Pipeline").
Option to delete specific signals	The ignored signal(s) will not contribute to any algorithm outputs (Sect. "Algorithm Pipeline").
Option to reset analysis regions if the user desires	If this option is used, the algorithm remakes analysis regions and reprocesses the correlograms with the new regions.
Option to relabel experimental treatments if the user desires	This option should be employed if the user mislabeled or forgot to label experimental treatments in previous runs of the algorithm.
Option to reprocess the recording (from raster plotting through correlogram creation and metric outputs)	This option should be employed if the user wishes to change correlogram settings (see "Correlogram Parameters" section of this table) compared to the previous run of the algorithm.
Option to recalculate correlogram metrics	This option should be employed if the user wishes to change correlogram classification settings (see "Correlogram Classification Parameters" section of this table), but keep the same correlogram bin count and range compared to the previous run of the algorithm.

### Statistics

The algorithm visualizes correlogram metric distributions with violin plots. The user can identify changes in metrics across analysis regions by changes in violin shape (Hintze & Nelson, 1998; Tanious & Manolov, 2022; Weissgerber et al., 2019). Violin plots are widely used to visualize data distributions (Hintze & Nelson, 1998; Tanious & Manolov, 2022; Weissgerber et al., 2019). Differences in violin shape are often used to indicate changes that arise due to different experimental conditions, but can also be used to study changes in metrics measured from a single individual or specific group of individuals through time (Hintze & Nelson, 1998; Tanious & Manolov, 2022; Weissgerber et al., 2019). If the plot is generated from a large sample size, even subtle differences in violin shape can be significant (Hintze & Nelson, 1998; Tanious & Manolov, 2022; Weissgerber et al., 2019). However, if the plot is generated from a smaller sample size, more dramatic changes in violin shape are needed for significance, and more attention should be paid to the individual data points rather than the violin (Hintze & Nelson, 1998; Tanious & Manolov, 2022; Weissgerber et al., 2019). The more measurements used to create the violin, the more reflective violin shape is of the true data distribution (Hintze & Nelson, 1998; Tanious & Manolov, 2022; Weissgerber et al., 2019).

It is important to consider the number of recorded signals when determining changes from violin shape. To help in this endeavor, the algorithm in this article uses MATLAB’s *violinplot* function (Bechtold, 2016) to show correlogram metric distributions. This was a deliberate choice because *violinplot* also displays dot plots over the violin to show each individual value contributing to the distribution (Bechtold & Bastian, 2016). If individual dots are harder to distinguish, the user can be more confident that violin shape reflects the true data distribution. However, if each dot is easy to distinguish, the number of recorded signals, and hence sample size, is small and more care must be employed in interpreting changes in violin shape.

This article did not employ statistical testing to obtain *p*-values for any observed differences, but instead looked for differences in violin shape. However, if *p*-values are desired, the shape of a violin plot can be used to determine what statistical test to apply (Hintze & Nelson, 1998; Tanious & Manolov, 2022; Weissgerber et al., 2019). For example, if the violin is wide in the middle and symmetrically tapers to the ends, the distribution is likely normal, and tests assuming normality can be employed. If violin shape is more complex, statistical tests that best apply to the appropriate distribution, or distribution free tests like Chi Squared or Kolmogorov–Smirnov (Hristova & Wimley, 2023; Pearson, 1992; Shao & Tsau, 1996) should be employed.

### MEA Recording to Validate Algorithm Outputs

Several MEA recordings were used to validate algorithm outputs. The experimental system and primary cell culture protocols used to obtain MEA recordings have been previously detailed, and are collectively called TBI-on-a-Chip (Beauclair et al., 2024; Rogers & Gross, 2019; Rogers et al., 2022, 2023, 2025). Briefly, a mixture of neuronal cell types were isolated from the frontal cortices of murine embryos (gestational day 16, ICR mice, Inotiv (formerly Envigo), catalog number 3040 F, strain Hsd:ICR(CD-1®) E16) (Beauclair et al., 2024; Rogers & Gross, 2019; Rogers et al., 2022, 2023, 2025). Experiments and animal care were performed in compliance with the ARRIVE guidelines and the Purdue University Animal Care and Use Committee’s guidelines under institutional protocol 1306000879. Isolated cortical cells were seeded onto MEAs and cultured for three weeks in Dulbecco’s modified minimal essential medium (DMEM, Cytiva, SH30002.02) supplemented with 5% horse serum (Gibco, 26–050–088), B-27 (Gibco 17504–044), and L-glutamine (Sigma-Aldrich G8540) in 10% CO_2_ at 37 °C to allow cultures to form mature networks with spontaneous firing activity (Beauclair et al., 2024; Rogers & Gross, 2019; Rogers et al., 2022, 2023, 2025).

Reuseable and transparent MEAs were fabricated as previously described (G. W. Gross, 1979; G. W. Gross et al., 1985; G. W. Gross & Gopal, 2006). Briefly, soda-lime glass with a quartz barrier sputtered with indium-tin oxide (ITO) was photoetched to create electrode channels, then the entire grid spin insulated with polysiloxane (G. W. Gross, 1979; G. W. Gross et al., 1985; G. W. Gross & Gopal, 2006; G. W. Gross & Schwalm, 1994; Rogers & Gross, 2019). Electrode conductor terminals were then deinsulated with a single laser shot and electrolytically gold plated to decrease interfacial impedance (G. W. Gross, 1979; G. W. Gross et al., 1985; G. W. Gross & Gopal, 2006; Rogers & Gross, 2019). The resulting MEA was sterilized in an autoclave, then flamed and coated with poly-D-Lysine (Sigma-Aldrich P6407) and Laminin (Sigma-Aldrich L2020) to facilitate cell attachment (G. W. Gross & Schwalm, 1994; Lucas et al., 1986; Rogers & Gross, 2019).

To record firing activity of mature networks, cultures on MEAs were placed in a custom incubator (Beauclair et al., 2024; G. W. Gross & Schwalm, 1994; Rogers et al., 2022, 2023, 2025; Rogers & Gross, 2019). Figure 3A shows a schematic of this incubator, how culture life support was maintained, and how experimental treatments were administered for each recording. Culture pH was maintained via a CO_2_ inlet connected to a reservoir containing 15 mL of medium (Beauclair et al., 2024; Rogers & Gross, 2019; Rogers et al., 2022, 2023, 2025). To ensure adequate gas exchange, the medium flowed between the reservoir and incubator via a peristaltic pump (Beauclair et al., 2024; Rogers & Gross, 2019; Rogers et al., 2022, 2023, 2025). Chemical treatments applied to the culture during a recording were administered via a drug injection port connected to the medium flow (Beauclair et al., 2024; Rogers & Gross, 2019; Rogers et al., 2022, 2023, 2025). Alternatively, chemical treatments were administered to the medium reservoir if the objective was to standardize culture firing, and drug effects were not the experimental treatment being assessed. Culture temperature was maintained at 37 °C via resistive heating. For additional details about the MEAs and recording setup, see previous publications (Beauclair et al., 2024; G. W. Gross, 1979; G. W. Gross et al., 1985; G. W. Gross & Gopal, 2006; Rogers et al., 2022, 2023, 2025; Rogers & Gross, 2019).

Recordings were performed and saved via Plexon Inc.’s real-time acquisition system programs for unit timing in neuroscience software with the multi-channel neuronal acquisition processor hardware (MNAP, also called Harvey Box). Spikes in the recorded voltage were identified as firing events if spike voltage was at least three times higher than the noise floor (Beauclair et al., 2024; Rogers & Gross, 2019; Rogers et al., 2023, 2025). Separate signals on the same electrode were discriminated based on the waveform of the voltage change as previously described (Beauclair et al., 2024; Rogers & Gross, 2019; Rogers et al., 2023, 2025). In each minute of the recording, a unit was considered active if at least 10 spikes occurred (Beauclair et al., 2024; Rogers & Gross, 2019; Rogers et al., 2023, 2025). Each unique signal corresponds to a unique neuron (Beauclair et al., 2024; Rogers & Gross, 2019; Rogers et al., 2023, 2025). Data from a single MEA recording were saved as a .plx file that contained the rasters of each active neuron. An example raster from a single recording is shown in Fig. 3B. The algorithm is designed to load a. plx file, read the rasters, then follow the pipeline described in Sect. "Algorithm Pipeline" to generate and quantify correlograms. While this algorithm is designed to read. plx files, the same pipeline can be applied to rasters of any file type. Users will need to modify the code to read rasters if a different file type is used. Example crosscorrelograms of two signals (arrows in Fig. 3B) generated by the MATLAB script from regions of the raster near the start and end of the recording are shown in Fig. 3C and D, respectively. Plotting correlograms like those in Fig. 3C and D is an optional output of the MATLAB code controlled via the main analysis function. Three MEA recordings that measured firing in separate cultures subjected to different treatments were used to validate algorithm outputs. Duration of these three recordings was selected based on the amount of time needed for cells to respond to each experimental treatment. Variation in recording duration is common and will have no bearing on correlogram outputs as long as the algorithm’s analysis regions are much shorter than the recording duration.

The first MEA recording used for validation was a control to ensure that algorithm metrics were sensitive to changes in network dynamics when signals are not lost, but firing dramatically alters. A 1.7 h recording during which a culture was administered 40 μM bicuculline methiodide (Sigma-Aldrich 14343) via the drug injection port shown in Fig. 3A was used to determine whether the three correlogram metrics (uniformity, peak count, and area left of zero) could capture changes in neuronal network dynamics as cell firing synchronizes and exhibits seizure-like activity (G. Gross, 1995). Note that both the irregular phase (increased spiking) and bursting phase (rhythmic, synchronized bursts of activity in all recorded signals) of a seizure (De Curtis & Avoli, 2016) are reproduced in culture firing after bicuculline treatment (G. Gross, 1995). Bicuculline is a competitive antagonist of GABA_A_ receptors, and administration of the drug therefore deactivates inhibitory GABA synapses within a culture (Curtis et al., 1971; Johnston, 2013; Stanford et al., 1995). Bicuculline is also employed often in MEA recordings to standardize cell firing patterns by reducing variability in cell firing (G. Gross, 1995; G. W. Gross et al., 1997) and ensuring high population activity levels (Gramowski et al., 2006; Meyer et al., 2009). The sampling frequency of the bicuculline recording was 40 kHz, total gain was 10,000 for all electrodes, and 50 unique signals were recorded.

The second MEA recording used for validation was a control to ensure that algorithm metrics were sensitive to changes in network dynamics when signals are lost in the presence of a stressor. A 20.5 h recording during which CO_2_ flow to a culture’s life support system (Fig. 3A) was deactivated to cause cell death by pH shock was used to determine whether code outputs could capture changes in firing dynamics as a result of cell death. Culture pH at the end of the pH shock recording was 8.58 (measured by a Fisherbrand™ accumet™ XL250 Benchtop pH/ISE Dual Channel Meter). The sampling frequency of the pH shock recording was 40 kHz, and 34 unique signals were recorded. Total gain was either 7,000 or 10,000. One electrode exhibited greater noise than the others, so the gain of this electrode was reduced to 7,000 in order to avoid amplifying the noise. Total gain was 10,000 for the other, less noisy electrodes.

The third MEA recording used for validation was a control to ensure that algorithm metrics do not show false changes when network dynamics remain constant. A 6 h recording during which tangential acceleration injury was administered to the culture via an impact pendulum (Rogers & Gross, 2019) was used to determine whether code outputs matched expected changes in firing dynamics as a result of physical cell injury. Damage was administered via an impact pendulum (Rogers & Gross, 2019; Rogers et al., 2022, 2023, 2025) mid-recording by subjecting the culture to five 250 g impacts for a total accelerational force of 1,250 g as previously described (Rogers & Gross, 2019). The impact culture was also treated with 40 μM bicuculline methiodide via the medium reservoir (depicted in Fig. 3A) before the recording began in order to standardize cell firing patterns (G. W. Gross et al., 1997; Rogers & Gross, 2019). Analysis of an impact recording without bicuculline can be found in Supplementary Section S1. The sampling frequency of the impact recording was 40 kHz, total gain was 10,000 for all electrodes, and 69 unique signals were recorded (Rogers & Gross, 2019). This impact recording has already been published, and additional details can be found in the previous publication (Rogers & Gross, 2019). The recording is reused here with permission. To load the culture into the impact pendulum and administer the five impact injuries, it was necessary to briefly disconnect the culture from the recording schematic diagrammed in Fig. 3A, and signals were therefore not recorded during administration of the impacts (Rogers & Gross, 2019). The results of the previous publication suggest that correlogram uniformity, peak count, and area left of zero should not dramatically alter throughout the recording (Rogers & Gross, 2019).

## Results

**Fig. 4:**
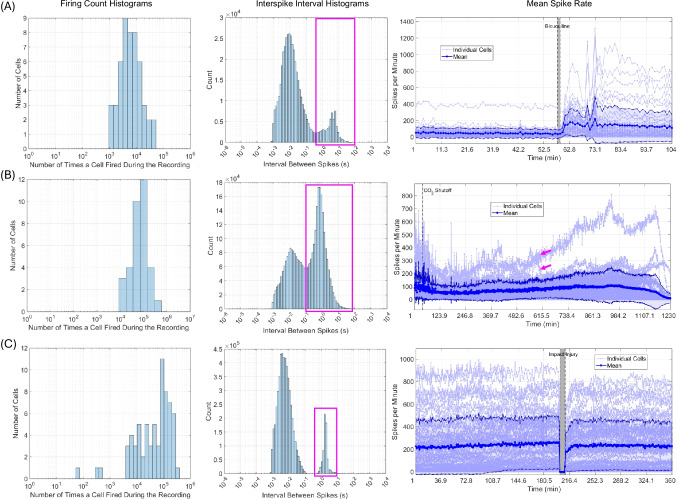
Algorithm outputs independent of correlograms. Firing count histograms, interspike interval histograms, and mean spike count per minute (dark blue line, dashed blue lines with shading in between indicate standard deviation, light blue lines are individual cells) are shown for entire extracellular recordings of neuronal cultures subjected to: 40 μM bicuculline methiodide (**panel A**), pH shock by shutting off CO_2_ flow to the culture (**panel B**), and impact injury (**panel C**). Magenta boxes indicate rhythmic activity intervals. Magenta arrows indicate two cells that responded to pH shock with opposite changes in spike rate

**Fig. 5:**
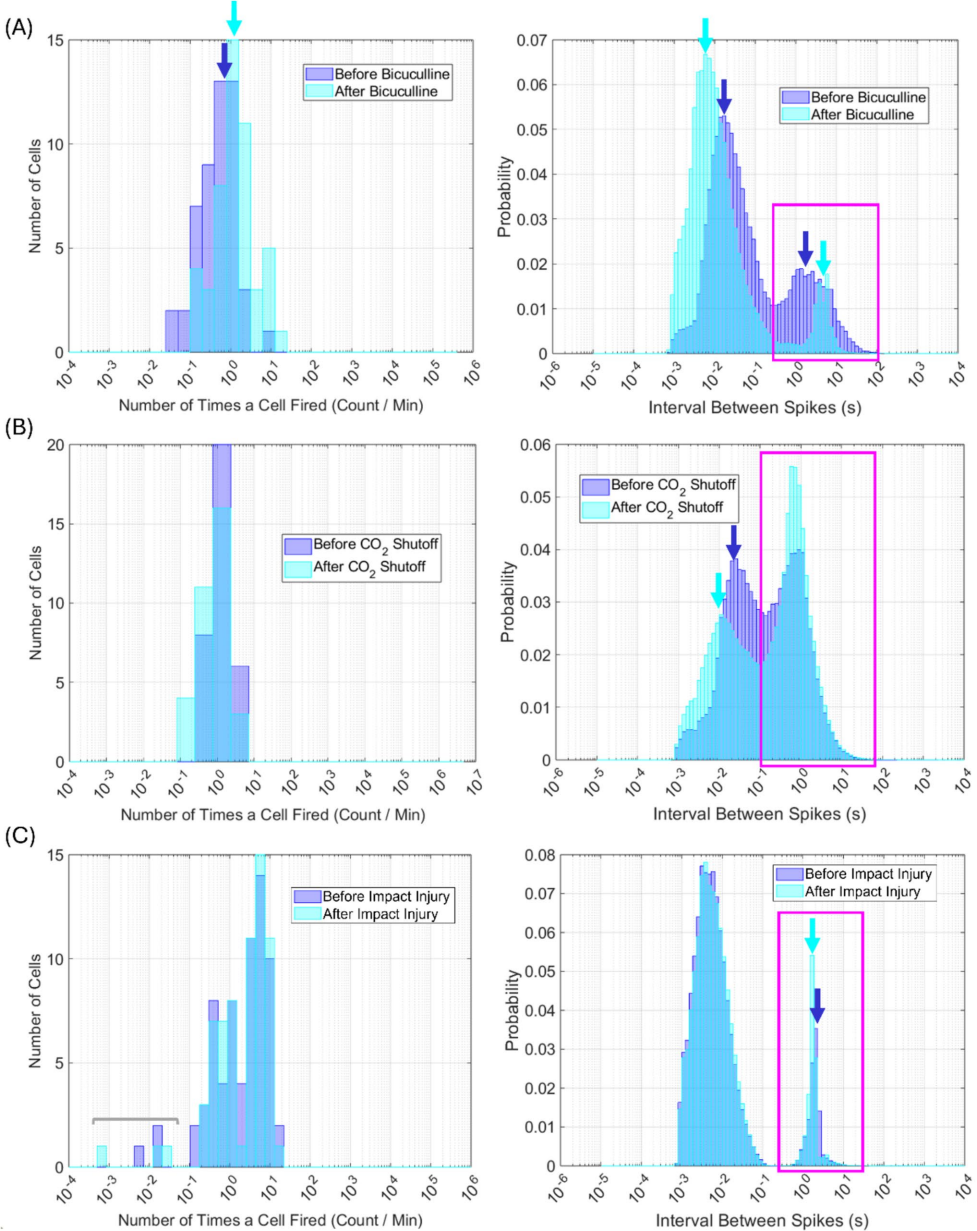
Firing count and interval histograms relative to experimental treatment. Firing count (left column) and interspike interval (right column) histograms obtained from the rasters of cells in microelectrode array recordings before and after 40 μM bicuculline (**panel A**), pH shock (**panel B**), and impact injury with bicuculline (**panel C**) are shown. Firing counts are normalized by the duration of the region (before vs. after treatment) in order to account for recording regions of different duration. Interspike interval histograms are normalized to probability so different region durations do not alter histogram shape. Blue and cyan bars correspond to distributions before and after treatment, respectively. Magenta boxes indicate rhythmic activity intervals. Dark blue and cyan arrows indicate mean values before and after treatment, respectively. Grey bars indicate changes that occurred only in a subset of cells

**Fig. 6:**
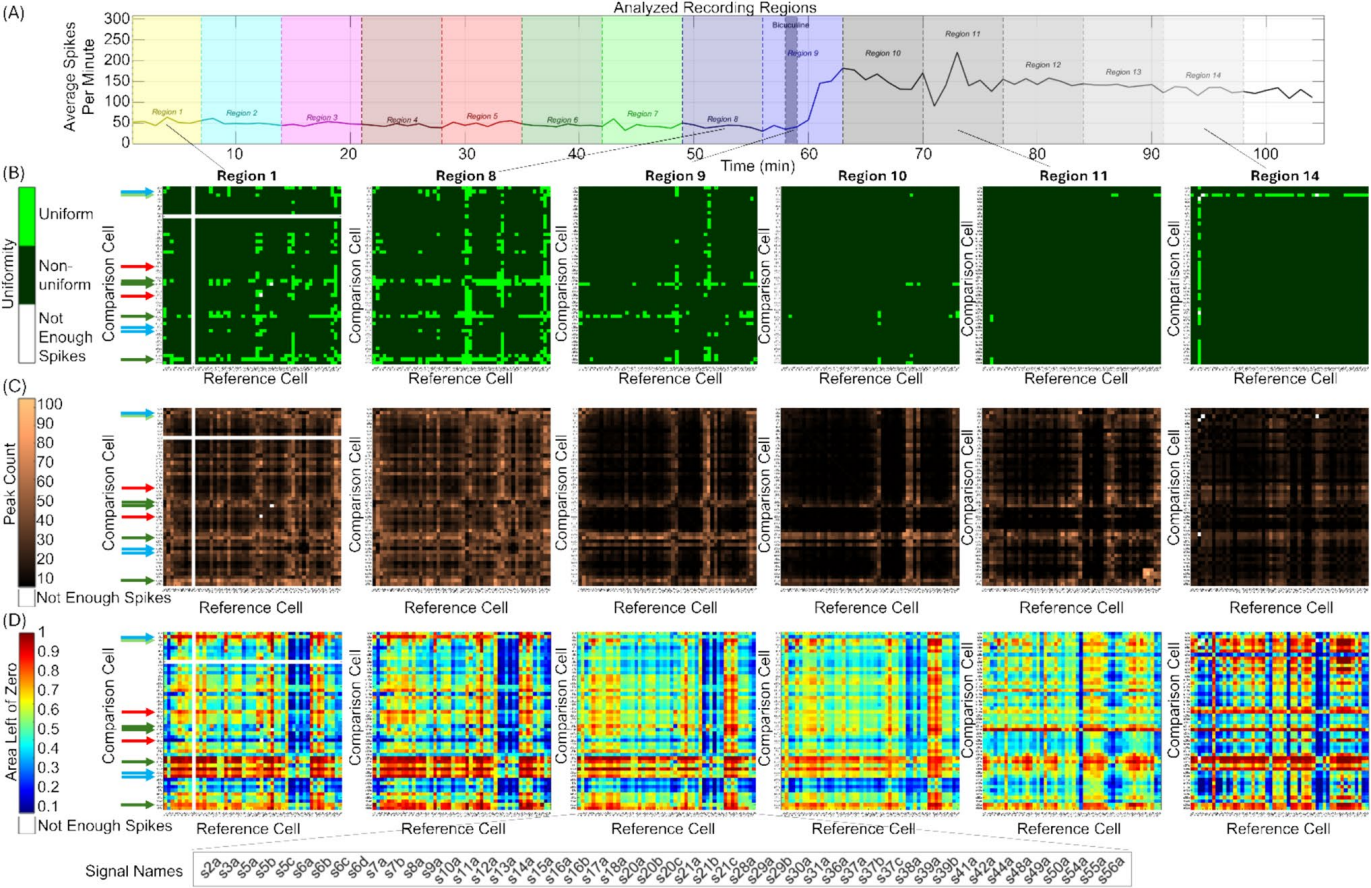
Metric heatmaps for all correlograms in a recording of cells treated with 40 μM bicuculline methiodide. Bicuculline was administered to a culture after almost one hour of recording native firing activity. The average number of spikes each minute of the recording is shown in **panel A**. The recording was divided into 14 analysis regions, each seven minutes long, also shown in **panel A**. Correlograms for each unique pair of cells were created for each analysis region. Representative heatmaps for six of the 14 analysis regions indicating which correlograms are uniform (light green) and nonuniform (dark green) across analysis regions are shown in **panel B**. Dark green arrows indicate signals that were independent before bicuculline and dependent after. Light green arrows indicate signals that became independent after bicuculline treatment. Corresponding heatmaps showing correlogram peak count and area left of zero are shown in **panel C and D**, respectively. For peak count, a brighter color indicates more peaks. For area left of zero, a value of 1 (red) indicates that the comparison cell (y-axis) always fired before the reference cell (x-axis). A value of 0 (blue) indicates that the comparison always fired after the reference. A value of 0.5 (green) indicates that there is no consistent firing order between the pair of cells. Red arrows indicate signals that fired before most others only after bicuculline was administered. Blue arrows indicate cells that fired before most others, but that lost this leader behavior after bicuculline was administered. In all heat maps, white pixels indicate correlograms of cells that did not fire in the respective analysis region

**Fig. 7:**
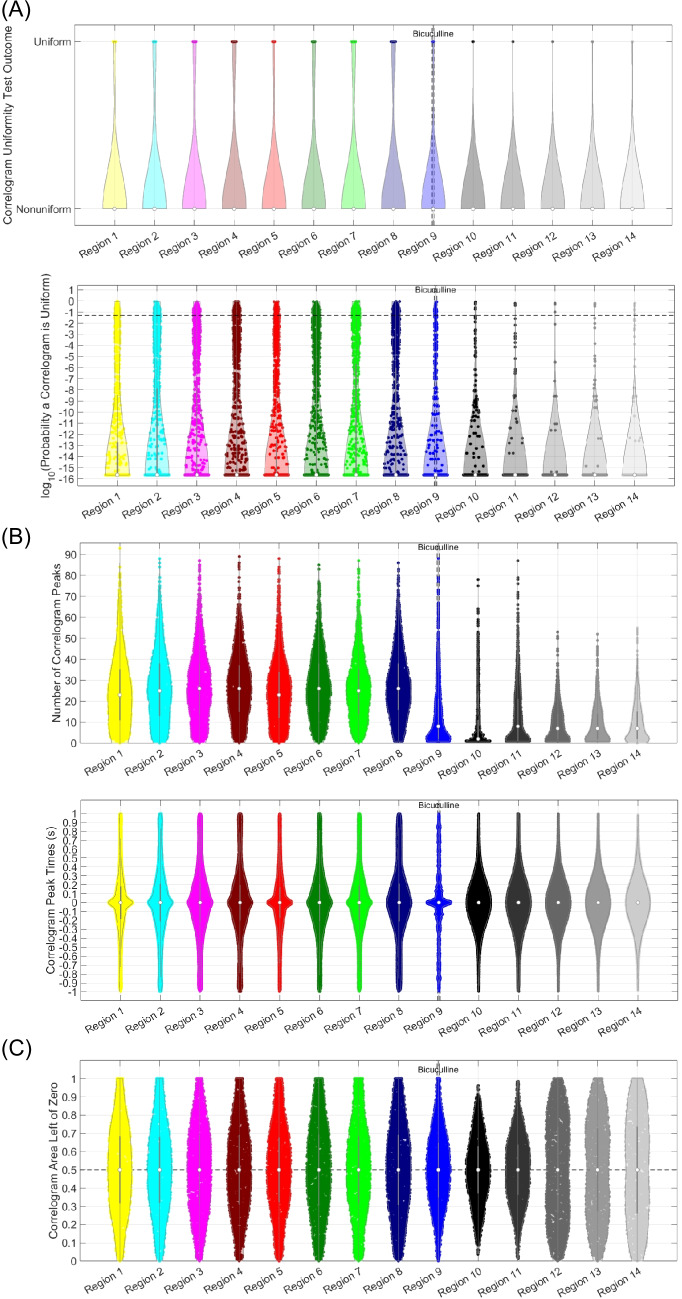
Correlogram metric distributions across analysis regions for a bicuculline-treated culture. In this recording, the culture was treated with 40 μM bicuculline methiodide during analysis region 9. Violin plots are displayed to view population level changes in correlogram properties across analysis regions (shown in Fig. 6A). Correlogram uniformity and probability of being uniform are shown in **panel A**, peak count and peak times in **panel B**, and area left of zero in **panel C**. The dashed line in panel A represents the significance cutoff of *p*-value = 0.05 (Chi squared test, Sect. "Quantifying and Classifying Signal Dependence"), used to determine whether the correlogram is uniform. Individual correlogram metrics for each analysis region are shown in the heat maps in Fig. 6 and correspond to individual points in the violins. White points in the violins represent the median metric value.

**Fig. 8:**
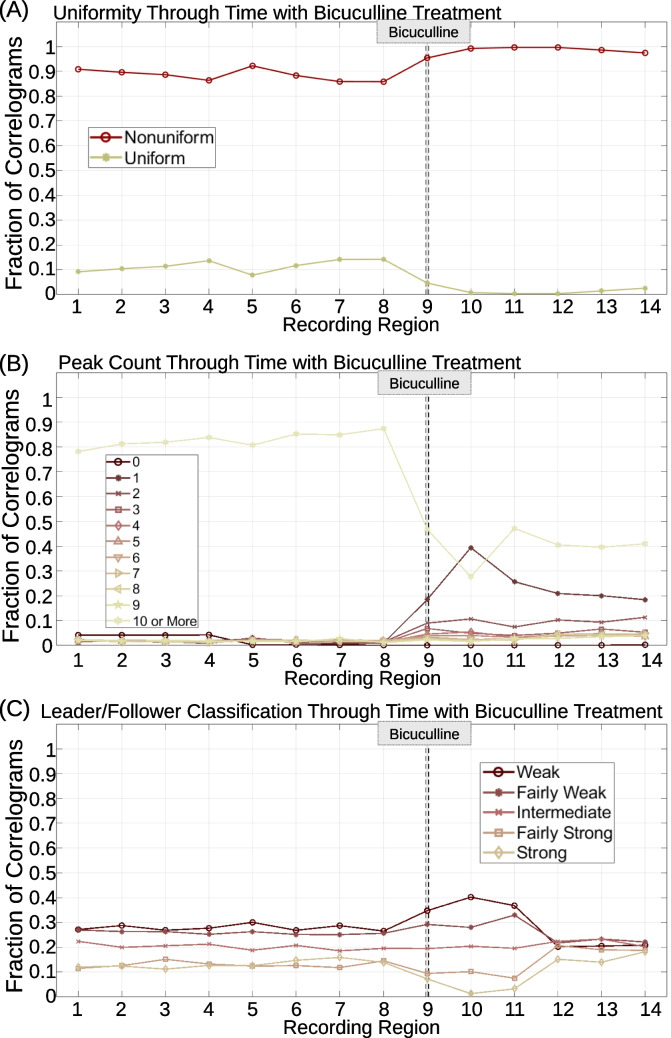
Correlogram classification summaries for bicuculline treatment. Correlogram uniformity (**panel A**), peak count (**panel B**), and leader/follower consistency (**panel C**) were classified as described in methods Sect. "Correlogram Shape Quantification". The fraction of correlograms with each classification are shown for a microelectrode array recording in which the culture was subjected to 40 μM bicuculline methiodide. Dashed vertical lines indicate when bicuculline was administered

**Fig. 9:**
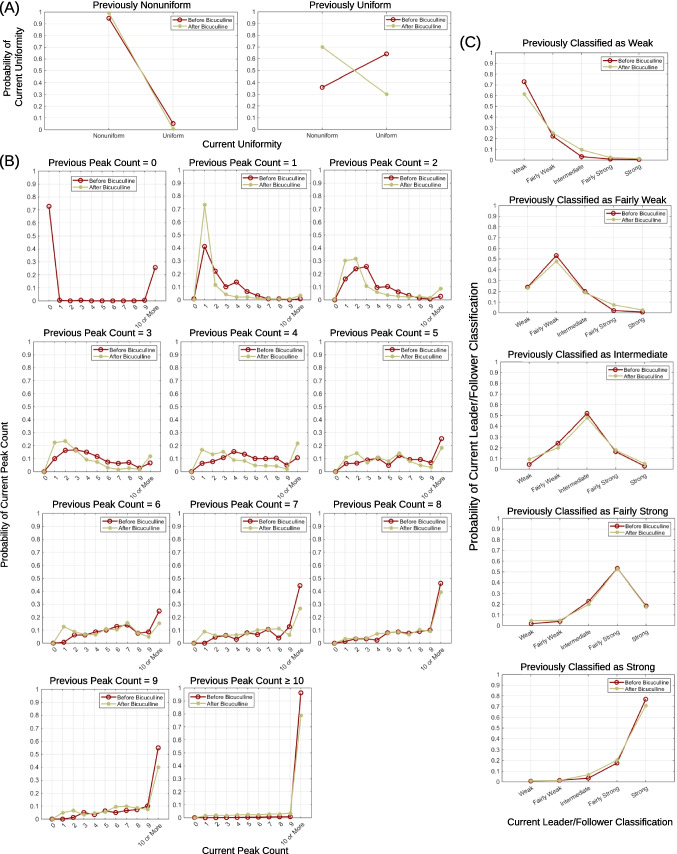
Evolution of correlogram classifications before and after 40 μM bicuculline methiodide treatment. Correlogram uniformity (**panel A**), peak count (**panel B**), and leader/follower nature (**panel C**) were classified as described in methods Sect. "Correlogram Shape Quantification". Correlograms of a specific classification may be more likely to change through time or after experimental treatments. To identify such correlograms, classifications were tracked between analysis regions (shown in Fig. 6A). For all but the first analysis region, correlograms were grouped based on the classification in the previous analysis region. For each group, a histogram of correlogram classification in the current analysis region was created. This histogram was then normalized to probability and therefore represents the probability of a correlogram exhibiting each classification given the correlogram’s classification in the previous analysis region. Correlograms more likely to change will have lower probabilities of exhibiting the same classification as the previous region (for example, uniform correlograms in the right plot of panel A). Similarly, correlograms less likely to change will have a higher probability of exhibiting the same classification (for example, nonuniform correlograms in the left plot of panel A). These results were then grouped by before vs. after bicuculline treatment.

**Fig. 10:**
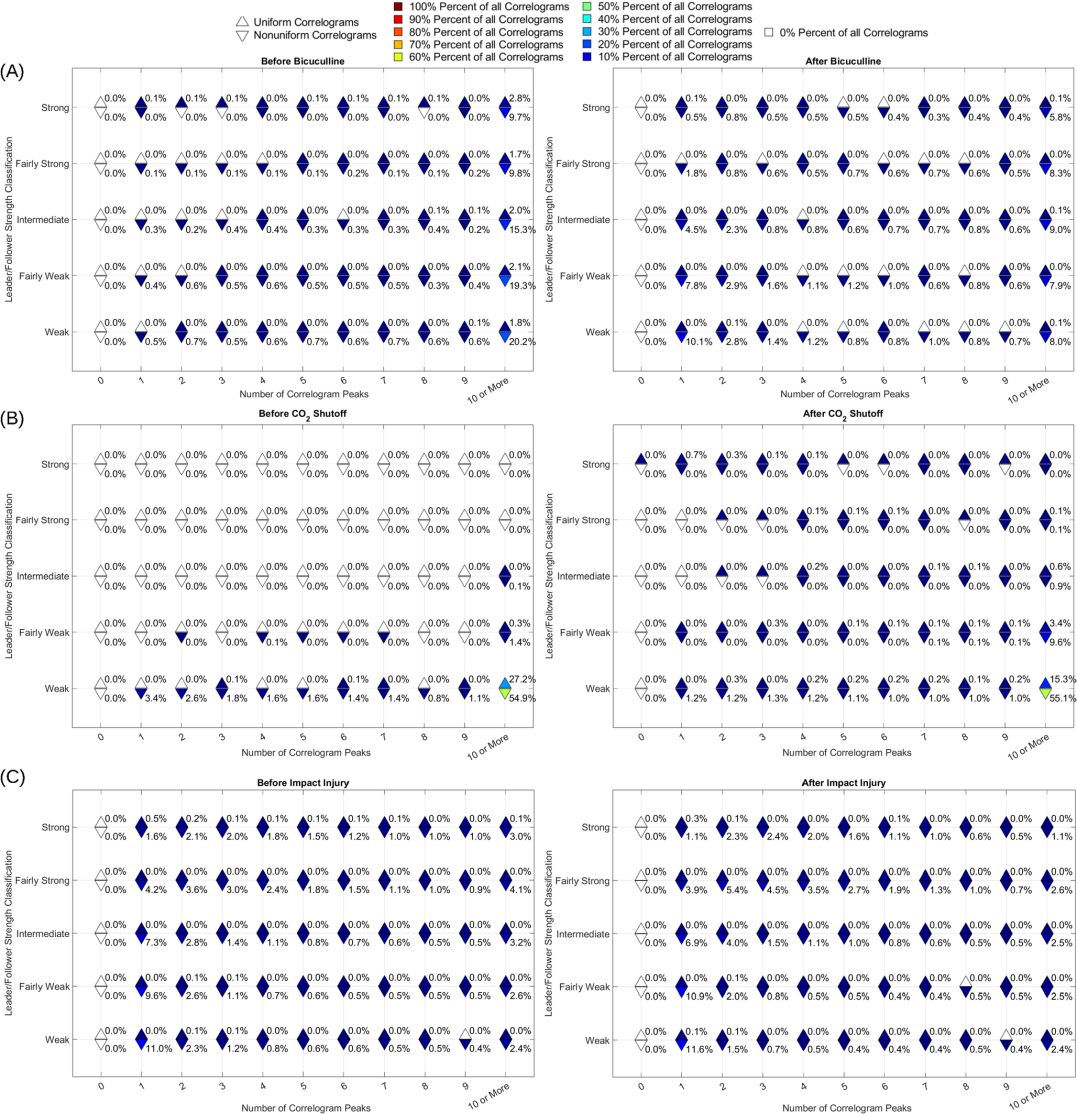
Heatmaps showing the percent of correlograms with each classification grouping for a culture before and after 40 μM bicuculline methiodide (**panel A**), pH shock (**panel B**), and impact injury under bicuculline (**panel C**). For this article, there are 110 different combinations of the two uniformity classifications, five leader/follower strength classifications, and 11 peak count classifications. Empty (white) symbols described 0% of the correlograms. Shaded symbols indicate that at least one correlogram in the culture was described by the given classification combination. Shading ranges from dark blue (> 0%) to dark red (100%). Triangles pointing up represent uniform correlograms while triangles pointing down represent nonuniform correlograms.

Each of the three recording files described in Methods Sect. "MEA Recording to Validate Algorithm Outputs" was run through the MATLAB analysis pipeline described in Methods Sect. "Algorithm Pipeline". First, raw data in a .plx file from each recording was read into MATLAB using the *readplx* function available from the MATLAB Central File Exchange (Kraus, Benjamin, 2024). From the raw data, cells were assigned a unique ID that consists of “s” followed by a number, then a, b, c, or d. In this naming scheme, “s” stands for signal, the number represents the electrode from which the signal was recorded, and a through d represent the first through fourth signal on the given electrode, respectively. A maximum of four signals were discriminable on each electrode. For example, “s2c” represents the third signal on electrode 2. Rasters (Fig. 3B) from each unique signal were first used to obtain general recording statistics (Sect. "Outputs Independent of Crosscorrelations", Figs. 4 and 5). Rasters were then used to create correlograms (Fig. 3C and D) and analyze correlogram properties (Sect. "Correlogram Creation, Uniformity, Peak Count, and Area Left of Zero"-"Correlogram Classification Heat Maps", Figs. 6, 7, 8, 9, and 10). Autocorrelograms were excluded from the distributions shown in Figs. 7, 8, 9, and 10 in order to focus on changes in interactions between cells, but can be included via a switch in the main function based on user preference.

### Outputs Independent of Crosscorrelations

The algorithm calculates several metrics independently of cross correlation analysis. Figure 4 shows distributions in the number of times each cell fired during each recording, interspike interval distributions arising from each cell, and the mean firing count per minute. Note that interspike intervals are calculated raster by raster, and thus reflect intervals between the spikes of each cell, not any spike in the recording. Comparing and contrasting these outputs against correlogram metric outputs can provide additional information to assist in understanding neuronal dynamics. Examples of how this comparison can improve metric interpretation are provided in the discussion of each recording (Sects. "Metric Interpretations for Bicuculline"-"Metric Interpretations for Impact Injury").

In all three recordings, most cells fired between 10^4^ and 10^5^ times. However, three cells in the impact recording (Fig. 4C) fired less than 1,000 times, suggesting that a subpopulation of less-active cells may have existed in the impact culture. Interspike interval distributions were bimodal for all recordings, with one peak between 0.001–0.1 s and another between 1–10 s (magenta boxes in Fig. 4). The peak at longer times corresponds to rhythmic activity such as inter-burst intervals, while the peak at shorter times corresponds to intervals between spontaneous firing events, such as refractory periods (if within a few ms (Gallistel et al., 1969)), or firing within a burst. Note that refractory period durations can be obtained from autocorrelograms (Bar-Gad et al., 2001) if desired, but such analysis is not employed in this article.

As shown in Fig. 4A, on average, cells in the bicuculline recording fired 4,500 times (from the peak in the firing count histogram). From the peaks in the interspike interval histogram, the average short interspike interval was roughly 10 ms and the average rhythmic activity interval was 0.5 s (peak in the magenta box of Fig. 4A) for the entire recording. Spikes occurred at the shorter interval roughly 3× more often than the rhythmic activity interval. From the mean spike rate plot, bicuculline increased the average number of spikes per minute shortly after the drug was administered through the end of the recording (Fig. 4A), in agreement with the scientific literature (Gramowski et al., 2006; Meyer et al., 2009).

As shown in Fig. 4B, on average, cells in the CO_2_ shock recording fired 900,000 times (from the peak in the firing count histogram). From the peaks in the interspike interval histogram, the average short interspike interval was roughly 10 ms and the average rhythmic activity interval was 1 s (peak in the magenta box of Fig. 4B) for the entire recording. Spikes occurred at the shorter interval roughly 0.5× less often than the rhythmic activity interval. Before CO_2_ shock, the mean number of spikes per minute exhibited stable fluctuations around a constant value of ~ 100 spikes per minute. Roughly 50 min after CO_2_ shutoff, the mean spikes per minute decreased to ~ 50 spikes per minute, and the fluctuations vanished. Decreased spike rate lasted for roughly six hours. After these six hours, two signals increased their firing rate. Due to these two signals, the mean spikes per minute also increased. From minute 246 of the recording on, individual cells exhibited different trends. Some cells increased firing while others decreased firing in response to CO_2_ shutoff (representative cells indicated by magenta arrows, Fig. 4B), suggesting that firing rate alterations in response to alkalosis are cell-dependent. This observation is confirmed in the literature (Sinning & Hübner, 2013). Finally, roughly 19.5 h into the recording, the mean number of spikes per minute decreased due to cell death from pH shock.

As shown in Fig. 4C, on average, cells in the impact under bicuculline recording fired 90,000 times (from the peak in the firing count histogram). From the peaks in the interspike interval histogram, the average short interspike interval was roughly 4 ms and the average rhythmic activity interval was just under 2 s (peak in the magenta box of Fig. 4C) for the entire recording. Spikes occurred at the shorter interval roughly 2× more often than the rhythmic activity interval. Impact slightly decreased the mean number of spikes per minute, and the spikes per minute of most individual cells, as previously described (Rogers & Gross, 2019).

In addition to plotting metrics throughout a recording, the algorithm also plots firing count and interspike interval histograms relative to experimental treatments. Firing count is normalized by the length of each recording period to ensure that different amounts of time recorded before and after treatment(s) do not affect the results. Similarly, interspike interval distributions are normalized to probability. Figure 5 shows firing count and interspike interval distributions before and after each experimental treatment.

The firing count histograms in Fig. 5 show that culture firing count increased after cells were treated with bicuculline (arrow and distribution position, Fig. 5A). On average, cells fired 0.7 times per minute before bicuculline (blue arrow, Fig. 5A) and slightly more than 1 time per minute after bicuculline (cyan arrow, Fig. 5A). Interspike interval distributions were also altered by bicuculline. Before bicuculline was administered to a culture, the interspike interval distribution peak corresponding to rhythmic activity (magenta boxes, Fig. 5) ranged between 0.4 s and 30 s, with a mean of ~ 2 s (dark blue arrow, Fig. 5A). After bicuculline, the rhythmic activity peak ranged between 1.5 s – 15 s, with a mean of ~ 4.8 s (cyan arrow, Fig. 5A). Narrowing of the rhythmic activity peak suggests that rhythmic firing in which most of the culture participates occurs more after bicuculline, potentially contributing to the increased spike count. The distribution peak at ms timescales decreased from ~ 17 ms before bicuculline (dark blue arrow, Fig. 5A) to ~ 6 ms after bicuculline (cyan arrow, Fig. 5A). Together, these observations suggest that bicuculline increases firing count in the culture by causing cells to fire more within a span of a few ms, and fire more rhythmically. Previous studies have observed similar effects (Gramowski et al., 2006; G. W. Gross et al., 1997; Meyer et al., 2009).

Culture firing count decreased slightly after the CO_2_ shutoff initiating pH shock (distribution shift to lower values, Fig. 5B). Alkalosis also altered interspike interval histograms (Fig. 5B). The interspike interval peak at shorter timescales ranged between 8 and 123 ms, with a mean of ~ 26 ms (dark blue arrow, Fig. 5B) before CO_2_ shutoff. After CO_2_ shutoff, the same peak ranged between 0.8 ms and 123 ms, with a mean of ~ 11 ms (cyan arrow, Fig. 5B). Peaks corresponding to rhythmic activity (magenta box, Fig. 5B) ranged between 0.12 s and 6 s, with a mean of ~ 0.66 s both before and after CO_2_ shutoff. However, before CO_2_ shutoff, both peaks had roughly the same probability, meaning that cells were equally likely to fire with shorter and longer intervals. After CO_2_ shutoff, the probability of a shorter interspike interval decreased. Therefore, while CO_2_ shutoff did not notably affect the duration of longer interspike intervals, cells were more likely to fire with longer interspike intervals after CO_2_ shutoff. Together, these observations suggest the slight decrease in cell firing count after CO_2_ shutoff is the result of increased propensity for longer interspike intervals. This hypothesis agrees with previous literature observations of firing during alkalosis (Zhang et al., 2013).

Firing count distributions remained the same after impact for all but a few cells which decreased firing (grey bracket, Fig. 5C). Interspike interval distributions were nearly identical before and after impact (Fig. 5C), likely due to the presence of bicuculline in the culture medium before, during, and after impact. The peak at ms timescales ranged between 0.8 and 123 ms, with a mean of ~ 4 ms both before and after impact. The peak corresponding to rhythmic activity (magenta box, Fig. 5C) ranged between 0.53 s and 15.2 s before and after impact. The mean value of the rhythmic activity peak decreased slightly from ~ 2.3 s before impact (dark blue arrow, Fig. 5C) to ~ 1.7 s after impact (cyan arrow, Fig. 5C). However, the probability of longer interspike intervals increased from a maximum of ~ 0.035 before impact to a maximum of ~ 0.055 after impact. Therefore, while the mean rhythmic interval decreased, cells were overall more likely to fire with longer intervals. This observation suggests that the slight decrease in mean spikes/min after impact occurred because cells had an increased propensity for longer interspike intervals.

It is important to note that the interspike interval distributions of the impact culture before and after impact resemble those of a culture after bicuculline administration (Fig. 5A and C). Since the impact culture was treated with bicuculline before the recording began (Rogers & Gross, 2019), this observation suggests that changes as a result of impact could have been obscured by the effects of bicuculline. This notion is supported by the fact that changes in the firing count and interspike interval distributions after impact follow the same overall trend, but are more pronounced after impact in cultures without bicuculline, as shown in Supplementary Section S1, Supplementary Figure S1.

### Correlogram Creation, Uniformity, Peak Count, and Area Left of Zero

The metrics shown in Figs. 3 and 4 are independent of cross-correlations, which are generated by the MATLAB script after the other recording statistics are displayed All recordings in this article were automatically divided into seven minute analysis regions in order to reduce the likelihood of sparse correlograms (longer regions mean more spikes in each region) while still obtaining fine time resolution through the recording. This automatic analysis region generation resulted in a total of 14 analysis regions for the bicuculline recording, 175 for the pH shock recording, and 50 for the impact recording. All correlograms in this article ranged between −1 s and 1 s, with a bin width of 1 ms (see Table 1). After correlogram generation, uniformity, peak count, and area left of zero were calculated and classified for each correlogram as described in methods Sect. "Correlogram Shape Quantification". Metrics were calculated for all correlograms that contained at least one raster event (see Table 1). Therefore, it is important to consider correlogram spike count when interpreting correlogram metric outputs. As detailed in the following sections, sparsity artifacts did not arise in the bicuculline recording, arose at the end of the pH shock recording when cells began dying, and appeared in some correlograms after impact.

Heat maps showing correlogram uniformity, peak count, and area left of zero can be generated as optional code output if the user wishes to assess the three metrics for each unique correlogram (see Table 1). Figure 6 shows these optional heat maps for select regions of the bicuculline recording, as well as the automatically selected analysis regions. Movies of the heat maps for the other MEA recordings are provided in the supplementary material. Signal interactions that changed most in response to treatment and changes in correlograms involving specific signals can be identified via the heatmaps. If desired, the user can also plot heatmaps showing the change in metric values between analysis regions (not shown).

For two neurons, $i$ and $j$, the correlogram of $i$ vs. $j$ is the mirror (around $t$ = 0) of the correlogram of $j$ vs. $i$. Therefore, the uniformity and peak count matrices displayed as heatmaps are symmetric, meaning that the entry at row $i$, column $j$ is the same as the entry at row $j$, column $i$ (Fig. 4B,C). For the area left of zero matrix, the entry at row $i$, column $j$ is equal to 1 minus the entry at row $j$, column $i$ (Fig. 4C). For example, if the matrix entry at row $i$, column $j$ is colored red, meaning area left of zero is close to 1, the entry at row $j$, column $i$ will be colored blue (*i.e.* the reflected area left of zero is close to 0 because 1 – [a number close to 1] is close to 0). Note that, if 0.5 is subtracted from all entries, the area left of zero matrix is skew symmetric, where the diagonal is zero and off-diagonal elements are negatives of each other. For the area left of zero matrix, a red row, blue column indicates a cell that consistently fired a signal before most other signals. Such cells fire before most others in the culture, and thus could potentially function as pacemaker cells, burst leaders, or other similar leader signals. Similarly, a blue row, red column indicates a cell that consistently fired after most other signals. A row that is partially red and partially blue suggests the signal leads some signals and follows others. Such signals may be located in the middle of a pathway or firing chain, but additional experimental data would be needed to confirm this hypothesis. Green rows indicate signals that do not consistently fire before or after other signals. However, it is important to note that these leader/follower interpretations only apply if the correlogram is not sparse, meaning a sufficient number of spikes contribute to each $C(x)$.

#### Bicuculline

As shown by the dark green arrows in Fig. 6B, most of the correlograms in which s21a, s21b, s37b, and s55a participated were uniform before bicuculline, nonuniform after, suggesting that synchronous activity induced by bicuculline (G. Gross, 1995) made these signals more dependent on others. Conversely, as indicated by the light green arrow in Fig. 6B, correlograms in which s5a participated were nonuniform before bicuculline, but became uniform after, meaning that bicuculline rendered s5a independent of most of the other signals in the culture.

As shown in Fig. 6C, bicuculline reduced the number of peaks, and increased the number of spikes (Fig. 5A). More spikes in total suggests that changes to the number of correlogram peaks are not caused by a decrease in the number of spikes contributing to each correlogram. Therefore, the decreased peak count suggests firing patterns were less diverse after bicuculline exposure, as expected from the literature (G. Gross, 1995; G. W. Gross et al., 1997).

Figure 6D shows that most correlograms maintained their position relative to $t$ = 0 before and after bicuculline. However, correlograms in which s3a, s39a and s39b (light blue arrows in Fig. 6D) acted as the comparison cell were mostly distributed left of zero before bicuculline, suggesting that these cells fired before most others in the culture. After bicuculline, the same correlograms exhibited a small area left of zero, meaning that these cells fired after others once GABAergic inhibition was lost. Conversely, s18a fired with or slightly before other cells before bicuculline, but fired before most other cells after bicuculline (Fig. 6D, red arrows). Similarly, almost all correlograms in which s29a participated as a comparison cell had a low area left of zero before bicuculline, but a high area left of zero after bicuculline (Fig. 6D, red arrows).

#### Alkalosis

Supplementary Movies S1-S3 show heat maps for the pH shock recording. Analysis regions for the pH shock recording are shown in Supplementary Figure S6A. CO_2_ shutoff occurred in analysis region 6, metric changes were noticeable around region 30–50 in the supplementary movies, then stabilized, then dramatically changed around region 155 when signals began to die. Unlike the bicuculline recording, no correlograms in this culture exhibited strong firing order, and area left of zero was close to 0.5 for most correlograms throughout the recording. The initial heat map changes (region 30–50) include a slight but temporary increase in uniform correlograms, a lasting decrease in correlogram peak count, minimal changes in firing order, and affected a majority of the correlograms. Since individual signals did not stand out in this response, additional discussion of these changes is reserved for Sect. "Violin Plots to Visualize Correlogram Metric Distributions". Just before a signal died (region 155 until the end of the recording), correlograms in which the dying signal participated often became uniform, the peak count decreased, and the area left of zero approached 1 or 0 in a seemingly random fashion. The seemingly random changes in area left of zero are likely an artifact due to sparse correlograms. With low spike counts as signals die, correlograms are too sparse to form an accurate picture of the distribution, resulting in seemingly noisy area left of zero that randomly fluctuates between 0 and 1. Together, these observations support the notion from Figs. 3B and 4B that cell firing altered when the pH shock was mild (region 30–50), and that this altered state persisted until cell death (region 155).

#### Impact Injury

Supplementary Movies S4-S6 show heat maps for the impact recording under bicuculline. Analysis of an impact recording without bicuculline is provided in Supplementary Section S1, Supplementary Movies S7-S9. Heatmap shading for all three correlogram metrics varied slightly for most correlograms as a result of impact. However, these alterations were subtle, and no dramatic changes occurred.

### Violin Plots to Visualize Correlogram Metric Distributions

While heatmaps provide detailed, signal-level information, violin plots summarize these patterns at the population level, facilitating comparison across recording regions and treatments. The heat maps in Sect. "Correlogram Creation, Uniformity, Peak Count, and Area Left of Zero" (Fig. 6, Supplementary Movies S1-S6) allow metric visualization for each correlogram throughout the recording. However, to understand metric changes at a population level, it is necessary to consider metric distributions. Width of the violins generated by the algorithm indicates metric distribution (Hintze & Nelson, 1998; Tanious & Manolov, 2022; Weissgerber et al., 2019). Violin width is calculated from a kernel density estimator, and is similar to the height of a histogram peak (Bechtold, Bastian, 2016; Hintze & Nelson, 1998; MATLAB Help Center, 2024; Tanious & Manolov, 2022; Weissgerber et al., 2019). Violin plots show the distribution of metric values, individual points (corresponding to a single entry in the heatmaps from Sect. "Correlogram Creation, Uniformity, Peak Count, and Area Left of Zero"), and the median metric value, and are created using the *violinplot* function available from the MATLAB file exchange (Bechtold, Bastian, 2016). The user can determine whether to include autocorrelograms in the distributions or consider only crosscorrelograms. For the results presented in this article, only crosscorrelograms are considered in order to focus on alterations in cell interactions. Additionally, if desired, the user has the option of plotting violins showing the change in metric value between analysis regions (current analysis region minus previous analysis region, not shown).

#### Bicuculline

Violin plots for the bicuculline recording are shown in Fig. 7. Each point in the violins corresponds to a single entry in the matrices shown in Fig. 6. The analysis regions are the same as those in Fig. 6A. As shown by the uniformity classification and *p*-values in Fig. 7A, bicuculline reduced the number of uniform correlograms.

Figure 7B shows violins representing distributions in the number of correlogram peaks, as well as locations of correlogram peaks within the correlogram range ($\pm$ 1 s). After bicuculline, the number of correlogram peaks decreased, suggesting that firing patterns between two cells were more diverse before bicuculline was administered. This observation coincides with previous findings that a dose of bicuculline comparable to that administered during the recording (35 μM in the literature, 40 μM in this study) reduces variation in the firing pattern of a culture (G. W. Gross et al., 1997). Before bicuculline, most correlogram peaks were located between −0.4 s and 0.4 s, with an additional group of peaks between 0.4 s and 1 s, and −0.4 s to −1 s. After bicuculline, fewer peaks were located between 0.4 s and 1 s, and −0.4 s to −1 s, demonstrating that cells fired over a smaller range of time after bicuculline.

Figure 7C shows violins representing the distribution in correlogram area left of zero ($A$, with $0\le A\le 1$). As previously discussed, for the two correlograms in which neuron $i$ and neuron $j$ participate, ${A}_{j,i}=1- {A}_{i,j}$. Therefore, distributions in $A$ are symmetric about 0.5. Before bicuculline, the value of $A$ for most, but not all, correlograms ranged between 0.3 and 0.7. However, a few correlograms exhibited $A<0.3$ or $A>0.7$, meaning that the culture before bicuculline exhibited relatively few pairs of $i$ and $j$ where one cell always lead or followed the other. This subset of correlograms where one cell always lead or followed the other decreased immediately after bicuculline administration (analysis region 9). This decrease lasted through analysis regions 9–11. These regions encompass the culture’s initial response to bicuculline, where culture spiking activity increased and individual cells exhibited a variety of responses to the drug (Fig. 4A). Once culture activity stabilized (starting with analysis region 12, Fig. 4A), $A$ for most correlograms ranged between 0.1 and 0.9. Therefore, in the long term, bicuculline administration resulted in more consistent firing order. For example, if a leader lead 70% of the time before bicuculline, the leader might do so 80% of the time after bicuculline.

#### Alkalosis

Violins for the pH shock recording are shown in Supplementary Figure S6. The CO_2_ supply was deactivated in analysis region 6 (red dashed line in Supplementary Figure S6A). As shown in Supplementary Figure S6B and C, most of the correlograms were nonuniform. However, correlogram uniformity was affected by pH shock twice after the CO_2_ supply was deactivated. First, correlogram uniformity increased in analysis region 30–55 (magenta boxes, Supplementary Figure S6B and C), where many correlograms become uniform, then return to the baseline uniformity value in region 56. Combined with the matrices in Supplementary Movie S1, the fraction of uniform correlograms was the same before region 30 and after region 55, but the signals with uniform correlograms were different before region 30 and after region 55. Second, toward the end of the recording as cells began dying (analysis region 142, blue boxes in Supplementary Figure S6A-C), another surge in the number of uniform correlograms occurred.

As shown by the peak count violins in Supplementary Figure S6D, correlogram peak count increased immediately after CO_2_ shutoff (analysis regions 7–46, magenta box in Supplementary Figure S6D and E), declined slowly from analysis region 47–162, then declined rapidly from region 162 to the end of the recording as cells died (blue box in Supplementary Figure S6D and E).

As shown in Supplementary Figure S6E, most correlogram peaks were located between −0.1 s and 0.1 s before CO_2_ shutoff (region 6, red dashed lines in Supplementary Figure S6), with a small subset of peaks located outside this range. In regions 7–81, most peaks were located between −0.2 s and 0.2 s, and more peaks fell outside this range than before CO_2_ shutoff, demonstrating correlogram broadening. Correlogram peak times became more evenly distributed through the correlogram range ($\pm$ 1 s) throughout regions 82–162, with slightly more peaks located between −0.05 s and 0.05 s. Correlogram peak times began to fluctuate in region 162 (blue box in Supplementary Figure S6E), after rapid cell death began (region 142, blue box Supplementary Figure S6A). In region 148, correlogram peaks were equally distributed throughout the correlogram range ($\pm$ 1 s). Peaks in region 149–162 were distributed similarly to region 81–147. Toward the end of the recording (region 164–175), correlograms spread, and most peaks were located between −0.5 and 0.5 s. Additionally, more correlogram peaks were located toward the extremes of the correlogram range compared to the previous recording regions.

Correlogram $A$ (Supplementary Figure S6F) ranged between 0.3–0.7 before the CO_2_ supply was deactivated. Immediately after CO_2_ shutoff (region 6–8), $A$ remained unchanged compared to pre-shutoff values. However, as cells started responding to the lack of CO_2_ buffering (analysis region 12–175, magenta box in Supplementary Figure S6F), correlograms exhibited a wider range in $A$. Finally, as cells began dying (region 164–175), $A$ often assumed an extreme value that randomly fluctuated between 1 and 0 (blue boxes in Supplementary Figure S6F).

#### Impact Injury

Violins of the impact recording are shown in Supplementary Figure S7. After impact, a slight broadening in the peak time distributions (Supplementary Figure S7E) occurred. Before impact, peak times occurring within $\pm$ 0.2 s were mostly distributed closer to 0. After impact, peak times in the $\pm$ 0.2 s range spread more, meaning that correlogram peaks occurred at longer times apart from one another (for example, regions 30, and 46-48). Distributions in the other correlogram metrics did not change dramatically before vs. after impact. Additionally, uniformity and $A$ distributions resemble those from the final analysis region of the culture administered bicuculline (Fig. 7D).

### Summarizing Distributions by Classifying Correlogram Metrics

After plotting the distributions in correlogram metrics (Fig. 7, Supplementary Figures S6 and S7), the algorithm also classifies each metric in order to enhance the ease with which changes in correlogram metric distributions are visualized. As detailed in Sect. "Correlogram Shape Quantification", correlograms were classified as uniform or nonuniform, peak counts were classified as 0, 1, 2, …, 8, 9, or 10 or more peaks, and $A$ was classified into five categories ranging from weak leader/follower nature (simultaneous or inconsistent firing order) to strong leader/follower nature (where one cell in the correlogram lead or followed the other at least 90% of the time). The fraction of correlograms with each classification is shown in Fig. 8 and Supplementary Figures S8 and S9 .

The fraction of correlograms classified as uniform decreased as a result of bicuculline administration (Fig. 8A). Similarly, bicuculline decreased the fraction of correlograms with a high peak count (Fig. 8B). Bicuculline initially decreased the fraction of correlograms classified as strong leader/follower pairs (Fig. 8C). In the long term after bicuculline, the fraction of strong and fairly strong leader/follower pairs doubled compared to before bicuculline, and the fraction of weak and fairly weak pairs decreased.

The fraction of uniform correlograms surged twice during the pH shock recording, once as neuronal interactions altered due to the steadily increasing pH (first surge, region 31-49Supplementary Figure S8A) and once when cells began to die (second surge, region 157-175 Supplementary Figure S8A). Cell death (pH shock) reduced the fraction of correlograms with a high peak count (Supplementary Figure S8B). Additionally, pH shock decreased the fraction of weak leader/follower pairs from ~ 1.0 before CO_2_ shutoff to ~ 0.8 after CO_2_ shutoff (Supplementary Figure S8C). This change occurred simultaneously with an increase in the fraction of fairly weak and intermediate leader/follower pairs from 0 to ~ 0.2 and 0.05, respectively.

The fraction of uniform correlograms remained constant at ~ 0 through the impact recording under bicuculline (Supplementary Figure S9A). Impact slightly reduced the fraction of correlograms with only one peak (Supplementary Figure S9B). The fraction of correlograms with each leader/follower classification remained fairly constant after impact, with only minor and temporary changes (Supplementary Figure S9C). The fraction of fairly strong leader/follower pairs temporarily increased from roughly 0.25 to roughly 0.30 before and after impact (regions 30-37), respectively. The fraction of strong leader/follower pairs temporarily decreased from roughly 0.15 to roughly 0.10 before and after impact, respectively. However, these changes lasted only for regions 30–44 after impact (administered between region 29 and 30). Both fractions returned to pre-impact levels by region 45 (Supplementary Figure S9C). Therefore, while these changes were too slight to easily distinguish by eye in the violin plots (Supplementary Figure S7), there were slight changes in network dynamics as a result of impact. Together, the classification plots in Fig. 8 and Supplementary Figures S8 and S9 reflect the observations in the previous Sects. (“Outputs Independent of Crosscorrelations”–“Violin Plots to Visualize Correlogram Metric Distributions”), with greater ease of observing population level changes in correlogram metrics.

### Tracking Changes in Correlogram Classification

Once correlogram metrics are classified, the algorithm also tracks classifications through time in order to determine whether correlograms with a particular classification are more likely to change than others. The user determines whether to perform this tracking region by region, or grouped before and after each experimental treatment (Table 1). To track correlogram classification, the algorithm compares each correlogram’s classification in a given analysis region, r, to that assigned in the previous analysis region, r-1. Histograms are then constructed to represent the number of times correlograms had a given classification in r, based on the classification in r-1. These histograms are normalized to obtain conditional probability distributions which represent the probability of each correlogram classification in r given the classification in r-1.

#### Bicuculline

Figure 9 shows how correlogram classifications evolved before and after bicuculline administration. The same tracking, but grouped by region, is shown in Supplementary Figure S10. As shown in Fig. 9A, nonuniform correlograms were likely to stay nonuniform both before and after bicuculline administration. Before bicuculline, uniform correlograms stayed classified as uniform ~ 65% of the time. After bicuculline, uniform correlograms stayed uniform only ~ 30% of the time, showing that the observed decreases in uniformity were solely due to uniform correlograms becoming nonuniform. Nonuniform correlograms remained nonuniform with a 95% probability or higher. For correlograms with four peaks or less, bicuculline increased the likelihood that a correlogram exhibited the same or a slightly decreased peak count (plus/minus one or two peaks) between analysis regions (Fig. 9B). Peak count transitioning for correlograms with five to eight peaks was not dramatically altered by bicuculline. Bicuculline reduced the probability that correlograms with 10 peaks or more exhibited the same peak count in the next region from ~ 100% to ~ 80%. Therefore, bicuculline exerted the greatest effect on correlograms with many or few peaks, but not intermediate peak counts. Bicuculline did not dramatically alter the leader/follower transition dynamics (Fig. 9C). This observation supports the notion (from Sect. "Correlogram Creation, Uniformity, Peak Count, and Area Left of Zero"-"Violin Plots to Visualize Correlogram Metric Distributions") that, while bicuculline standardizes cell firing, the drug does not dramatically alter firing order.

The same plots separated by analysis region instead of before vs. after bicuculline (Supplementary Figure S10) show similar trends, but capture classification changes with finer time resolution, and therefore provide more information about short term changes in cell independence, peak count, and firing order. For example, uniform correlograms altered classification the most throughout each analysis region while nonuniform correlograms remained nonuniform with a 90% probability or higher (Supplementary Figure S10A). In the initial response to bicuculline (analysis region 9–11), peak counts were more likely to jump to lower values, which is confirmed by the notion that cells fired in a single burst (Supplementary Figure S10B). In the long term after bicuculline (region 12–14), correlograms were more likely to keep the same peak count, and all correlograms had at least one peak (Supplementary Figure S10B). Similarly, in the initial response to bicuculline (region 9–11), strong leader/followers were more likely to become fairly strong, fairly strong to become intermediate, intermediate to jump between fairly weak and fairly strong, fairly weak to switch randomly, and weak to remain weak (Supplementary Figure S10C). Therefore, in the initial response to bicuculline, correlograms with fairly weak and intermediate dynamics underwent the least predictable firing order changes. In the long term (region 12–14), classifications were more likely to stay the same between regions (Supplementary Figure S10C).

#### Alkalosis

Supplementary Figure S11 shows how correlogram classifications evolved in the pH shock recording. CO_2_ shutoff did not affect transition dynamics between uniform and nonuniform correlograms (Supplementary Figure S11A). Before CO_2_ shutoff, correlograms with fewer peaks were likely to retain the same peak count or change only slightly (Supplementary Figure S11B). Correlograms with higher peak counts had more variation in peak count transitioning, but overall if peak count was higher, the count tended to stay high. After CO_2_ shutoff, peak count transition distributions broadened and became more uniform, meaning the current peak count was less predictable from knowledge of the previous peak count (Supplementary Figure S11B). Before CO_2_ shutoff, correlograms were likely to retain the same leader/follower strength classification, or become one classification group weaker (Supplementary Figure S11C). After CO_2_ shutoff, intermediate correlograms were slightly more likely to transition to stronger and weaker leader/follower classifications (Supplementary Figure S11C). Stronger classifications arose after pH shock, and were more likely to transition to weaker classifications or alter classification randomly.

#### Impact Injury

Supplementary Figure S12 shows how correlogram classifications evolved in the impact recording with bicuculline. Most transition probabilities between correlogram classifications did not change as a result of impact. Only correlograms classified as uniform changed transition probabilities after impact (Supplementary Figure S12A). Before impact, uniform correlograms stayed uniform ~ 38% of the time. After impact, uniform correlograms stayed uniform 33% of the time. This observation suggests that previously independent firing was less likely to stay independent as a result of impact. However, combined with the results in Sect. "Correlogram Creation, Uniformity, Peak Count, and Area Left of Zero"-"Summarizing Distributions by Classifying Correlogram Metrics", this change was not significant enough to affect population dynamics after impact. Correlogram classification evolution changed more in the impact recording without bicuculline (Supplementary Figure S5), as detailed in Supplementary Section S1.

### Correlogram Classification Heat Maps

The algorithm also combines classifications of correlogram uniformity, peak count, and area left of zero to observe how overall correlogram shape evolves. For this article, there are 110 different combinations of the three classifications: two uniformity classifications x five leader/follower strength classifications × 11 peak count classifications. Note that the number 11 depends on the user-specified maximum peak count (Table 1, Sect. "Quantifying and Classifying Firing Pattern") and therefore varies depending on user parameters. To visualize how many correlograms are classified in each of the 110 different combinations, heatmaps showing the percent of correlograms with each classification combination are also generated by the code. Figure 10 shows these heatmaps plotted by periods relative to treatments. The user can also plot these heatmaps for each analysis region if desired.

#### Bicuculline

As shown in Fig. 10A, bicuculline affected correlogram classification groupings. Before bicuculline, 74% of all correlograms were nonuniform with 10 or more peaks. Of this subset, 20.2%, 19.3%, 15.3%, 9.8%, and 9.7% had weak, fairly week, intermediate, fairly strong, and strong leader/follower dynamics, respectively. After bicuculline, these percentages decreased to: 8.0%, 7.9%, 9.0%, 8.3%, and 5.8%. Additionally, the percent of correlograms that were nonuniform, contained only one peak, and exhibited weak, fairly weak, or intermediate leader/follower dynamics increased from, respectively, 0.5%, 0.4%, and 0.3% before bicuculline to 10.1%, 7.8%, and 4.5% after bicuculline. In general, the number of correlogram groupings with fewer peaks was higher after bicuculline. Together, these observations suggest that changes in peak count (firing pattern) dominate correlogram shape changes after bicuculline.

#### Alkalosis

As shown in Fig. 10B, pH shock did not dramatically affect correlogram groupings. However, pH shock did increase the number of possible classification combinations (number of occupied points in the heat map) from 24 to 89. In particular, ~ 82% of all correlograms in the culture exhibited 10 or more peaks and weak leader/follower dynamics before pH shock. Of this subset, 54.9% of correlograms were nonuniform and 27.2% were uniform. After pH shock, the percent of correlograms in each of these groups, respectively, increased to 55.1% and dropped to 15.3%, accounting for ~ 70% of all correlograms in the culture.

#### Impact Injury

As shown in Fig. 10C, impact injury did not affect correlogram classification groupings in the culture with bicuculline. Changes in correlogram classification groups in an impacted culture without bicuculline are discussed in Supplementary Figure S4.

## Discussion

This discussion outlines a variety of considerations for the algorithm, from links to biological mechanisms through algorithm applications and limitations. As the goal of the algorithm is to generate metrics useful in interpreting network dynamics, the success of the algorithm is linked with possible biological interpretations. Therefore, biological interpretations of the metrics are discussed in Sect. "Metric Interpretations for Bicuculline"-"Metric Interpretations for Impact Injury". Literature findings are also compared to algorithm results for each recording to ensure good agreement with the literature. Particularly important for algorithm validation, outputs from the impact injury agreed with previous, manual, literature analysis of the same recording (Sect. "Metric Interpretations for Impact Injury"). The discussion in Sects. "Metric Interpretations for Bicuculline"-"Metric Interpretations for Impact Injury" is then summarized in terms of algorithm validation in Sect. "Algorithm Validation". Based on the biological interpretations and algorithm validation, applications of algorithm outputs are detailed in Sect. "Algorithm Applications". Algorithm limitations are then assessed based on computational complexity (Sect. "Computational Complexity"), statistical approach (described in methods Sect. "Statistics", discussed in Sect. "Limited Statistical Evaluation"), questions arising from biological interpretations of the metrics (Sect. "Limited Mechanistic Insight if not in Conjunction with Additional Measurements"), and finally, comparison to previous platforms for correlogram quantification (Sect. "Complimentary Limitations to Standard Approaches (Functional Connectivity)").

### Metric Interpretations for Bicuculline

#### Heatmaps

As shown by the dark green arrows in Fig. 6B, most of the correlograms in which s21a, s21b, s37b, and s55a participated were uniform before bicuculline, nonuniform after. Therefore, bicuculline made these signals more dependent on others. Conversely, correlograms in which s5a participated (light green arrow, Fig. 6B) were nonuniform before bicuculline, uniform after. This observation suggests bicuculline rendered s5a independent of most of the other signals in the culture. These changes are likely due to the nature of inhibitory GABA_A_ inputs to each cell.

The loss of independence in s21a, s21b, and s37b may indicate that these cells provided excitatory signals inhibited by GABA signaling before bicuculline. For example, signals located on electrode 6, 7, 8, 16, 17, 18, 30, 36, 37, 38, and 39 were not independent of s21a, s21b, and s37b, prior to bicuculline treatment. Signals from these electrodes tended to fire with or slightly before the independent signals (Fig. 6D, dark green arrows). Therefore, cells on electrode 6, 7, 8, 16, 17, 18, 30, 36, 37, 38, or 39 could have released inhibitory neurotransmitters that dampened excitation of the rest of the network by s21a, s21b, and s37b. Such dampening would have rendered s21a, s21b, and s37b independent of the rest of the network. Once GABA_A_ inhibition was removed via bicuculline (Curtis et al., 1971; Johnston, 2013; Stanford et al., 1995), inhibition by cells on these electrodes may have been removed, allowing s21a, s21b, and s37b to be excited by or fire along with other signals from the rest of the culture.

A similar loss of independence occurred for s55a. Unlike s21a, s21b, and s37b, s55a fired before most other signals in the culture both before and after bicuculline (Fig. 6D, lowest dark green arrow). Therefore, s55a may have been independent of other signals before bicuculline as the result of signals from s55a being dampened by inhibitory signaling. When such inhibition was removed by bicuculline, signals from s55a would have been able to excite others, explaining the loss of s55a independence after bicuculline. While the hypothesized mechanism is the same, s55a differs from s21a, s21b, and s37b because s55a firing proceeds firing of the rest of the network while the others fire with the rest of the network. This observation suggests that an excitatory circuit in the culture may be initiated by s55a. Similarly, s21a, s21b, and s37b in concert with cells on electrode 6, 7, 8, 16, 17, 18, 30, 36, 37, 38, and 39 may constitute part of the excitatory circuit downstream of s55a or part of different, parallel excitatory circuits. Such excitatory circuitry may have been unable to excite the network due to GABA_A_ inhibition before bicuculline, thereby rendering the circuit independent of other signals from the network.

Unlike s21a, s21b, s37b, and s55a, s5a gained independence after bicuculline treatment. This gain of independence by s5a after bicuculline can also be explained by changes in inhibitory circuits. The fact that s5a firing was dependent on other signals and tended to fire with or after other signals (Fig. 6D, light green arrow) before bicuculline suggests the cell may have participated in the middle of a firing chain. After bicuculline, s5a became independent and fired before other signals. This observation suggests that the majority of s5a’s activity before bicuculline was dampened by GABA_A_ signaling from cells that fired before s5a. Removing GABA_A_ inhibition of s5a with bicuculline would have allowed the cell to fire without interference, thereby rendering s5a independent of previous GABA_A_ inputs.

While much of the firing order in the culture remained unchanged (unchanged pixel colors in Fig. 6D), a subset of cells assumed different leader/follower dynamics as a result of GABA_A_ inhibition by bicuculline (blue and red arrows in Fig. 6D). For example, only after bicuculline treatment did s18a and s29a consistently fire before most other cells in the culture (Fig. 6D, red arrows). These cells may have been prevented from firing by GABAergic circuits, and loss of inhibition rendered the cells much more likely to fire before others. Conversely, s3a, s39a, and s39b (Fig. 6D, blue arrows) shifted from leading most other signals to following other signals after bicuculline treatment. This shift is potentially due to the increased signal firing (Fig. 4A) after the drug was administered. Increased firing could have overridden or stimulated circuits previously lead by s3a, s39a, and s39b.

Together, all the cells listed in this section likely took part in GABAergic signaling within the culture, either downstream of inhibitory signaling or in possession of inhibitory synapses. Further experiments, such as imaging, are needed to confirm the hypothesized cell interactions that explain the observed changes in correlogram metrics. However, these results show that correlogram metric heatmaps complement one another and can be used to identify electrodes or signals of interest to target such experimentation. Furthermore, heatmaps can be used to identify specific cells to manipulate during an experiment. For example, stimulating a cell that is independent of most others in the network would influence network dynamics less than stimulating a cell that is not independent. Similarly, targeted stimulation or inhibition of a signal that consistently fires before most others could help facilitate experiments on network dynamics.

#### Violin Plots (Metric Distributions)

Distributions of each correlogram metric for the bicuculline recording are shown by the violin plots in Fig. 7. The shape of the violin reflects the distribution of all heatmap (Fig. 6) values for each metric (Hintze & Nelson, 1998; Tanious & Manolov, 2022; Weissgerber et al., 2019). Bicuculline reduced the number of uniform correlograms (uniformity classification and *p*-value distributions, Fig. 7A). This observation is likely due to the fact that bicuculline, like a seizure (De Curtis & Avoli, 2016), eventually synchronizes firing within a culture (G. Gross, 1995). Synchronous activity requires cell coordination, and is therefore incompatible with independent firing. The fact that there are fewer uniform correlograms after bicuculline therefore captures synchronization of the culture due to the drug.

After bicuculline, the number of correlogram peaks decreased and occurred closer together in time (Fig. 7B). These changes suggest that firing patterns between two cells were less diverse after bicuculline was administered. This observation coincides with previous findings that a dose of bicuculline comparable to that administered during the recording (35 μM in the literature, 40 μM in this study) reduces variation in the firing pattern of a culture (G. W. Gross et al., 1997). The smaller correlogram peak times after bicuculline are also expected because bicuculline decreases inhibitory activity in a culture and thereby would allow cells to fire sooner in response to stimulus (Curtis et al., 1971; Johnston, 2013; Stanford et al., 1995). Additionally, the observed decrease in peak timings with $\left|t\right|$ > 0.4 s suggests that firing patterns at longer timeframes were lost. This observation raises the possibility that bicuculline reduces variation in the firing patterns of a culture (G. Gross, 1995; G. W. Gross et al., 1997) by eliminating or altering coordinated activity that occurs over longer time scales.

Before bicuculline, correlograms exhibited relatively few pairs of $i$ and $j$ where one cell consistently lead or followed the other (Fig. 7C). This subset of correlograms decreased immediately after bicuculline administration (analysis regions 9–11), but increased once the culture stabilized into rhythmic activity (Fig. 7C). Together, these observations suggest that the increase in firing shortly after bicuculline administration disrupts leader/follower dynamics, likely because the increase in firing is global, and not dictated by particular circuits. In the long term, once a new steady state of synchronous, rhythmic activity is obtained, more consistent leader/follower pairs emerge as a result of the coordinated activity. As most cell pairs (besides those indicated with arrows in Fig. 6D) exhibited the same leader/follower dynamics before and after bicuculline, these results show that firing order was more consistent, but overall leader/follower relationships unaltered for the majority of signals. Therefore, synchronous activity due to bicuculline is likely not characterized by cells randomly firing together, but instead by all or most cells firing along a consistent circuit at close times to one another. In other words, loss of inhibitory circuits does not necessarily change firing order, but instead allows more coordinated signaling along firing pathways already in place. This hypothesis is confirmed by literature studies which show that bicuculline does not affect synapses in the short term (Brown et al., 2016).

Note that the violin plots inevitably arrive at similar conclusions to those drawn from the heatmaps because heatmap values each contribute to the distribution shown by violin width. However, the violin plots allow better visualization of how the entire population, not individual cells, responds to a treatment. Trends in heatmaps can only be qualitatively compared between different recordings because the individual signals participating in the correlograms differ. However, overall trends in the population are not subject to this limitation and can be quantitatively compared. Therefore, violin shapes and trends can be compared between different recordings and possibly recordings of the same treatment measured by different techniques.

#### Correlogram Metric Classifications and Classification Changes

Plots of the fraction of each correlogram with a given classification (Fig. 8 and Supplementary Figures S8 and S9) reiterate the trends observed from the violin plots, but provide an alternative visualization of violin width. While this visualization was slightly redundant for the bicuculline recording, more subtle changes in violin shape are easier to visualize with classification plots like that in Fig. 8. For example, the impact injury recording showed subtle heatmap changes (results Sect. "Correlogram Creation, Uniformity, Peak Count, and Area Left of Zero", Supplementary Movies S4-S6) that were difficult to observe from violin shape (Supplementary Figure S7), but easier to visualize with classification plots (Supplementary Figure S9B and C). Additionally, correlogram classification allows changes in classification, and by proxy violin shape, to be tracked through time.

Examining how correlogram classifications change can determine whether only certain types of relations, or the population as a whole was affected by experimental treatment. After bicuculline, uniform correlograms stayed uniform only ~ 30% of the time while nonuniform correlograms remained nonuniform with a 95% probability or higher (Fig. 9A). This observation supports the notion that cells previously independent of the signaling network (uniform correlograms) joined highly synchronous network firing after the loss of GABA_A_ inhibition by bicuculline treatment. Bicuculline increased the likelihood that a correlogram exhibited the same peak count (plus/minus one or two peaks) between analysis regions (Fig. 9B). The fact that peak count after bicuculline was less likely to change provides further evidence that culture firing is less varied after bicuculline. This observation agrees with literature showing that bicuculline standardizes cell firing patterns and minimizes spiking variations in the network (G. W. Gross et al., 1997). Bicuculline did not dramatically alter the leader/follower transition dynamics of correlograms at the population level (Fig. 9C). This observation supports the notion (discussed in Sect. "Correlogram Creation, Uniformity, Peak Count, and Area Left of Zero"-"Violin Plots to Visualize Correlogram Metric Distributions") that the drug alters firing order for only a few cells. Furthermore, from the transition plots, it is clear that correlogram metrics remain consistent due to correlograms maintaining current classifications, rather than exchanging classifications with others. In other words, instead of altering only a subset of firing relations (correlograms with a specific classification), bicuculline renders all relations more consistent in firing pattern and order. The transition probability plots help distinguish between these two cases.

#### Combining Correlogram Metric Quantifications

While the heatmaps in Fig. 10A provide an alternate visualization and thereby reinforce conclusions drawn from previous bicuculline recording plots, the heatmaps provide easy identification of which correlogram metric contributed most to correlogram shape alterations. After bicuculline, changes in peak count (firing pattern) dominate correlogram shape changes. This observation suggests that firing pattern changes dominate cellular responses to bicuculline. Furthermore, this effect is expected based on literature observations that firing patterns are standardized (G. Gross, 1995; G. W. Gross et al., 1997), but that synapses are not lost (Brown et al., 2016) after bicuculline treatment.

### Metric Interpretations for pH Shock

#### Violin Plots (Metric Distributions)

No specific correlograms stood out in the pH shock recording heatmaps (Supplementary Movies S1-S3). However, violin plots for the pH shock recording (Supplementary Figure S6) showed that, while no individual correlogram stood out, most correlogram shapes were altered under pH shock. Correlograms experienced two surges in uniformity. The first surge occurred after the CO_2_ supply was deactivated (analysis region 6) when the pH began increasing (lasting from analysis region 30–55, magenta box, Supplementary Figure S6B and C). The second surge occurred at the end of the recording (region 142 to the end) as cells died (blue box, Supplementary Figure S6B and C). The first surge in uniformity suggests that cell interactions changed as a result of pH shock, potentially due to alterations in synapses, GABAergic firing, or ion distributions influenced by pH (Sinning & Hübner, 2013). The second surge in uniformity likely arose from decreased spike counts, and hence lack of coordinated firing, as cells died.

As shown by the peak count violins in Supplementary Figure S6D, correlogram peak count increased immediately after CO_2_ shutoff (analysis regions 7–47, magenta box, Supplementary Figure S6D and E), declined slowly from analysis region 47–162, then declined rapidly from region 162 to the end of the recording as cells died (blue box, Supplementary Figure S6D and E). The initial increase in peak count as CO_2_ left the system, but before the pH was dramatically affected suggests that firing patterns were initially more diverse. These observations agree with literature observations that a slight pH change can, for non-GABAergic cells, increase culture spiking (Church & McLennan, 1989; Jarolimek et al., 1990; Lu et al., 2012; Sinning & Hübner, 2013) and cause seizures in patients (Aram & Lodge, 1987; Helmy et al., 2012). However, these findings seem to contradict the observed decrease in mean firing rate discussed in Sect. "Outputs Independent of Crosscorrelations" (Fig. 4B) and literature showing decreased firing in alkaline conditions for GABAergic cells (Jarolimek et al., 1990; Sinning & Hübner, 2013; Sun et al., 2012; Zhang et al., 2013). It is important to note that pH response is cell dependent (Sinning & Hübner, 2013), and not all cells follow the mean trend, as shown by the magenta arrows in Fig. 4B and the heatmaps in Supplementary Movie S2. Therefore, while mean spikes per minute decrease, firing pattern in response to pH is cell-dependent and more varied. The decline in peak count starting at region 47 coincides with the end of changes in correlogram uniformity (Supplementary Figure S6B and C). This observation suggests that after network communication (signal (in)dependance) altered in response to the increasing pH, firing patterns were also less diverse. For the rest of the recording, as the pH continued increasing, firing pattern diversity steadily decreased, even though spike counts gradually increased or remained constant (Fig. 4B and Supplementary Figure S6A and D). These results suggest that the initial increase in firing pattern diversity occurred before alkalosis progressed, and that alkalosis simplifies firing patterns. This finding is consistent with previous observations that firing patterns are less diverse and firing count decreases in GABAergic neurons during alkalosis (Jarolimek et al., 1990; Sinning & Hübner, 2013; Sun et al., 2012; Zhang et al., 2013). Since these cortical cultures are at least in part GABAergic (or the cultures would not be sensitive to bicuculline), it is likely that GABA signaling is present. Therefore, heterogeneous cell responses to alkalosis and eventual firing decrease agree between algorithm outputs and the literature. The final decrease in peak count starting in region 142 was the result of the decreasing number of spikes in each correlogram due to dying and dead cells.

Initially after CO_2_ shutoff (region 7–81), correlogram peak times expanded away from $t$ = 0. This slight broadening of correlogram peak time distributions (starting in the magenta box, Supplementary Figure S6E) corresponds with the longer interspike intervals observed after CO_2_ shutoff (see Sect. "Outputs Independent of Crosscorrelations"). Correlogram peak times continued to broaden away from $t$ = 0 throughout regions 81–147, indicated by the fact that violin shape became more uniform (the denser bulge between $\pm$ 0.1 s decreased in width, and the violin looked more rectangular). Therefore, as alkalosis progressed, cells were more likely to fire at randomly longer intervals, in agreement with fluctuating firing patterns as a result of pH changes (Supplementary Figure S6D). Correlogram peak time distribution shapes fluctuated after rapid cell death began (region 142), until the end of the recording. These fluctuations suggest that firing patterns and timings fluctuate as cells die, which is not surprising given the interplay between neuron firing and cell death pathways (Hara & Snyder, 2007).

Correlogram $A$ (Supplementary Figure S6F) ranged between 0.3–0.7 before the CO_2_ supply was deactivated. As cells started responding to the lack of CO_2_ buffering (analysis region 12–175, magenta box, Supplementary Figure S6F), correlograms exhibited a wider range in $A$. This observation suggests that firing order became slightly more consistent after CO_2_ shutoff. Combined with the coincident decrease in firing pattern diversity (Supplementary Figure S6D), this observation further supports the notion that network communication altered in response to pH stress. Finally, as cells began dying (region 164–175), $A$ often assumed a value of 1 or 0. This is due to decreasing spike counts resulting in increasingly sparse correlograms as cells died. With fewer spikes, all components of the correlogram are more likely to fall on one side of $t=0$, meaning $A$ becomes 0 and 1, or randomly fluctuates between the two. Therefore, fluctuations in $A$ at the end of the pH shock recording are artifacts due to sparse correlograms arising from cell death, rather than shifts in neuronal firing order.

Together, the discussion in this section suggests that algorithm metrics relate to heterogeneous alkalosis effects observed in the literature (Aram & Lodge, 1987; Church & McLennan, 1989; Helmy et al., 2012; Jarolimek et al., 1990; Lu et al., 2012; Sinning & Hübner, 2013; Sun et al., 2012; Zhang et al., 2013). Therefore, algorithm metrics do not conceal or average out effects of heterogeneity in individual signals, even when considering the network as a whole. This observation raises the possibility of using correlogram uniformity, peak count, and area left of zero to understand how biological heterogeneity affects overall response to pathological pH states and a variety of other conditions.

#### Correlogram Metric Classifications and Classification Changes

Classification plots (Supplementary Figure S8) reflect the observations made for the pH shock violin plots. However, metric classification allowed for additional information on how fluctuations in cell activity during alkalosis arose. CO_2_ shutoff did not affect transition dynamics between uniform and nonuniform correlograms (Figure S11A). Therefore, changes in correlogram uniformity likely arose from varied alterations to individual signal firing, rather than a specific effect on a particular class of cell interactions (for example, nonuniform). After CO_2_ shutoff, peak count transition distributions became more uniform, meaning the current peak count was less predictable from knowledge of the previous peak count (Figure S11B). This change likely reflects the fact that firing patterns continuously altered with changing pH, as detailed in the violin plot discussion.

Correlograms with weak or fairly weak leader/follower classifications tended to stay the same after CO_2_ shutoff. Therefore, if little consistent firing order was observed between two signals, alkalosis was unlikely to impose firing order. Correlograms with intermediate leader/follower classification were most affected by alkalosis. Correlograms classified as exhibiting intermediate leader/follower consistency were more likely to transition to weaker leader/follower classifications (Supplementary Figure S11C). While less frequent than a transition to weaker leader/follower classifications, intermediate correlograms were also more likely to transition to stronger leader/follower classifications after CO_2_ shutoff (less than 20% probability of transitioning to a stronger classification, compared to 60% probability of transitioning to a weaker classification, Supplementary Figure S11C). Stronger classifications arose only after pH shock and altered classification randomly. As discussed with the violin plots, the rapid gain and loss of strong leader/follower pairs likely arose due to a combination of continuously changing firing patterns and sparse correlograms as cells died. Together, the observations from Supplementary Figure S11C suggest that alkalosis exerted the biggest effect on signal pairs which exhibited a consistent (where one signal leads 70–80% of the time), but not fixed, firing order. Targeting such signal pairs for additional experimentation, such as imaging for synapses, would likely help elucidate changes and characteristics of cells most responsive to alkalosis.

#### Combining Correlogram Metric Quantifications

As shown in Fig. 10B, pH shock did not dramatically affect correlogram groupings, but did increase the number of classification combinations (number of occupied points in the heat map) into which correlograms were classified from 24 to 89. This increase was mostly due to an increase in the number of leader/follower classifications after pH shock. Therefore, while changes in correlogram shape were not dramatic, leader/follower classification fluctuations expanded the number of states in which correlograms existed. This observation once again reinforces the notion of fluctuating cell dynamics due to heterogeneous responses to pH shock.

### Metric Interpretations for Impact Injury

Manual correlogram analysis has already been performed for the impact recording with bicuculline used in this article (Rogers & Gross, 2019). This previous analysis suggested that impact caused more random spiking between pairs of cells (Rogers & Gross, 2019). However this random spiking resulted in decreased correlogram peak prominence, and did not render correlograms uniform (Rogers & Gross, 2019). Additionally, the previous literature analysis found that correlograms (1 ms bins, ranging from −0.1 s to 0.1 s) broadened slightly after impact compared to before and that correlogram peaks shifted slightly (Rogers & Gross, 2019). However, neither change dramatically altered peak count or area left of zero for most of the correlograms in the culture (Rogers & Gross, 2019). Together, these literature findings suggest that correlogram shape metrics calculated in this article should not change dramatically after impact (Rogers & Gross, 2019). The impact recording therefore acts as a negative control for algorithm outputs, since correlogram uniformity, peak count, and area left of zero should be subtly, not dramatically altered. The fact that, as expected, algorithm outputs for correlogram uniformity, peak count, and area left of zero were not dramatically altered after impact (Sects. "Correlogram Creation, Uniformity, Peak Count, and Area Left of Zero"-"Correlogram Classification Heat Maps") demonstrates that the algorithm does not falsely detect changes if no changes are present. Combined with the observed changes in the bicuculline and pH shock recording (Sect. "Results"), these observations suggest that algorithm metrics serve to identify altered cell dynamics, and will not falsely detect changes if none are present. Therefore, algorithm metrics are a useful tool to automate correlogram shape analysis.

The heat map movies of the impact recording (Supplementary Movies S4-S6), showed that each metric varies slightly after impact, but does not change dramatically (the shading in each pixel is slightly different). These findings agree with the subtle, but not dramatic changes expected from literature measurements (Rogers & Gross, 2019). This lack of dramatic change in correlogram metrics is also supported by the violin plots in Supplementary Figure S7, which confirm that correlogram shape metrics are not dramatically different across the entire network.

Subtle changes in correlogram metric distributions for the impact recording are easier to visualize with classification plots like that in Fig. 8. While difficult to observe in the violin plots, the fraction of correlograms with each leader/follower classification underwent minor and temporary alterations after impact injury. The fraction of fairly strong leader/follower pairs temporarily increased. Conversely, the fraction of strong leader/follower pairs temporarily decreased (Supplementary Figure S9C). These changes lasted only for regions 30–44 after impact (administered between region 29 and 30), and returned to pre-impact levels by region 45 (Supplementary Figure S9C). Therefore, firing order was temporarily less consistent after impact injury. While this change was too slight to easily distinguish by eye in the violin plots (Supplementary Figure S7), the classification plots (Supplementary Figure S9) confirm the notion from the heat maps (Supplementary Movies S4-S6) that subtle alterations in network dynamics occur after impact under bicuculline. Therefore, the classification plots in Supplementary Figure S9 reinforce, but provide more detail on the results observed in the heatmaps (Supplementary movie S4-S6) and violin (Supplementary Figure S7) plots. For this recording, the classification plots enhance the ease of observing subtle population level changes in correlogram metrics. Note that classification plots merely reiterated information from the violins for both bicuculline treatment and pH shock (Sect. "Metric Interpretations for Bicuculline"-"Metric Interpretations for pH Shock"), and did not provide much additional information. Therefore, the impact recording results suggest that classification plots are more helpful when alterations to the correlograms are more subtle.

While correlogram shape was not dramatically altered by impact injury, changes in rhythmic activity after impact were observed from correlograms in the previous study (Rogers & Gross, 2019). Specifically, correlogram peaks arising from rhythmic activity occurred with a lower frequency, meaning further apart in time, after impact (Rogers & Gross, 2019). The broadened peak time distributions observed in this article for the same MEA recording (Sect. "Violin Plots to Visualize Correlogram Metric Distributions", Supplementary Figure S7E) also suggest longer intervals between correlogram peaks after impact, and are therefore consistent with the literature. This result is particularly noteworthy because the automated analysis and literature analysis were performed with different approaches. As detailed in Sect. "Violin Plots to Visualize Correlogram Metric Distributions", the algorithm automatically considered peak times from all possible crosscorrelograms in the culture. The previous literature analysis was performed manually (Rogers & Gross, 2019) because automated peak timing analysis was not easily accessible. To render such manual analysis feasible, a single reference cell was selected according to the parameters detailed in the previous publication (Rogers & Gross, 2019), and correlograms with only this cell as reference were analyzed (Rogers & Gross, 2019). The observed agreement between the manual literature analysis of a subset of the data and the automated analysis of all crosscorrelograms validates both the literature findings and the efficacy of the algorithm described in this article. Different types of analysis arrived at the same conclusion. The literature study has the limitation of manual analysis, but the advantage of directly identifying peaks due to rhythmic activity. The algorithm in this article has the advantage of high throughput, but the detriment that peaks due to rhythmic activity are not directly identified, and only peak times in general are considered. However, methods to detect correlogram peaks arising from rhythmic activity have already been developed (Eggermont, 2014; Ito et al., 2014), and can be employed in future versions of the analysis algorithm.

Algorithm outputs independent of correlograms and correlogram-related outputs can also be compared to provide a better understanding of cell dynamics. For example, for the impact recording, lengthened rhythmic activity intervals after impact were observed by the algorithm (Sect. "Violin Plots to Visualize Correlogram Metric Distributions", Supplementary Figure S7E) and literature (Rogers & Gross, 2019). However, the interspike interval distributions (Sect. "Outputs Independent of Crosscorrelations", Fig. 5C), indicate that the mean rhythmic interspike interval decreased from ~ 2.3 s to ~ 1.7 s as a result of impact. This seeming contradiction likely arises due to different time frames being considered. In the previous literature (Rogers & Gross, 2019) and this study, correlogram metrics only considered spikes that occurred less than one second from one another. However, the interspike interval distribution showed changes in spikes that occurred longer than 1 s apart. Together, these observations suggest that the short-timescale and long-timescale firing responses to impact differ. This observation matches those of EEG studies that demonstrate both increased and decreased EEG signal frequencies as a result of injury (Atlan & Margulies, 2019; Ianof & Anghinah, 2017). Most EEG signal frequencies decrease, meaning longer intervals between signals, after traumatic brain injury (Atlan & Margulies, 2019; Ianof & Anghinah, 2017). These agree with the results based on correlogram peak times. However, an increase in seizure like activity, during which signals occur rapidly and close together, is also observed in post-injury EEG studies (Atlan & Margulies, 2019; Ianof & Anghinah, 2017), agreeing with the observed decrease in the interspike interval histograms. Therefore, the shorter rhythmic activity intervals captured by the interspike interval histogram might relate to seizure like activity in patients, while the longer intervals captured by crosscorrelogram metrics might correspond to general slowing of intervals after injury. Future comparison of analysis algorithm metrics and multiscale modeling might be able to relate the complex dynamics between short term and long term rhythmic activity and bridge the gap between different recording methods. Additionally, the similarities between MEA recordings from neuronal cultures and EEG recordings of entire brains suggest that in vitro platforms to study injury can provide translatable insight into signaling changes after TBI.

### Algorithm Validation

As detailed in Sects. "Metric Interpretations for Bicuculline"-"Metric Interpretations for Impact Injury", the algorithm was able to describe alterations in network dynamics when firing dramatically altered when signals were not lost (bicuculline treatment, Sect. "Metric Interpretations for Bicuculline") and when network dynamics altered and signals were lost in response to stress (pH shock, Sect. "Metric Interpretations for pH Shock"). Furthermore, algorithm metrics do not show false changes when network dynamics remain constant and agree with manual literature analysis (impact injury under bicuculline, Sect. "Metric Interpretations for Impact Injury"). For all validation recordings, algorithm metrics therefore behave as expected. These results indicate that quantifying correlogram uniformity, peak count, and area left of zero provides useful information about network dynamics in a culture, and will not give false positive or false negative results. Additionally, results from the alkalosis recording demonstrate that algorithm metrics do not conceal or average out effects of heterogeneity in individual signal responses, even when considering the entire network. Furthermore, the literature on neuronal responses to pH shock and injury reported conflicting effects, where cells seem to both increase and decrease firing, or where activity occurs at both shorter and longer intervals across multiple length scales. Algorithm metrics also exhibited similar heterogeneity for both the alkalosis and impact recordings. Therefore, more targeted exploration of algorithm metric heterogeneity will likely serve as a useful means of understanding how seemingly conflicting changes arise in different experimental conditions. Algorithm metrics therefore serve as a useful means of employing correlograms to quantify neuronal dynamics from an individual to population level without losing information on network responses or yielding false positive or negative results.

### Algorithm Applications

As detailed throughout the results and discussion, algorithm outputs are able to capture time-dependent changes in cell dynamics under each experimental condition and agree with expected changes based on the scientific literature. Automated quantification of correlogram shape metrics overcomes the limitations of manual qualitative analysis of correlogram shape. By overcoming these limitations, a greater number of correlograms can be rapidly examined over a greater number of experimental time points from an individual to population level. Therefore, the algorithm is a useful tool to rapidly and automatically quantify changes in firing dynamics from individual signals to populations of signals.

For any new recording, the optional function to plot individual correlograms should be used to ensure that the correlograms being analyzed are not sparse. If correlograms are sparse, correlograms with longer analysis region size, different range, or different binning parameters should be used. If correlograms are not sparse, changes in correlogram metrics arise from alterations to relative signal timings.

Correlogram metric heatmaps allow signals or electrodes of interest to be identified, and can therefore help target additional experimentation. Additionally, the optional feature of the algorithm which allows particular signals to be excluded from analysis allows signals of interest to be deleted for targeted in silico investigation of how these signals affect the network as a whole.

Violin plots of correlogram metrics are a useful means of confirming changes visualized in the heat maps and comparing how population trends in correlogram properties change across analysis regions. Additionally, since violin plots show distributions and do not focus on individual signals, it is possible to compare violin shapes across recordings in order to assess how population dynamics vary across biological replicates or different experimental treatments. If violin shapes differ, the data distribution is altered and the two violins are significantly different (Hintze & Nelson, 1998; Tanious & Manolov, 2022).

Metric classification provides alternative visualization of violin plot width. Classification plots reinforce the heatmap and violin observations. However, subtle population level changes in correlogram metrics are easier to visualize in classification plots. Additionally, classifying correlogram metrics allows tracking of how these classifications evolve through time. This tracking can help identify whether certain classes of signal pairs are more susceptible to a given experimental treatment. Furthermore, in the event that correlogram classification remains fairly constant throughout a recording, tracking can distinguish whether these constant values are due to individual correlograms randomly exchanging classification (for example, the pH shock recording) or due to correlograms maintaining the same classification (for example, the bicuculline and impact under bicuculline recordings).

The grouped classification heatmaps help visualize how overall correlogram shape changes for the entire population, and can also help identify which of the three correlogram metrics (independence, firing pattern, or firing order) most affect cell dynamics in the population.

The main advantage of the analysis algorithm in this article is that correlogram metrics are summarized by distributions and based on signal timings without making any assessment of functional connectivity. In this manner, the algorithm bridges the gap between observed changes in individual signal dynamics, *i.e.* each unique correlogram, and overall changes in population dynamics. It is easier to compare population distributions between different recordings (for example, biological replicates in an experiment), and even across different recording techniques.

The metrics in this algorithm use relative signal timings to help identify whether signal independence, pattern, and order are altered by experimental conditions, not the mechanism underlying these changes. These three features play a role in any type of multi-signal recording technique, regardless of length scale, and are therefore easier to compare across large (EEG) and small (MEA) scale recordings. To obtain mechanistic insight into changes in correlogram metrics, algorithm metrics should be compared against data from other experiments. While assessing functional connectivity using correlograms has already been extensively described (Awiszus, 1992; Eggermont, 2014; Gerstein & Perkel, 1972; Hu et al., 2022; Kobayashi et al., 2019; Melssen & Epping, 1987; Moore et al., 1966, 1970; Narayanan & Laubach, 2009; Saberi-Moghadam et al., 2018; Shao & Tsau, 1996; Wang et al., 2024), and even coded in MATLAB (Narayanan & Laubach, 2009), such calculations are not part of the current algorithm in order to focus on metrics that can be calculated with fewer assumptions. The current algorithm outputs make no assumptions about connections or correlations between cells, and instead only consider signal timings. Relative signal timings can be affected by a number of variables beyond functional connectivity. It is therefore ideal to validate mechanistic hypotheses generated from algorithm outputs or perform functional connectivity analysis of correlograms via additional experiments such as direct imaging. Without comparison to other experimental data, this algorithm should only be used to assess how signal independence, pattern, and order are altered due to experimental treatments, and compare whether the observed alterations are consistent through time, between biological replicates, and even across different recording techniques.

A final benefit of the metrics in this article is easier comparison across length scales and recording techniques. Dependence, pattern diversity changes, and consistency in firing order have a similar interpretation regardless of whether the pattern corresponds to individual action potentials or features of coordinated brain activity. Therefore, in the future, these metrics can be compared between different scales (for example, TBI-on-a-Chip or in vivo MEA recordings compared to EEG) to relate how alterations in firing dependence, pattern diversity, and order at cellular scales affect firing dependence, pattern diversity, and order of coordinated activity in larger cell populations and brain regions. While it is also possible to compare functional activity metrics across length scales, interpretation of such comparison is less straightforward (Fedele et al., 2020; Grienberger & Konnerth, 2012; Michel & Brunet, 2019; Obien et al., 2015; Peterka et al., 2011). To link changes in cell interactions to changes in brain region connectivity, it is helpful to determine whether cell scale connectivity changes alter signaling features like firing pattern, and if these alterations in turn relate to the changes in brain region connectivity. Therefore, the metrics in this algorithm can also be helpful in understanding and quantifying the effects of functional connectivity changes. The possibility of bridging different scales and complimenting functional connectivity measurements makes the algorithm a useful tool to quantify changes in neuronal dynamics as a result of different conditions or treatments.

### Computational Complexity

The highest computational demand of the algorithm occurs during correlogram creation. The algorithm must compare ${N}^{2}$ different signals in a single analysis region (containing a total of ${S}_{r}$ raster entries from all signals) to generate correlograms. For each of these ${N}^{2}$ comparisons, there is a total of ${s}_{i}{s}_{j}$ events to compare, where ${s}_{i}$ and ${s}_{j}$ are the number of events in the given analysis region recorded from raster $i$ and $j\in \left[1,N\right]$, respectively. Therefore, a total of $T$ comparisons are made for a single analysis region $r$.

3 $${T}_{r}= {\sum }_{i=1}^{N}{\sum }_{j=1}^{N}{s}_{i}{s}_{j}$$

If the spike counts of $1\le q\le N$ unique signals increase by perturbation $k$, then the new ${S}_{r}$ is the original region spike count (${S}_{r,0}$) plus the number of spikes added (${\Delta S}_{r}$).

4 $${S}_{r}= {S}_{r,0}+{\sum }_{i=1}^{q}{k}_{i}= {S}_{r,0}+{\Delta S}_{r}$$

The change in ${T}_{r}$ (${\Delta T}_{r}$) is given by Eq. 4.

5 $${\Delta T}_{r}={\left({\sum }_{i=1}^{q}{k}_{i}\right)}^{2}+ 2{S}_{r,0}\left({\sum }_{i=1}^{q}{k}_{i}\right)= {\left({\Delta S}_{r}\right)}^{2}+ 2{S}_{r,0}\left({\Delta S}_{r}\right)$$

The dominant term in ${\Delta T}_{r}$ depends on the value of ${S}_{r,0}$ relative to ${\Delta S}_{r}$. If ${S}_{r,0}\ll {\Delta S}_{r}$, the first term of Eq. 5 dominates, and complexity scales on the order of ${\left({\Delta S}_{r}\right)}^{2}$. However, if ${S}_{r,0}\gg {\Delta S}_{r}$, the second term of Eq. 5 dominates, and computational complexity scales on the order of ${\Delta S}_{r}$. Increasing both $N$ and the number of raster events in the given region will therefore scale computational complexity. Note that, if applied to every analysis region, computational complexity will scale by some factor of the total number of analysis regions.

6 $${\Delta T}_{total}={\sum }_{r=1}^{R}{\left({\Delta S}_{r}\right)}^{2}+ 2{S}_{r,0}\left({\Delta S}_{r}\right)$$

From Eq. 6, the number of analysis regions ($R$), the number of signals ($N$), and the number of raster events in each region (${S}_{r}$) all affect computational complexity. However, ${S}_{r}$ will exert the greatest effect. Two steps are employed by the algorithm to slightly reduce ${S}_{r}$ before correlogram creation. First, the algorithm calculates ${t}_{c}- {t}_{r}$ for each reference, then uses a logical to isolate only comparison spikes with $\left|{t}_{c}- {t}_{r}\right|\le$ the correlogram range. This step thereby slightly reduces ${S}_{r}$ before obtaining correlogram bin counts. Additionally, if a signal does not fire in the particular analysis region, the algorithm adjusts $N$ accordingly for that region before performing any correlogram creation. However, these steps exert only minor benefit.

If the algorithm takes too long to generate correlograms or issues a memory error, analysis region duration should be shortened. Conversely, region duration should be increased if the algorithm runs quickly, but there are very few spikes in each region and correlograms are sparse. Decreasing correlogram range (for example, from 1 s to 0.2 s) can also decrease computational complexity because more spikes will be excluded from the correlogram range logical. However, decreasing correlogram range alone will have less impact on ${S}_{r}$ than shortening region duration.

To help the user assess computational complexity, the algorithm displays elapsed time in MATLAB’s command window when the script finishes running. With the seven minute analysis regions in this article, the algorithm finished analyzing the bicuculline recording in under 1.1 min, the alkalosis recording in under 5.1 min, the impact under bicuculline recording in under 20.2 min, and the impact without bicuculline recording in under 3.2 min. As expected, algorithm runtime depends on the number of signals and the spiking of each signal. Even with the same correlogram and region parameters, the algorithm will require different amounts of time to run for different recordings. Note that the pH shock recording, while the longest (20.5 h, 34 units) was processed ~ 4× faster than the impact recording under bicuculline (6 h, 69 units). Therefore, more analysis regions is less expensive than more units or more spikes, as expected from Eq. 6.

Note that the algorithm saves all correlograms and resulting metrics. The script can be run multiple times on the same recording. To save time, subsequent iterations of the script will load the previously generated correlograms and metrics. However, if desired, the user can override this data loading to enforce correlogram generation and analysis from scratch (Table 1).

### Limitations and Future Improvements

The discussion so far has described the benefits of quantifying correlogram uniformity, peak count, and area left of zero in understanding neuronal dynamics. However, there are limitations to this approach that could be improved (Sect. "Limited Statistical Evaluation"), can be compensated for with additional experimentation (Sect. "Limited Mechanistic Insight if not in Conjunction with Additional Measurements"), and that compliment limitations of the standard approaches that estimate functional connectivity from correlograms (Sect. "Complimentary Limitations to Standard Approaches (Functional Connectivity)").

#### Limited Statistical Evaluation

A key limitation of the analysis pipeline is that the shapes of metric violins were qualitatively compared to assess whether metric distributions changed (details in Sect. "Statistics"). For violin shape changes to indicate significant changes in the data distribution, correlograms must not be sparse and a higher number of signals should be recorded (Hintze & Nelson, 1998; Tanious & Manolov, 2022; Weissgerber et al., 2019). More quantitative statistical comparison of the metrics across analysis regions has been left to future work. However, the qualitative approach described in this article works if there are enough signals and correlogram properties are adequately set to ensure the correlograms are not sparse (Hintze & Nelson, 1998; Tanious & Manolov, 2022; Weissgerber et al., 2019).

#### Limited Mechanistic Insight if not in Conjunction with Additional Measurements

Algorithm metrics alone do not provide mechanistic insight. The purpose of this algorithm is not to provide details on signal connections, but to provide more general metrics associated with signal relations that can be compared across recordings. If the objective is to obtain mechanistic insight, additional measurements are required to plot against changes in dependence, firing pattern, or firing order. For example, quantification of synapses from immunofluorescence images can be plotted against metric violins to determine whether altered synaptic connectivity explains alterations in firing relations. Biomarker quantification through immunocytochemistry, immunohistochemistry, qPCR, Western Blotting, ELISA, or similar techniques can be plotted against algorithm metrics to look for mechanistic relations between the biomarker and network dynamics at a population level. Additionally, stimulation or inhibition of specific signals using patch clamp, electrodes, or optogenetics would allow targeted study of how altering specific signals influence the correlogram metrics describing network dynamics. Regardless of these possibilities, algorithm metrics alone should only be used to indicate whether signal relations have changed. The mechanisms underlying such changes should only be considered if algorithm metrics are analyzed against data from other experimental techniques. However, the same can be argued for functional connectivity, and these limitations are therefore not different from those of the standard approaches to quantify correlograms.

#### Complimentary Limitations to Standard Approaches (Functional Connectivity)

The approach to correlogram quantification in this article is based on well-established principles (Bartram et al., 2024; Eggermont, 2014; Gerstein & Perkel, 1972; Ito et al., 2014; Moore et al., 1966, 1970; Perkel et al., 1967; Rogers & Gross, 2019), and determines whether experimental factors alter dependence, interaction pattern, or order of signals in a network. However, this is not the standard approach to correlogram quantification. The standard approach is to estimate functional connectivity from correlograms (Awiszus, 1992; Bartram et al., 2024; Durstewitz, 2017; Eggermont, 2014; Gerstein & Perkel, 1972; Hu et al., 2022; Ito et al., 2014; Kobayashi et al., 2019; Melssen & Epping, 1987; Moore et al., 1966, 1970; Narayanan & Laubach, 2009; Pereda et al., 2005; Perkel et al., 1967; Saberi-Moghadam et al., 2018; Shao & Tsau, 1996; Wang et al., 2024). Compared to the standard approach, the metrics in this article make no assessment of underlying connections and mechanisms. However, by avoiding connectivity assessment, the metrics in this algorithm will not inadvertently mistake common input as cause, a limitation of functional connectivity assessment from correlograms (Awiszus, 1992; Durstewitz, 2017; Eggermont, 2014; Gerstein & Perkel, 1972; Kobayashi et al., 2019; Melssen & Epping, 1987; Moore et al., 1966, 1970; Narayanan & Laubach, 2009; Pereda et al., 2005; Perkel et al., 1967; Saberi-Moghadam et al., 2018; Shao & Tsau, 1996). Furthermore, functional connectivity assessment based on correlograms assess only linear relations between signals (Pereda et al., 2005). Alternative approaches to assess functional connectivity such as transfer entropy rely only on rasters (Ito et al., 2011; Novelli et al., 2019; Timme & Lapish, 2018; Vicente et al., 2011), can identify nonlinear correlations (Novelli et al., 2019; Timme & Lapish, 2018), and often outperform functional connectivity assessments based on cross-correlation (Ito et al., 2011; Vicente et al., 2011). For these reasons, the approach in this article does not employ correlograms to assess connections between signals, resulting in limitations complimentary to functional connectivity approaches. As outlined in this section, future approaches would be more powerful by combining the metrics in this algorithm with functional connectivity metrics.

There are many approaches to assess functional connectivity from correlograms to determine whether one signal causes another (Awiszus, 1992; Bartram et al., 2024; Durstewitz, 2017; Eggermont, 2014; Gerstein & Perkel, 1972; Hu et al., 2022; Ito et al., 2014; Kobayashi et al., 2019; Melssen & Epping, 1987; Moore et al., 1966, 1970; Narayanan & Laubach, 2009; Pereda et al., 2005; Perkel et al., 1967; Saberi-Moghadam et al., 2018; Shao & Tsau, 1996; Wang et al., 2024). Additionally, correlogram-free approaches to assess functional connectivity are on the rise (Ito et al., 2011; Novelli et al., 2019; Timme & Lapish, 2018; Vicente et al., 2011), and outperform correlogram-based approaches (Ito et al., 2011; Vicente et al., 2011). In particular transfer entropy is a correlogram-free approach that arises from information theory, which requires very few assumptions (Novelli et al., 2019; Timme & Lapish, 2018). Furthermore, transfer entropy can be compared across different techniques and different types of data (Timme & Lapish, 2018). As with the metrics in this article, transfer entropy distributions would be helpful to compare different analysis regions or different recordings. Such comparison would determine how strongly signals cause each other throughout a recording, and whether this causal strength is altered by experimental treatments. Therefore, transfer entropy is an ideal functional connectivity assessment to combine with algorithm metrics.

Transfer entropy is subject to the same limitations of functional connectivity analysis in general, in that if two signals are controlled by a different, unmeasured signal, a false causal relation will be identified between the two signals (Timme & Lapish, 2018). Therefore, while not always possible, it is ideal to validate causal relations identified using transfer entropy, or similar functional connectivity metrics, with stimulation or inhibition experiments and imaging. If the user cannot validate causal relationships in the given experimental setup and does not wish to risk mistaking common inputs as causal, the user may prefer an option to quantify network dynamics without making any evaluation of functional connectivity. In such a case, the metrics in this algorithm can be more helpful than functional connectivity assessment. While still indicating altered firing dynamics, there is no possibility for uniformity, peak count, and area left of zero to mistake common input as a causal relationship because these metrics do not assess causality.

Functional connectivity metrics are related, but not identical to the metrics in this article. For example, if one signal causes or inhibits another, the correlogram of the cause vs. effect signal should exhibit higher area left of zero and a higher transfer entropy. Quantifying area left of zero alone answers whether firing order changed, even if there is no causal link between signals. Quantifying transfer entropy or similar functional connectivity metrics alone answers whether causal relations, a subset of interactions dictating firing order, are altered (Timme & Lapish, 2018). Alone, area left of zero cannot describe causal strength between signals and transfer entropy cannot assess firing order in general. Therefore, employing both metrics in the future would provide a more complete picture of how neuronal firing altered. For example, if area left of zero distributions alter, but not transfer entropy, it is likely that changes in cell dynamics are due to altered firing patterns or altered common input. Note that correlogram peak count and peak times could help confirm whether such a hypothesis is true. Conversely, if area left of zero distributions and transfer entropy both alter, it is likely that the network rewired. Therefore, combining functional connectivity assessment with the metrics in this article can distinguish between different network effects and still be comparable across different biological replicates, recording techniques, and length scales.

The approach in this article facilitates comparison across different networks or recording techniques. It is difficult to relate altered connectivity between neuronal pairs from different cultures, or between two individual neurons and two brain regions because the biological meaning of a connection differs between these different cases (Fedele et al., 2020; Grienberger & Konnerth, 2012; Michel & Brunet, 2019; Obien et al., 2015; Peterka et al., 2011). It is more intuitive to compare whether signal dependence, firing pattern, or firing order changed across length scales or cultures because the biological meaning of these metrics does not change in different cases. However, some functional connectivity metrics, such as transfer entropy, can also be compared across techniques and length scales (Timme & Lapish, 2018). Therefore, more information about dynamics across length scales would be gained by combining algorithm metrics with functional connectivity assessment.

The purpose of this publication was to demonstrate that quantifying correlogram uniformity, peak count, and area left of zero provides useful information. This approach uses fewer assumptions and exhibits greater ease of comparison across length scales, but provides less insight into signal interactions. Therefore, to overcome the limitations of both this algorithm and the literature, algorithm metrics are best used in combination with previous correlogram quantification approaches or transfer entropy. Principles of the quantification in this algorithm are straightforward (Sect. "Correlogram Shape Quantification"-"Algorithm Pipeline"), and therefore can be implemented in the preexisting correlogram quantification software (Hu et al., 2022; Ito et al., 2014; Narayanan & Laubach, 2009; Nex Technologies, 2024; Viejo et al., 2023; Wang et al., 2024) without difficulty. Furthermore, functional connectivity assessment and rhythmic activity quantification are described well in the previous software platforms (Hu et al., 2022; Ito et al., 2011, 2014; Narayanan & Laubach, 2009; Novelli et al., 2019; Wang et al., 2024) and are therefore not difficult to implement in this article’s algorithm. Integrating the different software packages, while ideal, is left to future work.

## Conclusion

Correlograms are a standard method to analyze rasters from recordings of neuronal populations to gain insight into the relationship between a pair of signals. While correlogram shape can provide information about signal independence, firing pattern, and firing order, correlogram shape is typically described qualitatively, limiting the amount of data that can be analyzed. To overcome this limitation, a MATLAB algorithm was developed to automate correlogram shape quantification and thereby gain insight into the relationship between each unique pair of signals from a recording.

The metrics used for quantification were correlogram uniformity, peak count and location, and area left of zero, which respectively quantify signal independence/dependence, firing pattern, and firing order. These metrics were able to describe how relationships between cells altered due to a variety of treatments. Such quantification facilitates comparison of metric distributions and classifications across different regions of the same recording to increase statistical power of correlogram analysis and study signal dynamics with better time resolution. Additionally, comparing metric distributions and classifications between different recordings can help assess variation in treatment responses between biological replicates. Furthermore, correlogram metrics can be used to identify signals of interest for additional experimentation such as immunocytochemistry or electrical stimulation. Additionally, the algorithm performs all correlogram analysis and quantification from signal rasters, and can therefore be applied to any technique in which a raster is generated, including data collected at larger spatial scales such as EEG, or at the cellular level, such as MEA recordings, calcium imaging, or voltage imaging. The fact that algorithm outputs assess changes in signal dependence, pattern, and order, which are features common to any recording regardless of underlying mechanisms, facilitates comparison across different types of recordings. However, algorithm metrics do not assess functional connectivity or provide mechanistic insight beyond indicating the nature of alterations in firing dynamics. It is necessary to combine the metrics in this algorithm with functional connectivity assessment or with results from additional measurements such as imaging, ELISA, and western blotting in order to relate signal timings to connectivity and possible mechanisms. Despite these limitations, algorithm metrics still captured relevant and expected signaling changes in the three TBI-on-a-Chip validation MEA recordings, even when changes were heterogenous. Therefore, the algorithm in this article serves as a useful means of quantifying neuronal dynamics from correlograms from an individual to population level without losing information on network responses or yielding false positive or negative results. This tool can be used provide insight into how neuronal network dynamics are altered by different drugs or conditions, bridge in vitro and in vivo studies, and bridge length scales. The possibility of bridging different scales and techniques makes the algorithm a useful tool to study neuronal dynamics in different conditions or treatments and thereby gain insight into brain function and pathology.

## Supplementary Information

Below is the link to the electronic supplementary material.

Supplementary file 1 (ZIP 48301 KB)

Supplementary file 2 (ZIP 71440 KB)

Supplementary file 3 (DOCX 12492 KB)

## Data Availability

The MATLAB script is available on GitHub (https://github.com/ceadm/Correlogram-Uniformty-Peak-Count-Area-Left-of-Zero.git) as well as in the supplementary material of this work. Recording files are also provided with the code in the supplementary material of this work.
