# Supplementary material for: Quantifying Correlogram Shape to Analyze Neuronal Firing Dynamics Recorded in TBI-on-a-Chip
Source: Neuroinformatics. 2026 Apr 27;24(2):24. doi: 10.1007/s12021-026-09770-9 (PMC13111530; doi:10.1007/s12021-026-09770-9)
Supplement: Supplementary file 1 — (ZIP 48301 KB) [file 12021_2026_9770_MOESM1_ESM.zip › CorrelogramCode/ReadMe.docx]

This code can be employed to study neuronal dynamics from a recording performed with multiple electrodes. This code generates correlograms, calculates correlogram uniformity, peak count, and area left of zero, then generates a variety of figures to show how these metrics, and thereby relative signal timings and neuronal interactions, evolve throughout the recording. Operating principles, code metrics, and output applications are detailed in the original publication (Adam *et. al.* (2025). Quantifying Correlogram Shape to Analyze Neuronal Firing Dynamics Recorded in TBI-on-a-Chip, *Neuroinformatics*. <https://doi.org/10.1007/s12021-026-09770-9>). This document tells you how to run the code and describes code outputs.

**Citations for this Code:**

The following five sources need to be cited when this code is used.

1. The original publication about this code:
   1. <https://doi.org/10.1007/s12021-026-09770-9>
2. The function to read .plx files:
   1. Benjamin Kraus (2026). readPLXFileC (<https://www.mathworks.com/matlabcentral/fileexchange/42160-readplxfilec>), MATLAB Central File Exchange.
3. Violin plot functions:
   1. Holger Hoffmann (2026). Violin Plot (<https://www.mathworks.com/matlabcentral/fileexchange/45134-violin-plot>), MATLAB Central File Exchange. Retrieved February 21, 2026.
   2. Bechtold, Bastian, 2016. Violin Plots for Matlab, Github Project <https://github.com/bastibe/Violinplot-Matlab>, DOI: 10.5281/zenodo.4559847, <https://www.mathworks.com/matlabcentral/fileexchange/170126-violinplot-matlab>
4. Hatchfill (for some of the plot colorations):
   1. Neil Tandon. Hatchfill (<https://www.mathworks.com/matlabcentral/fileexchange/30733-hatchfill>), MATLAB Central File Exchange.

**Terminology:**

This code was written for a lab that does MEA recordings of neuronal networks, called TBI-on-a-Chip, but the analysis pipeline can be applied to any type of recording. Throughout the code variable names and comments, you will see terminology specific to cells. However, here is how to interpret the terminology for any recording in general.

1. Spike = a raster event
2. Cell or Unit = an individual signal

**Setup (first time only):**

1. Activate MATLAB (if first time using MATLAB)
2. Download the code
3. Open MATLAB
   1. After MATLAB opens, you should see the directory and files, the Workspace (shows current variables active in MATLAB, and is usually blank when you first open the program), and the Command Window.  If you open a script like Main_AnalyzeRecordingData.m, an Editor window will also pop up.  You will mostly be using the command window and editor.
   2. Note that descriptions in green text are comments to help follow the code
4. **Currently, the script only reads plx files. If you did not record with .plx, you will need to modify the portion of the code that reads the data accordingly. For plx files, you need to compile the function to read .plx once before running the script.**

*Compiling the function to read plx files:*

1. In MATLAB's directory window, double click on the folder "Function to Read plx Files."  You will now be inside this folder, and will see four files.
   1. The function to read plx files has already been published, and is from the following source.
      1. Benjamin Kraus (2026). readPLXFileC (https://www.mathworks.com/matlabcentral/fileexchange/42160-readplxfilec), MATLAB Central File Exchange.
2. You must compile the read plx function.  You only need to compile once.  The following steps apply for Windows machines...
3. Navigate to the MATLAB Home tab -> Add Ons -> "Get Add Ons" -> Type "MinGW" in the search bar (upper right)
4. Download MinGW-w64 C/C++/Fortran Compiler
5. In the command window, type "build_readPLXFileC" then hit "Enter"
6. A message that reads, "Building with 'MinGW64 Compiler (C)'." will appear, some time will pass (a few seconds), then "MEX completed successfully." will appear.
   - - If 4 does not proceed as planned, and red writing appears, an error has occurred.  Adjust accordingly, based on the nature of the error and try again.

After compiling the function to read recording files, the only code with which you need to interact is **Main_AnalyzeRecordingData.m**. The functions called by this main script can be found in the “Analysis Execution” folder and are commented with explanations for those who desire to examine the functions in more depth.

Note that a function called PlotCorrelogramGeneration is also provided so that users can recreate figure1 of the original publication (<https://doi.org/10.1007/s12021-026-09770-9>) if desired.

**Analyzing a Recording:**

 The code consists of several folders.

- "Analysis Execution" contains all the functions necessary to analyze the recording data.
- "Function to Read plx Files" contains the function (provided by Benjamin Kraus (2026). readPLXFileC https://www.mathworks.com/matlabcentral/fileexchange/42160-readplxfilec, MATLAB Central File Exchange) to read recording data into MATLAB.
- "Output Files" is where MATLAB saves the code outputs after all the data is processed (for example, after generating correlograms for the recording, the correlograms are saved in this directory).
  1. Example outputs used in the original publication are provided as examples.
- "Raw Recordings" is the folder where you should place the recording .plx files that you wish to analyze.
  1. The raw recording files used in the original publication are provided as examples.

**The function "Main_AnalyzeRecordingData.m" is not in any of these subfolders, and is what you should open to start data analysis.** It is the only script that allows the user to define analysis parameters and with which you must interact.

To analyze recording data...

1. Place the .plx file in the "Raw Recordings" folder
2. Open "Main_AnalyzeRecordingData.m"
   1. Be sure that the file name is set to the recording you wish to analyze. Do NOT add “.plx” at the end of the file name.
   2. In Main_AnalyzeRecordingData.m, you will see a list of parameters that you can control.  This list includes: the .plx file name (should be the same as the recording .plx file name), activation or inactivation of optional code outputs (aka "switches", saved in the structure “sw,” set these to 1 if you want to use certain code features, 0 if you only want the default outputs), plot appearance options (for example, font size, font type, etc...), and correlogram options (time range of the correlogram, number of bins, and classification options). Plot appearance and correlogram parameters are saved in the plot properties structure “plotProps.”
   3. Each user controlled parameter is explained in a comment next to the parameter. See table 1 of the original publication for more details beyond the comments.
3. After all parameters are set to your satisfaction, hit the green "Run" button
4. The code will ask you several prompts in the command window.
   1. Read the prompts and answer accordingly. The prompts only need to be followed the first time a recording is analyzed, and will be loaded in subsequent running of the script for that recording file unless otherwise specified by the user (in sw from 2b).
5. Once the prompts are complete, correlograms will be generated in each region.
6. Figures will appear when the script is finished running.  To save the figures, click "File" -> "Save As" and save the figure however and wherever you wish.

Note that all violin plots generated by the code rely on the violin plot function found at the following links. <https://www.mathworks.com/matlabcentral/fileexchange/45134-violin-plot>. Bechtold, Bastian, 2016. Violin Plots for Matlab, Github Project
<https://github.com/bastibe/Violinplot-Matlab>, DOI: 10.5281/zenodo.4559847, <https://www.mathworks.com/matlabcentral/fileexchange/170126-violinplot-matlab>

**Summary of Code Outputs besides Figures:**

*Optional whether to view these or not:* Code outputs are saved as .mat files in the “Output Files” directory in a folder with the same name as the recording. There are nine different outputs, as follows (listed alphabetically).

- 1. **AnalysisRegions** = a structure with three entries containing information about the regions in which correlograms were generated
     1. AnalysisRegions.Minutes = minute where each region started and ended, rows = each region, first column = start minute, second column = end minute
     2. AnalysisRegions.Seconds = AnalysisRegions.Minutes.*60 so units are seconds instead of minutes
     3. AnalysisRegions.numRegions = the number of analysis regions in the recording
  2. **badRegions** = a structure with three entries containing information about portions of the recording that you wish to exclude from analysis (for example, recording during unit selection before signals were fully established)
     1. badRegions.badRegionsIndicator = logical indicating whether any portion of the recording should be excluded from analysis
     2. badRegions.badStartTime = start time(s), in seconds, of the recording region(s) to exclude from analysis (only exists if part of the recording was excluded)
     3. badRegions.badEndTime = end time(s), in seconds, of the recording region(s) to exclude from analysis (only exists if part of the recording was excluded)
  3. **badSignals**
     1. A logical array that is 1 for signals to be ignored and excluded from analysis, 0 otherwise. If there are no signals to be excluded, the array is all 0 (false).
  4. **Correlograms** = a structure with correlogram details in each analysis region
     1. Correlograms.CorrelogramBins = the correlogram bin centers (in seconds)
     2. Correlograms.mat also contains additional substructures for each analysis region, named Region1, Region2, … RegionN. In these substructures, there are five different variables.
        1. CorrelogramProbabilities = the probability a spike falls in each correlogram bin within the given analysis region. Each row is a correlogram bin and each column is the correlogram of a particular comparison vs. reference signal.
        2. ComparisonNames = a cell array that tells you the comparison vs. reference signal names
        3. numberOfSpikesContributingToTheCorrelogram = Total correlogram event count (bin counts are divided by this number to obtain probability, so multiplying by this number gives the raw correlogram counts)
        4. CellAsComparisonInds = tells you the columns of CorrelogramProbabilities where each signal was used as a comparison to generate the correlogram. Row 1 -> N of this structure corresponds to signal 1 -> N.
        5. CellAsReferenceInds = tells you the columns of CorrelogramProbabilities where each signal was used as a reference to generate the correlogram. Row 1 -> N of this structure corresponds to signal 1 -> N.
  5. **InjuryIndicies** = a structure containing five entries with information about treatments administered during the recording. Note that this structure mentions “injury” because the Shi lab from which this code originates studies the effect of injuries administered during a recording. However, any treatment (not just injury) is saved in this structure.
     1. InjuryIndicies.InjuryStartMin = minute where each injury/treatment started, rows = each injury/treatment, first column = start minute
     2. InjuryIndicies.InjuryEndMin = minute where each injury/treatment ended, rows = each injury/treatment, first column = end minute
     3. InjuryStartSec and InjuryEndSec are the same as 1 and 2, but in units of seconds instead of minutes
     4. InjuryIndicies.InjuryLabels = a cell listing treatment/injury names
     5. AnalysisRegions.NumberOfInjuriesOrTreatments = the number of different injuries/treatments in the recording
  6. **plotProps** = a structure that lists the user specifications (see details in 2b or comments in Main_AnalyzeRecordingData.m), for plot appearance controls and correlogram properties
  7. **RawData** = a structure of saved raw output of readPLXFileC.
  8. **ProcessedData** = a structure containing 14 entries that list the results of reading RawData to obtain signal names, rasters, and metrics independent of correlograms
     1. ProcessedData.Comment = saves any comment written during recording in the .plx file
     2. ProcessedData.ExperimentTotalGain = array listing the gain of each electrode
     3. ProcessedData.CellIDs = list of signal names (index 1 = signal 1, index 2 = signal 2, …, index N = signal N)

All names are written as “s” for signal, followed by a number which tells you the electrode from which the signal originated, followed by a, b, c, or d to indicate different signals on the same electrode. A maximum of four signals can be discriminated per electrode.

- - 1. ProcessedData.CellDisplayNames = list of how signal names were displayed in the raw data (can differ from the signal names because some recordings originally list names as “dsp” for “display” and number signals by the order in which they were sorted, used for debugging only)
    2. ProcessedData.Raster = cell array where one entry is the list of times a particular signal fired for the entire recording. List 1 (ProcessedData.Raster{1}) corresponds to signal 1, … , list N (ProcessedData.Raster{N})corresponds to signal N.
    3. ProcessedData.CellCount = total number of signals in the recording
    4. ProcessedData.NumberOfActiveElectrodes = the number of electrodes that have at least one signal
    5. ProcessedData.TotalNumberOfElectrodes = the total number of electrodes from which a signal could be recorded (should be larger than or equal to the NumberOfActiveElectrodes)
    6. ProcessedData.ExperimentEndTimestamp = recording end (index – divide by the sampling frequency of the recording to obtain the recording end time in seconds, note this is the same as RawData.LastTimestamp)
    7. ProcessedData.ExperimentSamplFreq = sampling frequency of the recording
    8. ProcessedData.ExperimentEndTime = recording end (in seconds)
    9. ProcessedData.FiringCountOfEachCell = total number of times each signal fired during the recording
    10. ProcessedData.SpikeIntervals = cell array where one entry is a list of time elapsed since the previous spike for the entire recording of a given signal. List 1 (ProcessedData.SpikeIntervals {1}) corresponds to signal 1, … , list N (ProcessedData.SpikeIntervals {N}) corresponds to signal N.
    11. ProcessedData.RegionStats = a substructure with five entries to store information for firing count and interval histograms in particular regions of the recording (relative to treatment, or in each analysis region, as specified by the user)
        1. SCBins = bin centers to plot the spike count histograms
        2. SIBins = bin centers to plot the spike interval histograms
        3. groupOrder = description of each region being analyzed (label 1 corresponds to region 1, label 2 to region 2, … label N to region N)
        4. timeRange = second in the recording where each region started and ended, rows = each region, first column = region start (s), second column = region end (s)
        5. Substructures (one per region) with five entries describing region-specific information
           1. spikesInRegion = a cell array that is 1 x the number of signals and saves a binary array that is 1 if an event occurred in the given region, 0 if not. Entry 1 corresponds to signal 1, … , entry N (ProcessedData. Region#.spikesInRegion{N}) corresponds to signal N.
           2. regionSpikeCount = array that is 1 x the number of signals and lists the number of spikes each signal produced in the given region divided by the region duration (division performed in order to normalize data for regions of different length)
           3. fractionOfSpikesThatOccurrInThisRegion = array that is 1 x the number of signals and lists the fraction of each signal’s total spike count that occurred in the given analysis region
           4. INDS_interspikeIntervalsInRegion = a cell array that is 1 x the number of signals and saves a binary array that is 1 if a given interspike interval occurred in the given region, 0 if not. Entry 1 corresponds to signal 1, … , entry N (ProcessedData.Region#.spikesInRegion{N}) corresponds to signal N.
           5. interspikeIntervalsInRegion = a cell array that is 1 x the number of signals and lists the interspike intervals that occurred in the given region for each signal. Entry 1 corresponds to signal 1, … , entry N (ProcessedData. Region#.spikesInRegion{N}) corresponds to signal N.
  1. **RecordingMetrics** = structure with six entries that saves statistics, correlogram metrics, and classifications
     1. meanSPM = mean (of all signals) number of spikes in each minute of the recording
     2. stdSPM = standard deviation (of all signals) in the number of spikes in each minute of the recording
     3. minBinsSPM = minutes of the recording used for plotting spikes per minute (does not include 0 because spikes that occur between minute 0 and 1 contribute to the spike count of minute 1)
     4. individualSPM = number of spikes emitted by each signal in each minute of the recording
     5. minBins = all minutes of the recording, including minute 0
     6. CorrelogramMetrics = substructure containing five entries detailing correlogram classifications and statistics
        1. RefLabels = list of reference cell names (applies for plot labeling, and, if different from the list of cell names, tells which specific signals were used as references)
        2. refIndicies = indices of reference signals (i.e. cell 1, 7, 16 would be saved in refIndicies and correspond to names s4a, s4b, and s11a)
        3. maxPkCount = highest peak count of all correlograms in the recording
        4. Region Substructures (one for each analysis region) = substructure with 11 entries listing correlogram metrics in each analysis region
           1. leaderProb = array of area left of zero for each correlogram
           2. followerProb = array of area right of zero for each correlogram (note: leaderProb+followerProb = 1)
           3. uniformityTest = logical array indicating whether each correlogram was uniform or not
           4. uniformityTestPValue = array of Chi squared p-value used to decide the uniformityTest logical
           5. correlogramPeaks = cell array listing the peak values of each correlogram
           6. correlogramPeakLocations = cell array listing the peak locations in each correlogram
           7. numberOfCorrelogramPeaks = double array listing the peak count of each correlogram
           8. leaderProbMat = matrix of correlogram area left of zero (rows = comparison signal vs. columns = reference signal), used only if plotting correlogram metric heatmaps
           9. numPeaksMat = matrix of correlogram peak count (rows = comparison signal vs. columns = reference signal), used only if plotting correlogram metric heatmaps
           10. unifTestMat = matrix of correlogram uniformity (rows = comparison signal vs. columns = reference signal), used only if plotting correlogram metric heatmaps
           11. unifTestpMat = matrix of correlogram Chi-squared p-value used to determine correlogram uniformity (rows = comparison signal vs. columns = reference signal), used only if plotting correlogram metric heatmaps
        5. RegionDifferences = a substructure evaluating the change in correlogram metric values across analysis regions. Note that the field name indicates which earlier region is subtracted from which later region (i.e. Region7_Minus_Region1 subtracts metric values in analysis region 1 from those of analysis region 7).
           1. leaderProbDifference = matrix showing the difference in correlogram area left of zero (later region area left of zero – earlier region area left of zero)
           2. uniformityDifference = matrix showing the difference in correlogram uniformity (later region uniformity – earlier region uniformity)
           3. peakCountDifference = matrix showing the difference in correlogram peak count (later region peak count – earlier region peak count)
