# Supplementary material for: Quantifying Correlogram Shape to Analyze Neuronal Firing Dynamics Recorded in TBI-on-a-Chip
Source: Neuroinformatics. 2026 Apr 27;24(2):24. doi: 10.1007/s12021-026-09770-9 (PMC13111530; doi:10.1007/s12021-026-09770-9)
Supplement: Supplementary file 1 — (ZIP 48301 KB) [file 12021_2026_9770_MOESM1_ESM.zip › CorrelogramCode/~WRL2723.tmp]

**Setup (first time only):**

1. Activate MATLAB
2. Download the code
3. Open MATLAB by double clicking Main.m. This script is the only script you will need to interact with.
   1. After MATLAB opens, you should see the directory and files, the Workspace (shows current variables active in MATLAB, and is usually blank when you first open the program), and the Command Window.  If you open a script like Main.m, an Editor window will also pop up.  You will mostly be using the command window and editor.
   2. Note that green text are comments to help follow the code
4. In MATLAB's directory window, double click on the folder that says "Function to Read plx Files."  You will now be inside this folder, and will see four files.
   1. The function to read plx files has already been published, and is from the following source.
      1. Benjamin Kraus (2026). readPLXFileC (https://www.mathworks.com/matlabcentral/fileexchange/42160-readplxfilec), MATLAB Central File Exchange.
5. You must compile the read plx function.  You only need to compile once.  The following steps apply for Windows machines...
6. Navigate to the MATLAB Home tab  → Add Ons -> "Get Add Ons" -> Type "MinGW" in the search bar (upper right)
7. Download MinGW-w64 C/C++/Fortran Compiler
8. In the command window, type "build_readPLXFileC" then hit "Enter"
9. A message that reads, "Building with 'MinGW64 Compiler (C)'." will appear, some time will pass (a few seconds), then "MEX completed successfully." will appear.
   - - If 4 does not proceed as planned, and red writing appears, an error has occurred.  Adjust accordingly, based on the nature of the error and try again.

**Data Analysis:**

Navigate to the folder named  "Current Code."  You will see several folders.

      "Analysis Execution" contains all the functions necessary to analyze the recording data

      "Function to Read plx Files" contains the function (provided online and cited in the script) to read recording data into MATLAB

      "Output Files" is where MATLAB saves the files after all the data is processed (for example, after generating correlograms for the recording, the correlograms are saved here)

      "Raw Recordings" is the folder where you should place the recording .plx files that you wish to analyze

The function "Main_AnalyzeRecordingData" is what you should open to start data analysis.  It is also the only script with which you must interact.

To analyze recording data...

1. Place the .plx file in the "Raw Recordings" folder
2. Open "Main_AnalyzeRecordingData.m"
   1. You will see a list of parameters that you can control.  This list includes the .plx file name (should be the same as the recording .plx file name), activation or inactivation of optional code outputs (aka "switches", set these to 1 if you want to make certain figures, 0 if you only want the default figures), plot appearance options (for example, font size, font type, etc...), and correlogram options (time range of the correlogram, number of bins, and a threshold number of spikes contributing to the correlogram below which you wish to ignore correlogram data)
   2. Be sure that the file name is set to the recording you wish to analyze
3. After all parameters are set to your satisfaction, hit the green "Run" button
4. The code will ask you to select regions of the data to analyze, and you will need to follow the prompts in the command window
   1. Once regions are selected, they will be loaded in subsequent running of the script for that recording file, unless otherwise specified
5. Once regions are selected, correlograms will be generated in each region
6. Figures will appear when the script is finished running.  To save the figures, click "File" -> "Save As" "Enhanced metafile (*.emf) and save the figure wherever you wish.

Find me for any questions, or if you get a red error message in the command window.
