# Supplementary material for: Quantifying Correlogram Shape to Analyze Neuronal Firing Dynamics Recorded in TBI-on-a-Chip
Source: Neuroinformatics. 2026 Apr 27;24(2):24. doi: 10.1007/s12021-026-09770-9 (PMC13111530; doi:10.1007/s12021-026-09770-9)
Supplement: Supplementary file 3 — (DOCX 12492 KB) [file 12021_2026_9770_MOESM3_ESM.docx]

**Supplementary Material**

**Article Title:** Quantifying Correlogram Shape to Analyze Neuronal Firing Dynamics Recorded in TBI-on-a-Chip

Casey Erin Adam^1,2^, Shatha J. Mufti^1,2,3^, Jhon Martinez^1,2,3^, Edmond A. Rogers^1,4^, Martina Dalolio^1,3^, Nikita Krishnan^1,2,3^, Timothy Beauclair^1,2,3^, Riyi Shi^1,2,3 *^

1. Center for Paralysis Research, Purdue University, West Lafayette, IN 47907, USA
2. Department of Basic Medical Sciences, School of Veterinary Medicine, Purdue University, West, Lafayette, IN 47907, USA
3. Weldon School of Biomedical Engineering, Purdue University, West Lafayette, IN 47907, USA
4. Indiana University School of Medicine, 340 West 10th Street, Fairbanks Hall, Suite 6200, Indianapolis, IN 46202-3082, USA

^*^ Corresponding Author

**Email:** [riyi@purdue.edu](mailto:riyi@purdue.edu)

**ORCID List:**

Riyi Shi: <https://orcid.org/0000-0002-7297-9428>

Jhon Martinez: <https://orcid.org/0009-0007-3762-3446>

Martina Dalolio: <https://orcid.org/0000-0002-6108-1348>

Nikita Krishnan: <https://orcid.org/0000-0001-8371-3951>

# **Contents:**

Section S1: Impact without Bicuculline …………………….……..…….……..……….…... Page 2

Section S2: Supplementary Figures ……………….……..…….……….……..…...….…… Page 15

Section S3: Description of Supplementary Movies .…….….…….……..…..……….….… Page 23

Section S4: Literature Cited ……….………………….……..………….……..………….…. Page 25

# **Section S1: Impact without Bicuculline**

One of the microelectrode array (MEA) recordings used to validate code outputs in the main text was a six hour recording of cell activity before and after the culture was injured *via* acceleration impact with a total of five 250 g impacts (main text Sect. “MEA Recording to Validate Algorithm Outputs”, Sect. “Results”, Discussion Sect. “Metric Interpretations for Impact Injury”, Figs. 4, 5 and 10, Supplementary Movies S4-S6, and Supplementary Figs. S7, S9, and S12). This impact culture was also treated with 40 μM Bicuculline Methiodide prior to the beginning of the recording (Sigma-Aldrich 14343-10MG, added to the culture medium in the life support reservoir depicted in main text Fig. 3A) to standardize cell firing patterns (G. Gross, 1995; G. W. Gross et al., 1997). In general, alterations in correlogram properties before vs. after impact were subtle, both for algorithm metrics and literature measurements (Rogers & Gross, 2019). As discussed throughout the main text, one possible explanation for this lack of change is that alterations in cell signaling due to bicuculline overrode alterations due to impact. To test whether this hypothesis is true, an MEA recording of a similar impact without bicuculline was analyzed with the MATLAB algorithm.

Roughly 2.8 hours into the recording, a culture was subjected to impact injury *via* an impact pendulum as previously described (Rogers et al., 2022, 2023; Rogers & Gross, 2019). Ten 200 g impacts were administered. After impact, the culture was subjected to three different treatments hypothesized to have little to no effect on network dynamics, in order to compare changes across treatments. First, roughly 26 minutes after impact, was a partial medium leak from the recording chamber. Leak volume was too low to accurately measure, and consisted of only a few drops of medium. The purpose of this treatment was to ensure that loss of a few drops of medium did not affect firing in the culture. The second treatment was administered roughly one minute after the medium leak was stopped, when a subeffective dose of bicuculline methiodide was administered to the culture (0.2 μM, Sigma-Aldrich 14343-10MG, added *via* the drug injection port depicted in main text Fig. 3A). This concentration of bicuculline usually does not dramatically alter culture activity (Gramowski et al., 2006). However, due to increased epileptiform activity after injury (Atlan & Margulies, 2019; Ianof & Anghinah, 2017), subeffective doses of bicuculline may influence injured cultures more than uninjured. The final treatment was another dose of 0.2 μM bicuculline methiodide to determine whether subeffective doses were cumulative. Culture activity was recorded for a total of eight hours, then the recording was analyzed via the MATLAB script. Culture firing was recorded with a sampling frequency of 40 kHz. One electrode had a noisier signal than the others. To reduce the effects of noise, the total gain of the noisy electrode was reduced to 7,000. For all other electrodes, the total gain was 10,000. A total of 41 unique signals were recorded.

Supplementary Fig. S1 shows algorithm outputs independent of correlograms. The raster relative to impact is shown in Supplementary Fig. S1A, and differs before vs. after impact (Supplementary Fig. S1A insets). The mean number of spikes per minute is shown in Supplementary Fig. S1B. Impact decreased the mean number of spikes per minute from ~100 spikes/min before impact to ~60 spikes/min after impact. During the medium leak, average culture firing rate increased to ~90 spikes/minute. The first administration of 0.2 μM bicuculline temporarily increased mean firing rate to ~100 spikes/min, but within 15 minutes of bicuculline administration, firing had decreased back to the post-impact baseline of ~60 spikes/min. The second dose of 0.2 μM bicuculline also caused a temporary increase in culture firing to ~100 spikes/minute, but this effect lasted only for a few minutes before returning to the post-impact baseline of ~60 spikes/min. Together, these observations suggest that firing rate was sensitive to all treatments. Additionally, regardless of treatment, individual spike counts per minute (lighter lines in Supplementary Fig. S1B) show that most cells fired less than the average, and that shifts in the average spikes per minute were due to changes in specific signals, not the entire cell population.

Supplementary Fig. S1C shows firing count histograms for the entire recording as well as between each treatment. For the entire recording, all cells in the recording fired between 1,000-100,000 times. Firing counts between each treatment were most affected by impact injury. Note that, to prevent different amounts of time between treatments from biasing the histograms between each treatment, the spikes/minute in Supplementary Fig. S1C are calculated by taking the total number of spikes for each cell in each period between treatments and dividing by the total amount of time in each period (min). The counts/min in Supplementary Fig. S1C therefore do not correspond to the average spikes per minute from Supplementary Fig. S1B, but are instead normalized to account for differences in the amount of time between treatments. Before impact, individual cells fired between 0.02 and 13.5 times/min, with a mean of ~1.5 spikes/min. After impact, firing count histograms broadened. Cells still fired on average ~1.5 spikes/min, but this count ranged between 0.0006 and 13 times/min, with only a single signal firing ~0.0008 times/min. After medium leak, distributions still had an average of ~1.5 spikes/min, but ranged between 0.009 and 13 times/min. Therefore, the increase in mean spikes/minute due to medium leak was the result of the previously less active signal firing more, and is not a population level change. This activity restoration may be due to individual signals recovering after impact rather than due to a change caused by the leak. Additionally, firing count distributions were similar for each administration of bicuculline, and matched the post-impact distribution. Together, these observations show that the biggest changes in cell firing were the result of impact, and the other treatments had minimal to no effect. Therefore, impacted cultures without bicuculline exhibit a greater change in firing count distributions after impact (Supplementary Fig. S1C) than impacted cultures under bicuculline (main Sect. “Outputs Independent of Crosscorrelations”, Fig. 4 and 5).

| 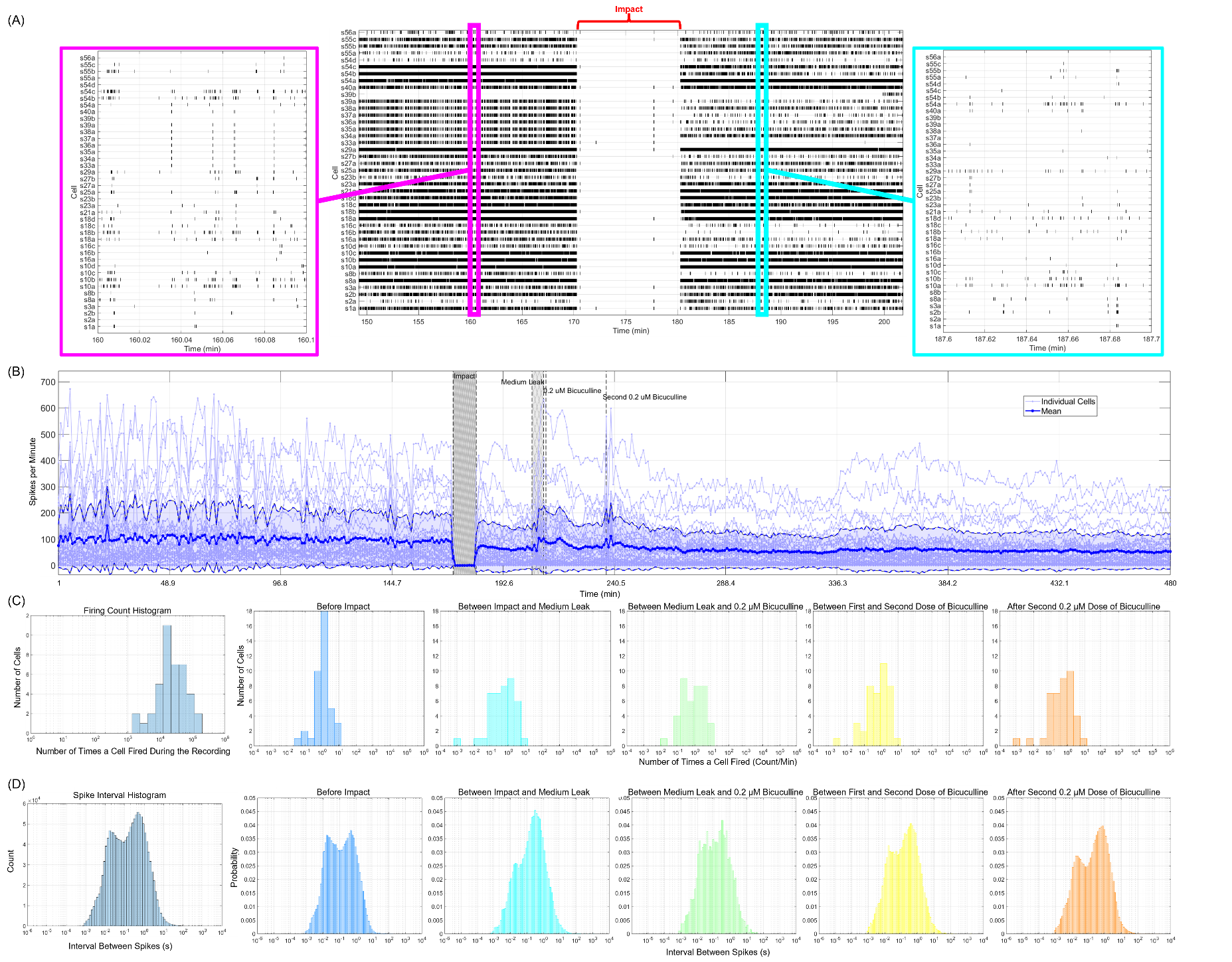 |
| --- |
| **Fig. S1. Impact culture firing metrics independent of correlograms.**  **Panel A** shows signal rasters before and after impact (indicated in red). Insets show rasters before (magenta) and after (cyan) impact over a smaller range in time. Each row represents a unique signal, corresponding to a single cell, and black bars indicate times when the given cell fired. **Panel B** shows the population mean number of spikes per minute (dark blue line), the population standard deviation in the number of spikes per minute (shading), and the number of spikes per minute of individual signals (lighter lines). **Panel C** shows firing count histograms for the entire recording as well as between treatments. **Panel D** shows interspike interval distributions for the entire recording as well as between treatments. The histograms in panel B and C are normalized to account for different amounts of time between different treatments. |

Supplementary Fig. S1D shows interspike interval distributions for the entire recording and between treatments. For the entire recording, interspike interval distributions were bimodal, with a peak occurring at roughly 16.5 ms and another at roughly 0.48 s. The distribution before impact matched the overall recording distribution. After impact, the interspike interval distribution became unimodal. The peak at shorter times nearly disappeared, showing that cells fired less frequently after impact, and the peak at longer times shifted from 0.48 s before impact to 0.32 s, suggesting a change in rhythmic firing pattern. A similar decrease in the mean rhythmic activity interval was observed in the impact recording under bicuculline (main Sect. “Outputs Independent of Crosscorrelations”, Fig. 5C). Distributions after medium leak were still unimodal, but showed a slight restoration in histogram counts of smaller interspike intervals. This observation is likely due to the recovery of individual signals after impact, as discussed for the firing count histograms (Supplementary Fig. S1C), and not the result of medium leak. After bicuculline, interspike interval histograms became bimodal, with a peak occurring at roughly 16.5 ms, and another at roughly 0.48 s after the first dose and 0.74 s after the second dose. This observation supports the notion that subeffective doses of bicuculline can alter firing in injured cultures.

After plotting the distributions in Supplementary Fig. S1, the algorithm divided the recording into 66 analysis regions, each seven minutes in length. These regions are shown in Supplementary Fig. S2A. Correlograms ranging $\pm$ 1 s with a 1 ms bin width around $t=0$ were created for each analysis region, then correlogram metrics were calculated. Supplementary Fig. S2 also shows how correlogram uniformity, peak count, and area left of zero distributions changed across analysis regions. Changes in individual correlograms can be viewed in Supplementary Movies S7-S9. As shown in Supplementary Fig. S2B and C, the amount of uniform correlograms increased as a result of impact, and this change was unaffected by the other treatments. Peak count remained unaffected by treatment (Supplementary Fig. S2D). Peak times (Supplementary Fig. S2E) remained fairly constant, save for the regions when bicuculline was administered. When bicuculline was administered, the number of peaks with times outside the range of -0.1-0.1 s decreased. As shown in Supplementary Fig. S2F, correlogram area left of zero was only affected by impact, and none of the other treatments. Before impact, most correlograms exhibited an area left of zero between 0.4-0.6. After impact, most correlograms had an area left of zero between 0.3-0.7, with more outliers outside this range. This observation suggests that firing order became more consistent as a result of impact injury. The other treatments administered to the culture did not alter area left of zero distributions from those post-impact.

| 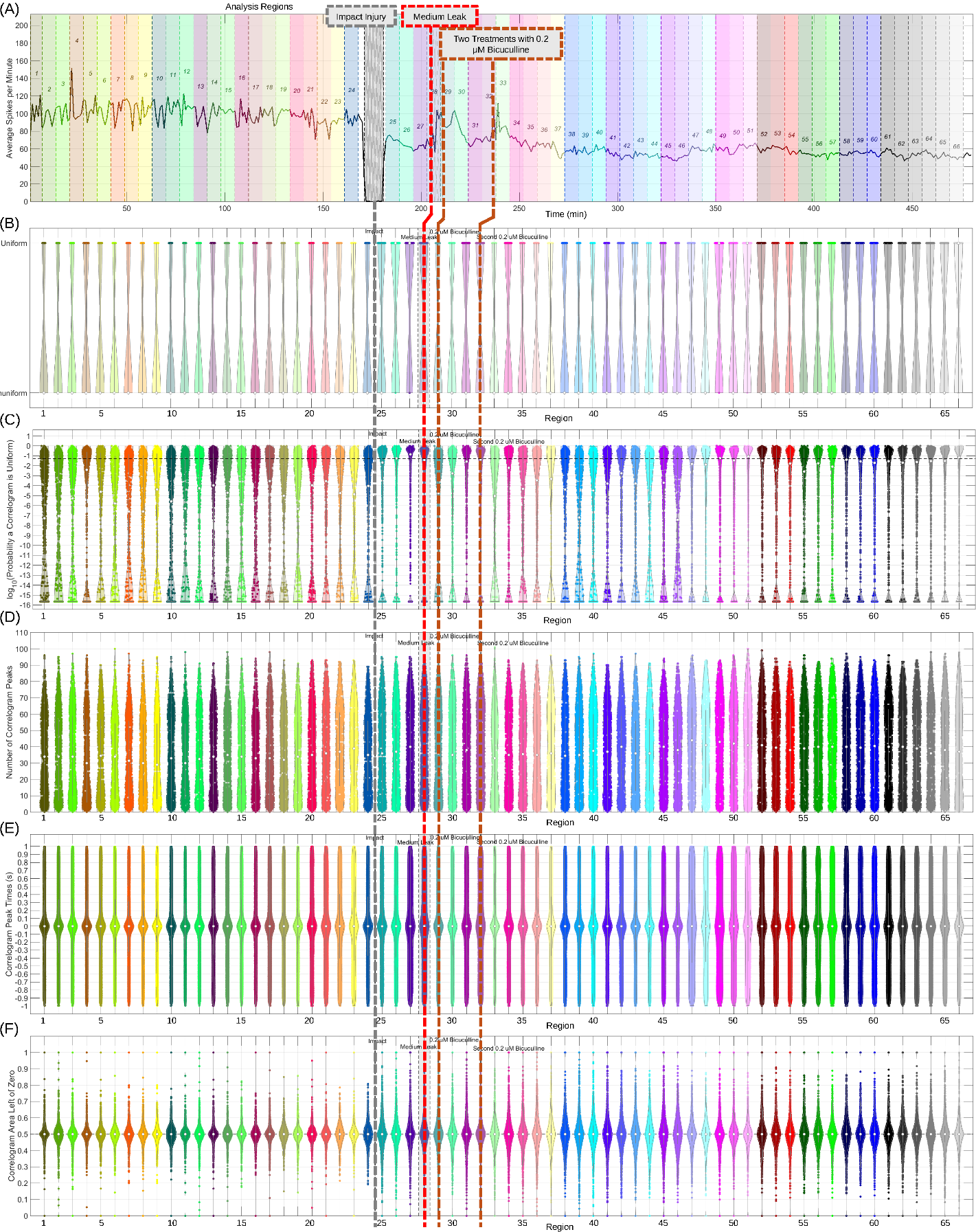 |
| --- |
| **Fig. S2.**  **Correlogram metric distributions as a result of impact.**  **Panel A** shows the analysis regions in which correlograms were generated for the entire recording overlayed over the mean spike count per minute. Violin plots are displayed to view population level changes in correlogram properties across analysis regions. Correlogram uniformity and probability of being uniform are shown in **panel B and C**, peak count and peak times in **panel D and E**, and area left of zero in **panel F**. Individual correlogram metrics for each analysis region are shown in Supplementary Movies S7-S9 and correspond to individual points in the violins. White points in the violins represent the median metric value. |

To increase the ease by which changes in correlogram metrics are visualized, metrics were classified as described in main text Sect. “Correlogram Shape Quantification”. The fraction of correlograms with each classification are shown in Supplementary Fig. S3. As shown in Supplementary Fig. S3A, impact decreased the faction of correlograms with a nonuniform distribution from ~0.65 to between 0.42 and 0.50. Medium leak had no effect on uniformity. Both doses of bicuculline briefly elevated the fraction of nonuniform correlograms from post-impact values to roughly 0.65. However, these changes only lasted until analysis region 49, where the fraction of nonuniform correlograms returned to ~0.50. As shown in Supplementary Fig. S3B, peak count classifications did not change dramatically due to any of the treatments administered in the recording. However, an increasing number of correlograms had zero peaks after impact, suggesting that correlograms broadened as a result of impact, and the decreased peak prominence was enough to prevent flattened peaks from being detected. Such broadening and decrease in peak prominence has previously been observed after impact (Rogers & Gross, 2019), and these results therefore agree with the literature. As shown in Supplementary Fig. S3C, impact decreased the fraction of correlograms with weak leader/follower dynamics, and increased the fraction of correlograms with other classifications, especially with fairly weak classifications. The other treatments during the recording had minimal effect on firing order classifications.

| 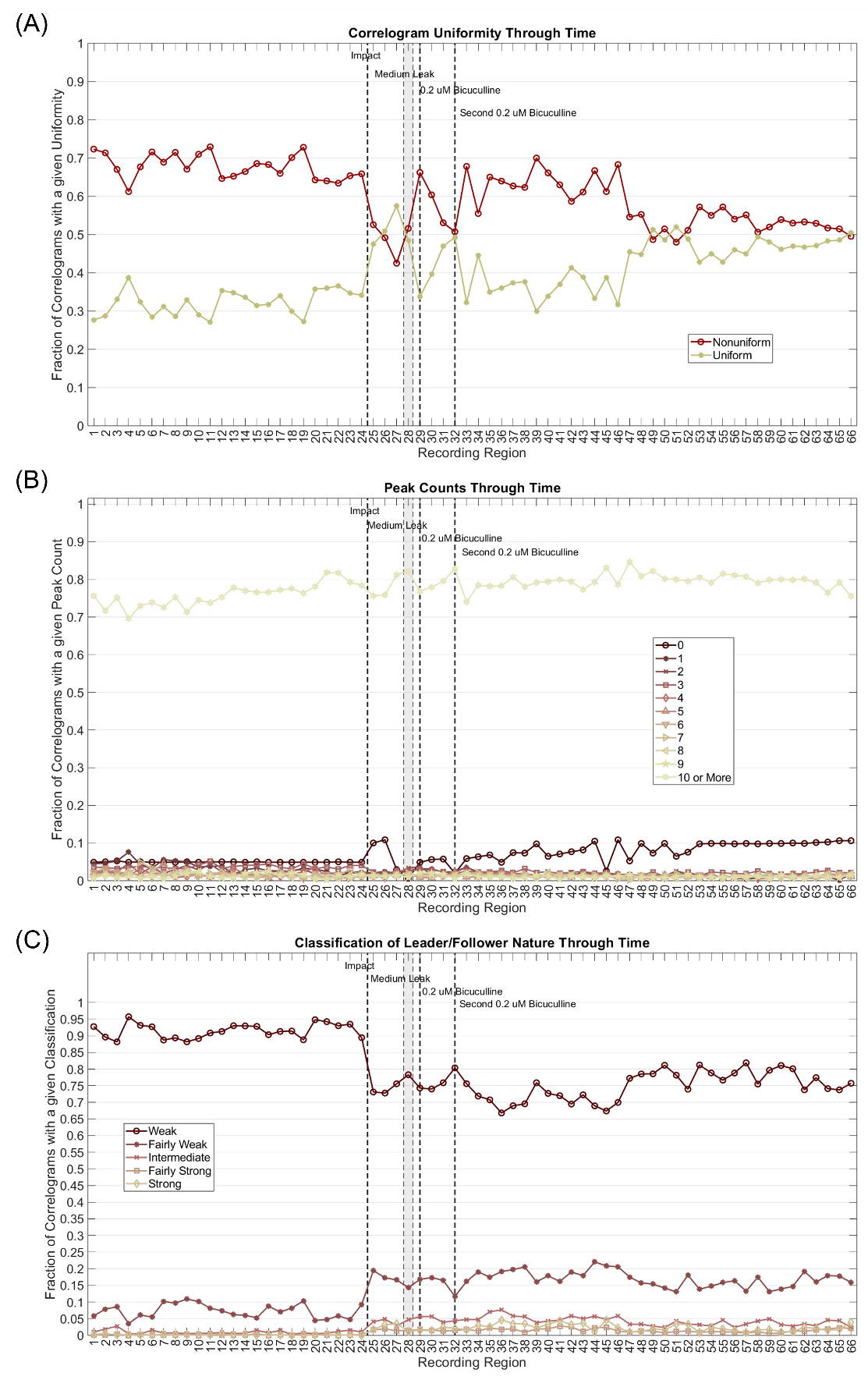 |
| --- |
| **Fig. S3. Correlogram classification summaries after impact without bicuculline.** Correlogram uniformity (**panel A**), peak count (**panel B**), and leader/follower nature (**panel C**) were classified as described in main text Sect. “Correlogram Shape Quantification”. The fraction of correlograms with each classification are shown for each region of the recording in which correlograms were generated. |

Classifications of correlogram uniformity, peak count, and area left of zero were also combined to observe how overall correlogram shape evolves. As mentioned in main text Sect. “Correlogram Classification Heat Maps”, there are 110 different combinations of the three classifications, two uniformity classifications x five leader/follower strength classifications x 11 peak count classifications. Supplementary Fig. S4 shows the percent of correlograms with each classification combination plotted by periods relative to treatments. Supplementary Table S1 summarizes changes in the most prominent correlogram classification groupings that arose as a result of impact injury. In general, as expected from the results in Supplementary Fig. S3A and C, the number of groupings including fairly weak and uniform correlograms increased after injury. After the medium leak, heatmap values of weak leader/follower correlograms with 10 or more peaks shifted toward pre-impact levels, and remained stable regardless of bicuculline treatment. Heatmap values of fairly weak leader/follower correlograms with 10 or more peaks maintained the post-impact values, suggesting that these correlograms, were permanently altered by impact injury. Therefore, this impact may have damaged connections between cells, rendering firing more independent.

| **Supplementary Table S1.** Changes in the most prominent correlogram classification groupings, shown in Supplementary Fig. S4, as a result of impact injury. | | |
| --- | --- | --- |
| Grouping | Before Impact | After Impact |
| Nonuniform  10 or more peaks  Weak leader/follower | 44.0% | 29.3% |
| Uniform  10 or more peaks  Weak leader/follower | 27.7% | 34.9% |
| Nonuniform  10 or more peaks  Fairly weak leader/follower | 2.4% | 5.4% |
| Uniform  10 or more peaks  Fairly weak leader/follower | 4.7% | 10.2% |
|  | Total = 78% | Total = 79.8% |

| 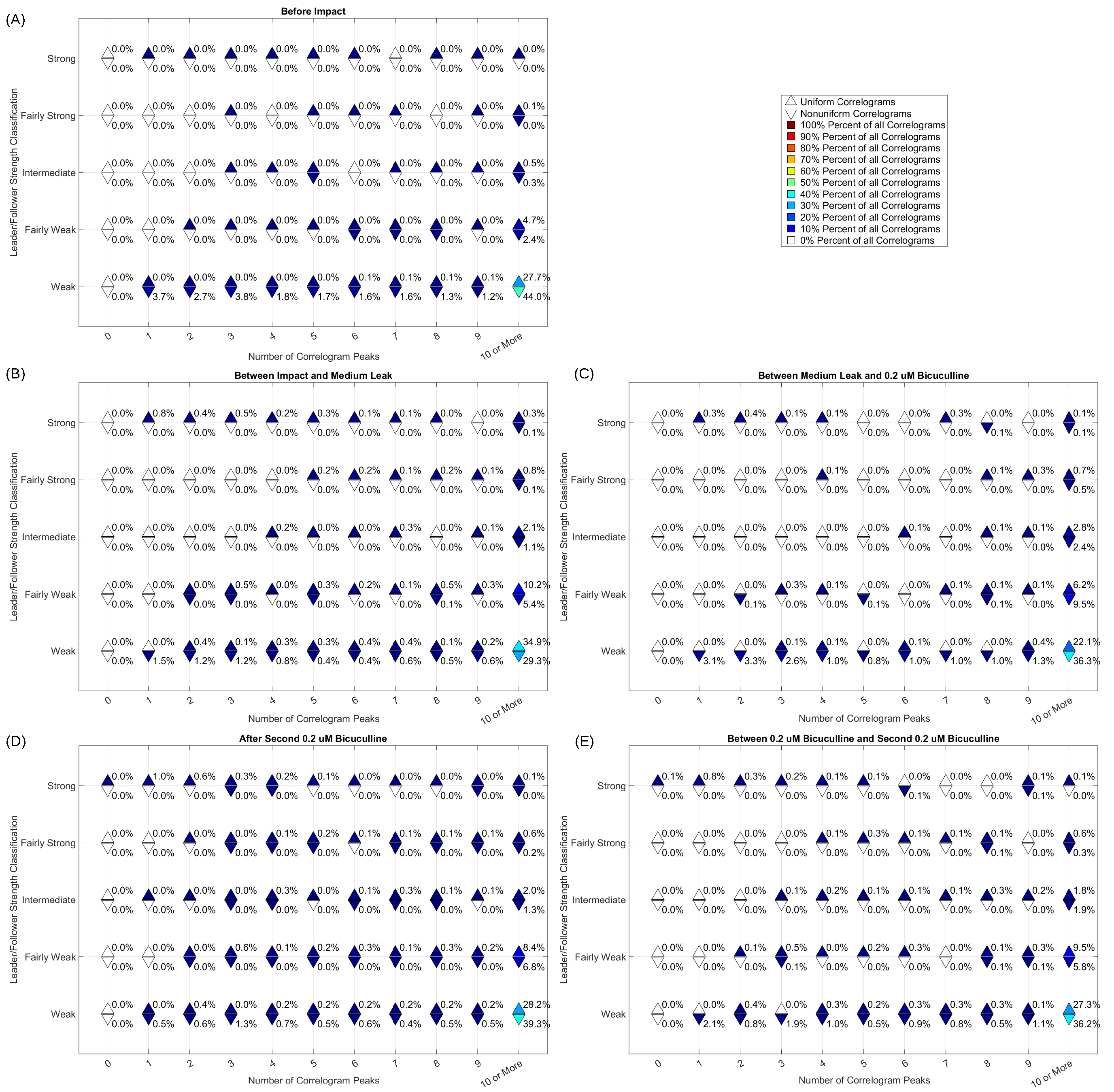 |
| --- |
| **Fig. S4.** Heatmaps showing the percent of correlograms with each classification grouping for a culture before any treatment (**panel A**), after impact (**panel B**), during and after medium leak (**panel C**), after treatment with 0.2 μM bicuculline methiodide (**panel D**), and after a second 0.2 μM bicuculline treatment (**panel E**). For this article, there are 110 different combinations of the two uniformity classifications, five leader/follower strength classifications, and 11 peak count classifications. Empty (white) symbols described 0% of the correlograms. Shaded symbols indicate that at least one correlogram in the culture was described by the given classification combination. Shading ranges from dark blue (>0%) to dark red (100%). Triangles pointing up represent uniform correlograms while triangles pointing down represent nonuniform correlograms. |

Supplementary Fig. S5 shows how correlogram classifications evolved for each treatment. In general, uniform correlograms stayed uniform ~80% of the time. However, after medium leak, uniform correlograms stayed uniform less than 60% of the time, possibly due to the reactivation of a subset of signals, as discussed for Supplementary Fig. S1. Nonuniform correlograms stayed nonuniform 80-90% of the time after most treatments. However, after impact, nonuniform correlograms stayed nonuniform only 70% of the time. Correlogram peak count transitions remained fairly stable between treatments. After impact, leader/follower classifications switched randomly for intermediate, fairly strong, and strong classification levels (transition probabilities were more uniform, resulting in flatter transition distributions). This observation suggests that stronger leader/follower pairs were not consistently stronger. Additionally, this random switching after impact may indicate sparser correlograms.

Together, these results demonstrate that damage due to impact injury influences firing in a culture. While bicuculline can standardize cell firing, changes arising from various treatments may be less apparent with such standardization. One likely explanation for this result is that injury affects GABAergic signaling (Parga Becerra et al., 2021), and decreases inhibitory signaling in general (Lee et al., 2019). With bicuculline already inhibiting GABAergic inhibitory signaling in a culture, a decrease in inhibitory signaling after impact would have less effect on overall culture firing, and cell interactions would therefore undergo less change. However, the overall changes in rhythmic activity after impact are consistent between the recording with and without bicuculline. This observation suggests that, with and without bicuculline, damage alters the rhythmic dynamics of excitatory signaling, likely by both removing inhibitory interactions and altering excitatory firing.

| 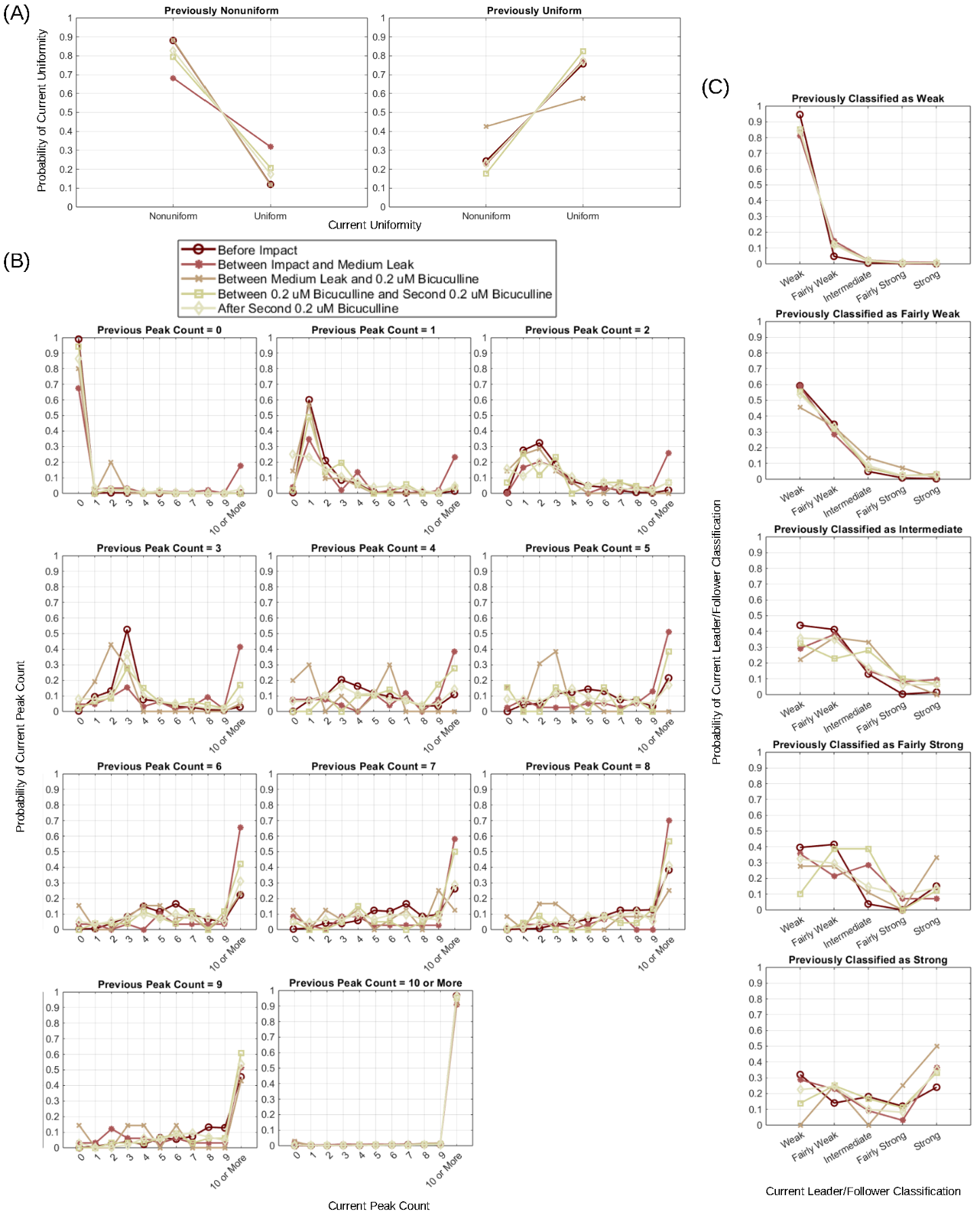 |
| --- |
| **Fig. S5.** Evolution of correlogram classifications before and after impact, medium leak, and two subeffective doses of bicuculline (0.2 μM). Correlogram uniformity (A), peak count (B), and leader/follower nature (C) were classified as described in main text Sect. “Correlogram Shape Quantification”. To determine whether certain classifications were more likely to change than others as a result of treatment, classifications were tracked between treatments. The probability of each correlogram classification in analysis region 2 through 66 was calculated based on the correlogram’s classification in the previous analysis region (1-65), then all regions were grouped by treatment. |

# **Section S2: Supplementary Figures**

| 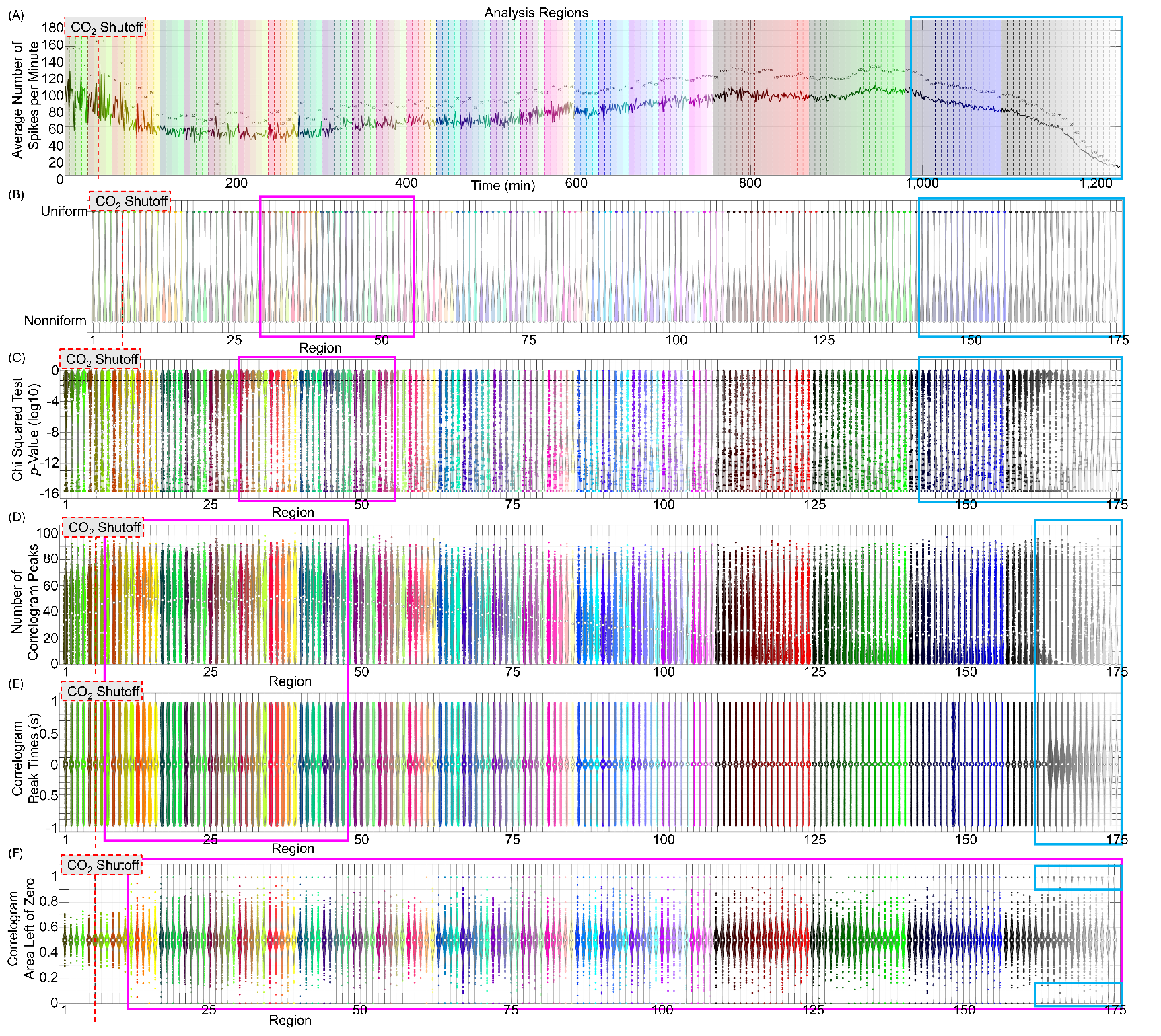 |
| --- |
| **Fig. S6. Correlogram metric distributions as a result of pH shock after CO_2_ shutoff** (red dashed lines). **Panel A** shows the analysis regions in which correlograms were generated for the entire recording overlayed over the mean spike count per minute. Violin plots are displayed to view population level changes in correlogram properties across analysis regions. Correlogram uniformity and probability of being uniform are shown in **panel B and C**, peak count and peak times in **panel D and E**, and area left of zero in **panel F**. Individual correlogram metrics for each analysis region are shown in Supplementary Movies S1-S3 and correspond to individual points in the violins. White points in the violins represent the median metric value. Magenta boxes indicate metric alterations in response to altered pH. Blue boxes indicate metric alterations due to cell death, as described in main text Sect. “Violin Plots to Visualize Correlogram Metric Distributions”. |

| 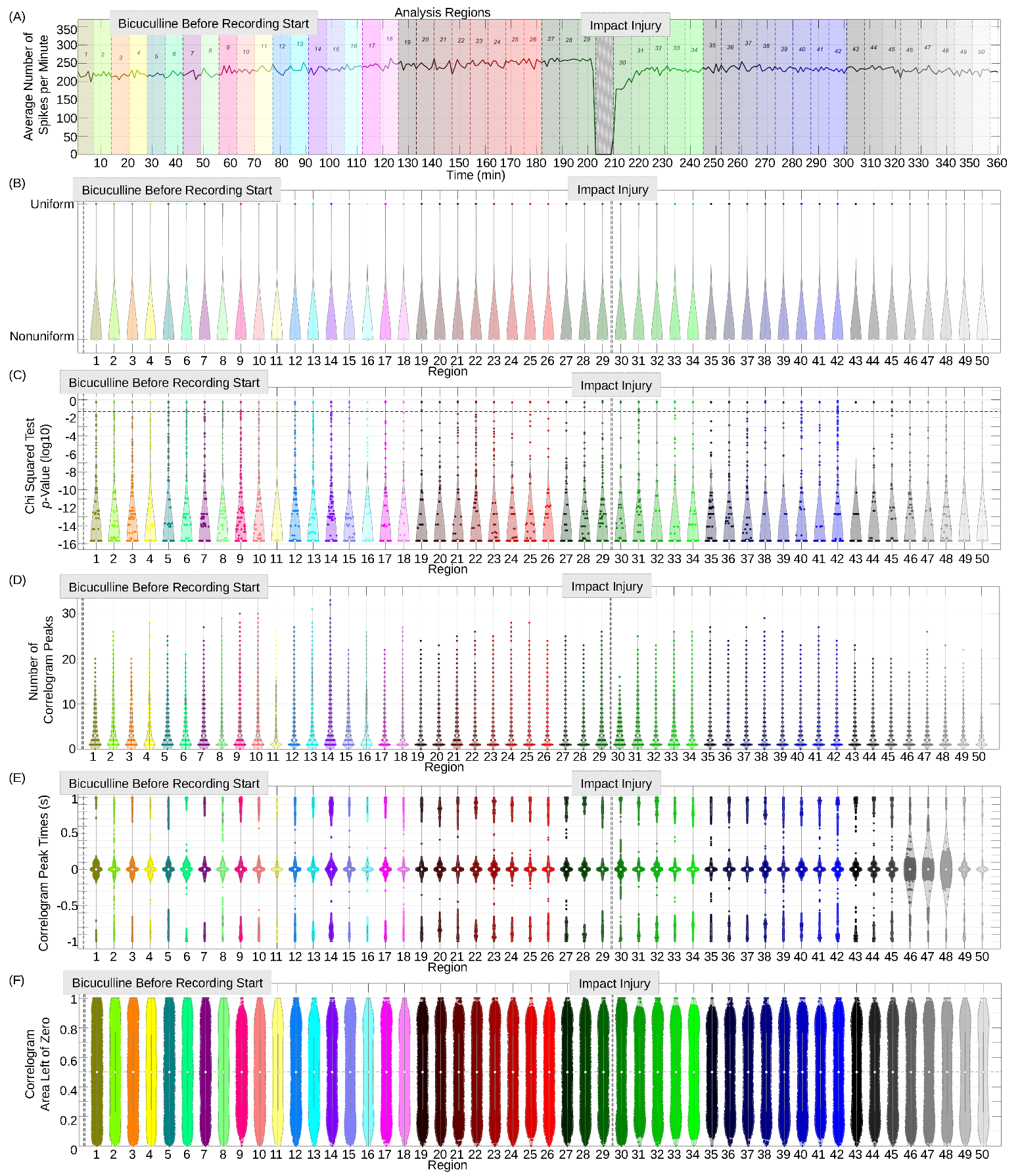 |  |
| --- | --- |
| **Fig. S7. Correlogram metric distributions as a result of impact under bicuculline.** **Panel A** shows the analysis regions in which correlograms were generated for the entire recording overlayed over the mean spike count per minute. Violin plots are displayed to view population level changes in correlogram properties across analysis regions. Correlogram uniformity and probability of being uniform are shown in **panel B and C**, peak count and peak times in **panel D and E**, and area left of zero in **panel F**. Individual correlogram metrics for each analysis region are shown in Supplementary Movies S4-S6 and correspond to individual points in the violins. White points in the violins represent the median metric value. |  |
| \| 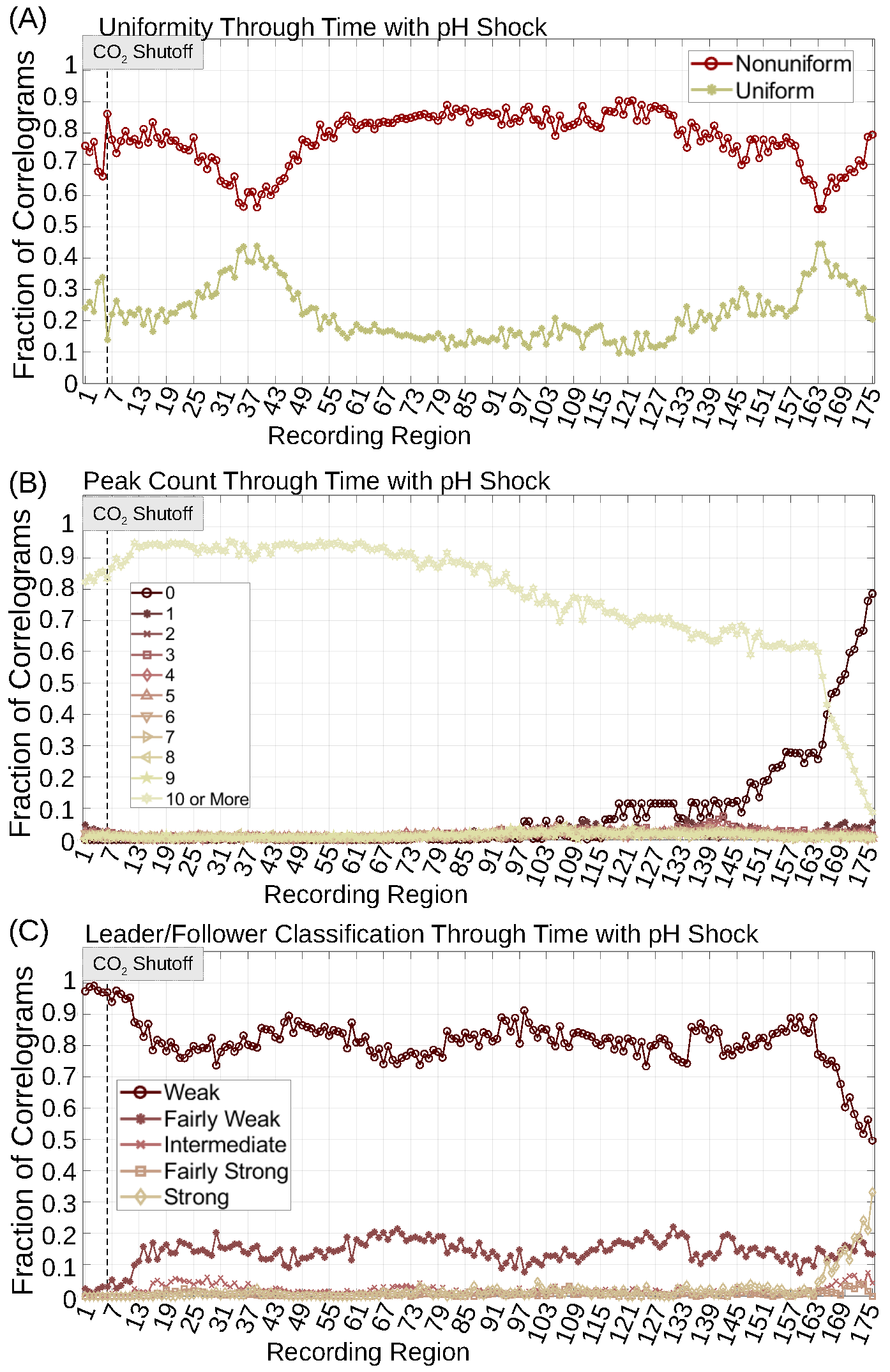 \| \| --- \| \| **Fig. S8. Correlogram classification summaries for pH shock.**  Correlogram uniformity (**panel A**), peak count (**panel B**), and leader/follower consistency (**panel C**) were classified as described in methods Sect. “Correlogram Shape Quantification”. The fraction of correlograms with each classification are shown for a microelectrode array recording in which the culture was subjected to alkalosis. Dashed vertical lines indicate when the culture’s CO_2_ supply was deactivated and alkalosis commenced. \|  \| \| 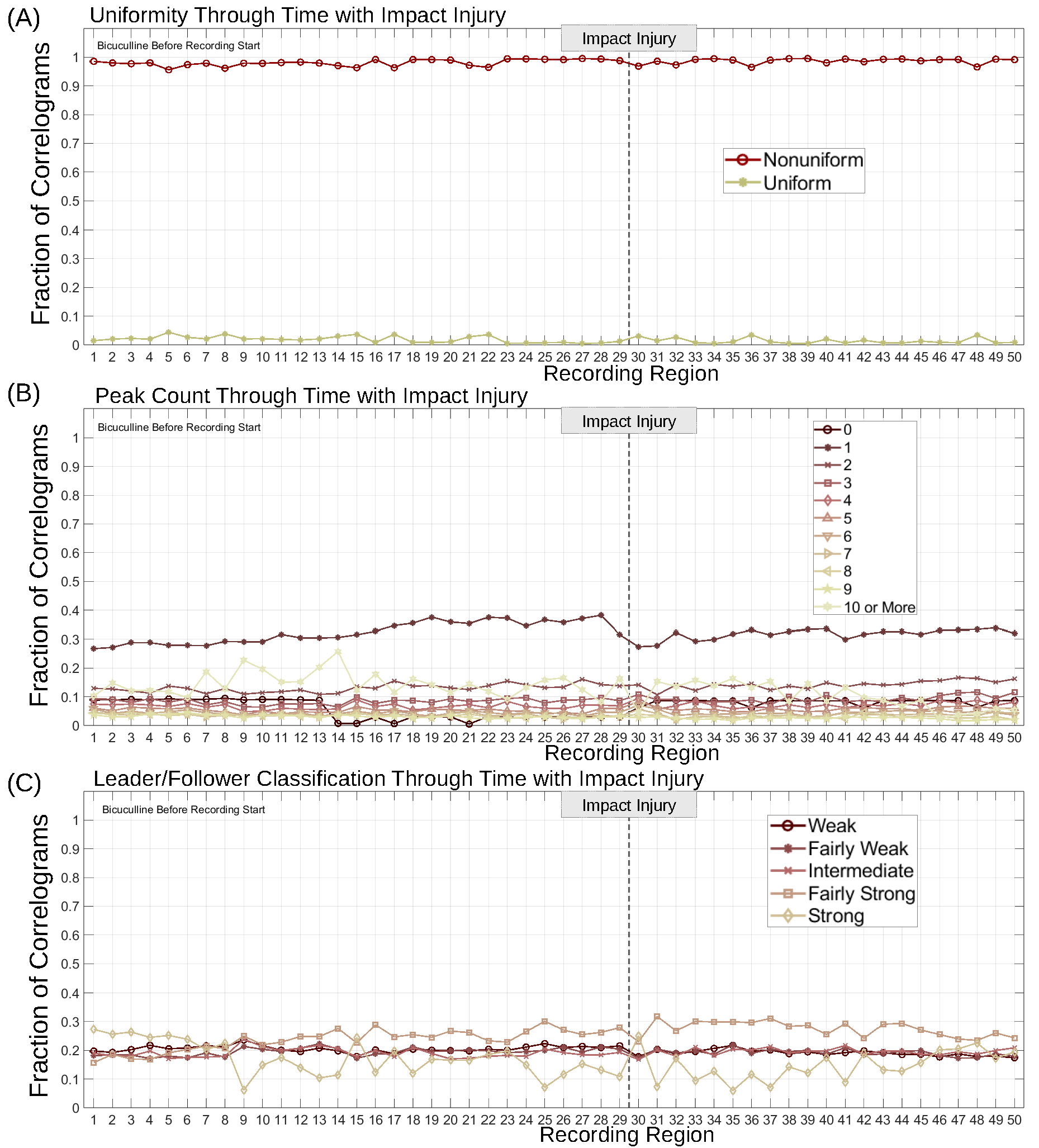 \| \| --- \| \| \| --- \| --- \| \| **Fig. S9. Correlogram classification summaries for impact injury under bicuculline.**  Correlogram uniformity (**panel A**), peak count (**panel B**), and leader/follower consistency (**panel C**) were classified as described in methods Sect. “Correlogram Shape Quantification”. The fraction of correlograms with each classification are shown for a microelectrode array recording in which the culture was subjected to impact injury. Dashed vertical lines indicate when the impact injury was administered. \|  \| 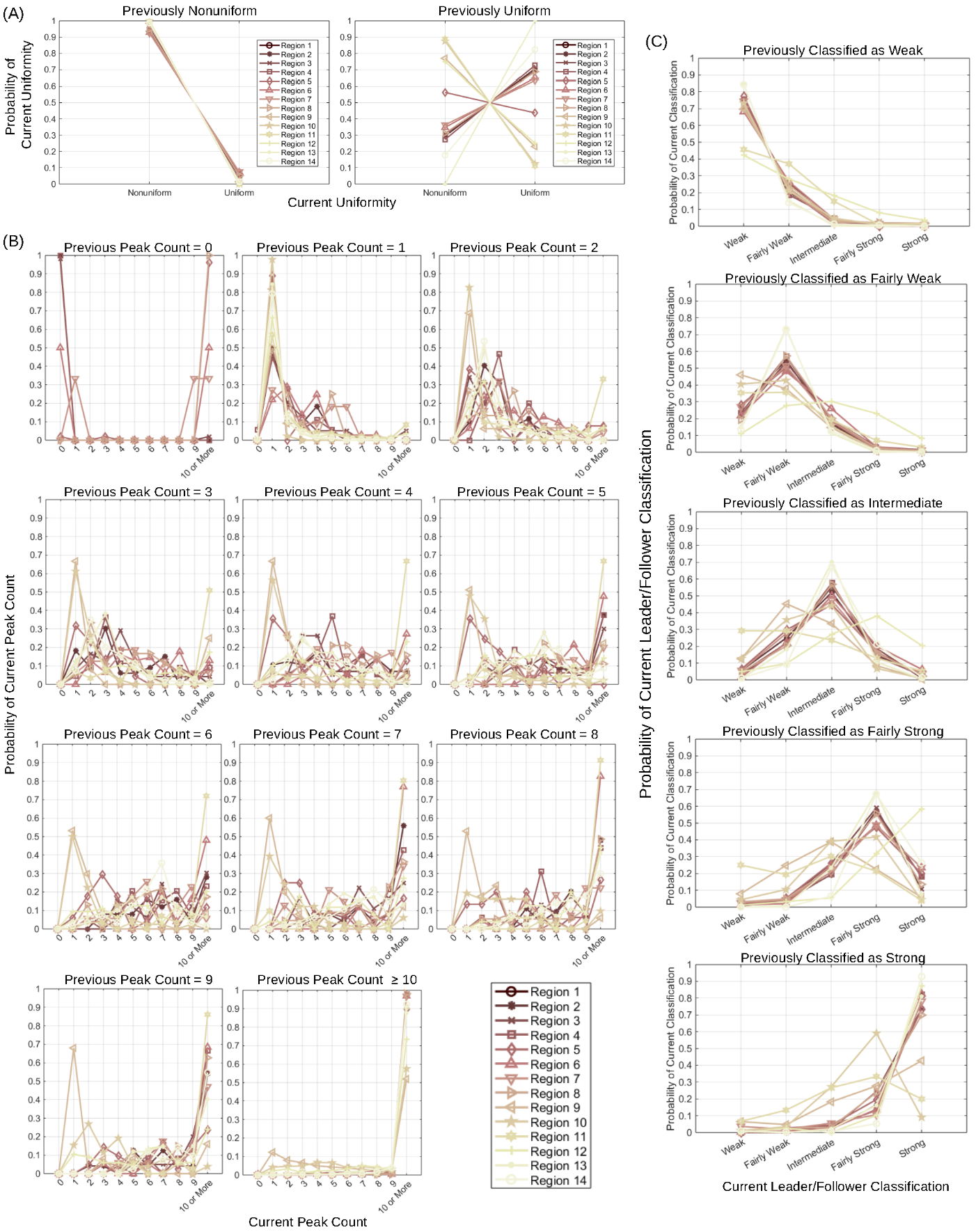 \| \| --- \| \| **Fig. S10. Evolution of correlogram classifications before and after 40 μM bicuculline methiodide treatment.** Correlogram uniformity (**panel A**), peak count (**panel B**), and leader/follower nature (**panel C**) were classified as described in methods Sect. “Correlogram Shape Quantification”. Correlograms of a specific classification may be more likely to change in response to bicuculline. To identify such correlograms, classifications were tracked between analysis regions (shown in main text Fig. 6A). For all but the first analysis region, correlograms were grouped based on the classification in the previous analysis region. For each group, a histogram of correlogram classification in the current analysis region was created. This histogram was then normalized to probability and therefore represents the probability of exhibiting each classification given the correlogram’s classification in the previous analysis region. \|   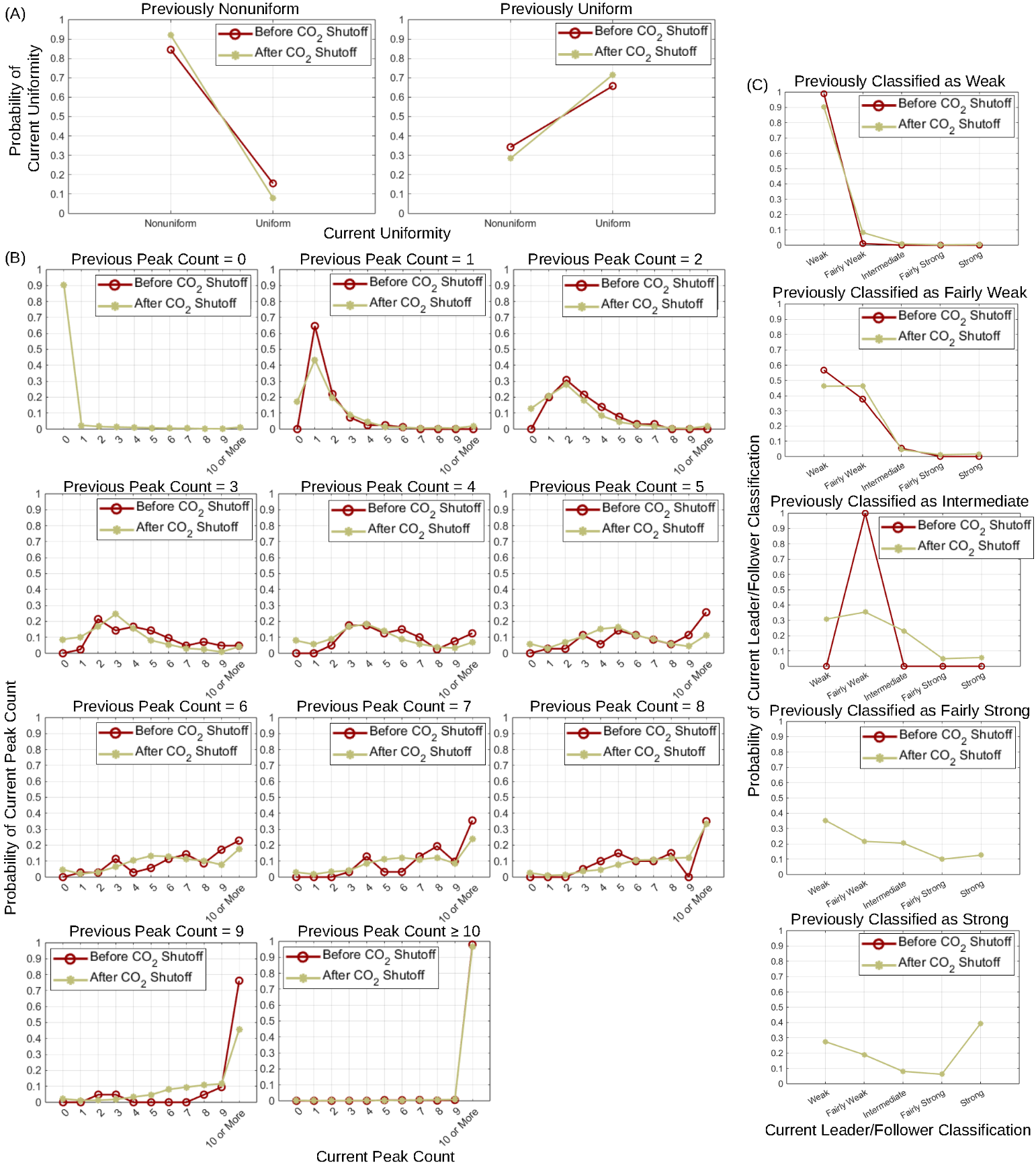 | |
| **Fig. S11. Evolution of correlogram classifications before and after pH shock.** Correlogram uniformity (**panel A**), peak count (**panel B**), and leader/follower nature (**panel C**) were classified as described in methods Sect. “Correlogram Shape Quantification”. Correlograms of a specific classification may be more likely to change in response to alkalosis. To identify such correlograms, classifications were tracked between analysis regions (shown in Supplementary Fig. S6A). For all but the first analysis region, correlograms were grouped based on the classification in the previous analysis region. For each group, a histogram of correlogram classification in the current analysis region was created. This histogram was then normalized to probability and therefore represents the probability of a correlogram exhibiting each classification given the correlogram’s classification in the previous analysis region. All regions were then grouped by before vs. after pH shock. If no distribution is plotted, no correlograms exhibited the given classification. | |
|  | |
| 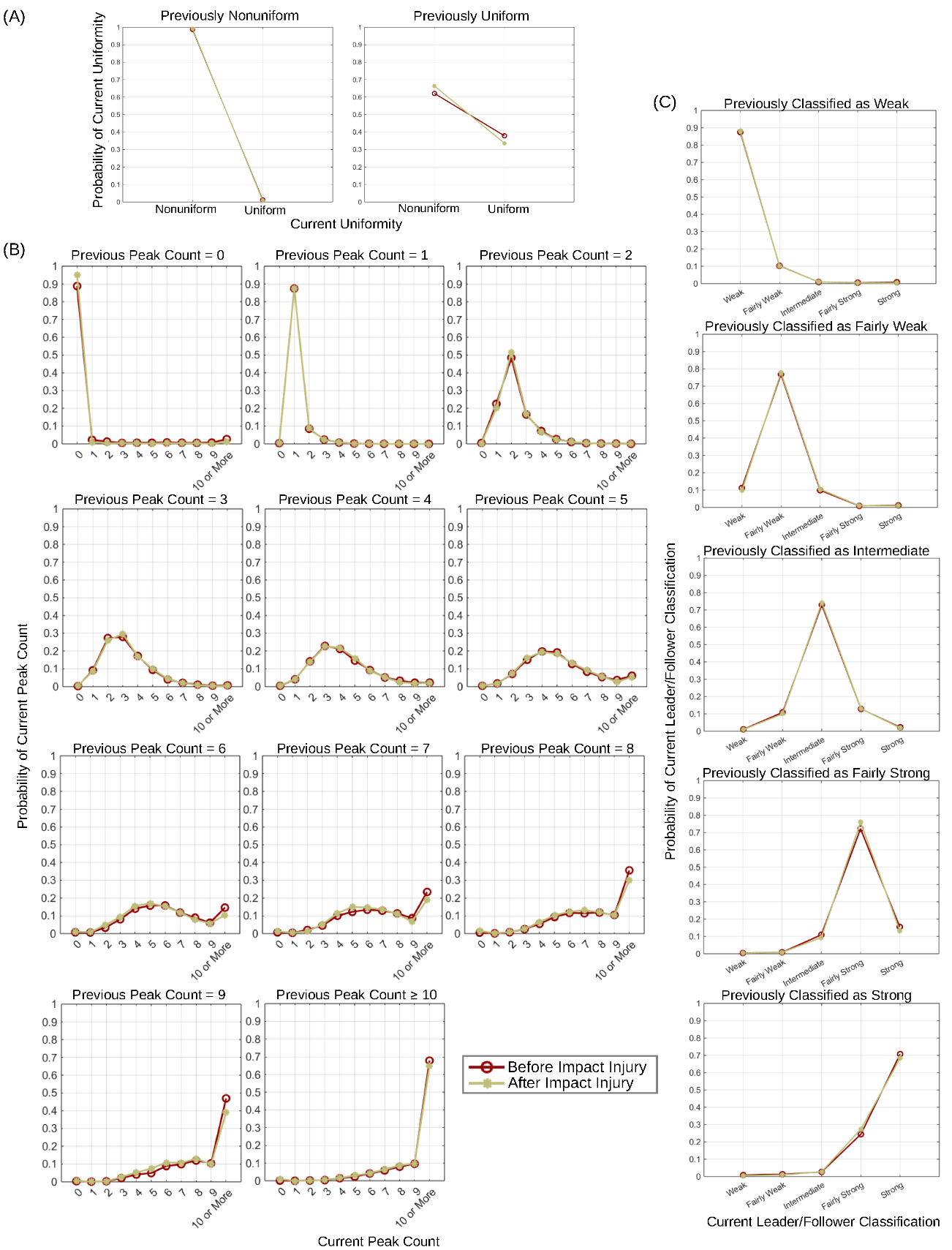 | |
| **Fig. S12. Evolution of correlogram classifications before and after impact under bicuculline.** Correlogram uniformity (**panel A**), peak count (**panel B**), and leader/follower nature (**panel C**) were classified as described in methods Sect. “Correlogram Shape Quantification”. Correlograms of a specific classification may be more likely to change in response to injury. To identify such correlograms, classifications were tracked between analysis regions (shown in Supplementary Fig. S7A). For all but the first analysis region, correlograms were grouped based on the classification in the previous analysis region. For each group, a histogram of correlogram classification in the current analysis region was created. This histogram was then normalized to probability and therefore represents the probability of a correlogram exhibiting each classification given the correlogram’s classification in the previous analysis region. All regions were then grouped by before vs. after injury. | |

**Section S3: Description of Supplementary Movies**

**Supplementary Movie S1** shows uniformity heatmaps for each analysis region of the pH shock recording. Light and dark green pixels indicate uniform and nonuniform correlograms, respectively. White pixels indicate correlograms involving cells that did not fire in the given analysis region.

**Supplementary Movie S2** shows peak count heatmaps for each analysis region of the pH shock recording. A brighter color indicates more peaks. White pixels indicate correlograms involving cells that did not fire in the given analysis region.

**Supplementary Movie S3** shows area left of zero heatmaps for each analysis region of the pH shock recording. For area left of zero, a value of 1 (red) indicates that the comparison cell (y-axis) always fired before the reference cell (x-axis). A value of 0 (blue) indicates that the comparison always fired after the reference. A value of 0.5 (green) indicates that there is no consistent firing order between the pair of cells. White pixels indicate correlograms involving cells that did not fire in the given analysis region.

**Supplementary Movie S4** shows uniformity heatmaps for each analysis region of the impact recording under bicuculline. Light and dark green pixels indicate uniform and nonuniform correlograms, respectively. White pixels indicate correlograms involving cells that did not fire in the given analysis region.

**Supplementary Movie S5** shows peak count heatmaps for each analysis region of the impact recording under bicuculline. A brighter color indicates more peaks. White pixels indicate correlograms involving cells that did not fire in the given analysis region.

**Supplementary Movie S6** shows area left of zero heatmaps for each analysis region of the impact recording under bicuculline. For area left of zero, a value of 1 (red) indicates that the comparison cell (y-axis) always fired before the reference cell (x-axis). A value of 0 (blue) indicates that the comparison always fired after the reference. A value of 0.5 (green) indicates that there is no consistent firing order between the pair of cells. White pixels indicate correlograms involving cells that did not fire in the given analysis region.

**Supplementary Movie S7** shows uniformity heatmaps for each analysis region of the impact recording without bicuculline. Light and dark green pixels indicate uniform and nonuniform correlograms, respectively. White pixels indicate correlograms involving cells that did not fire in the given analysis region.

**Supplementary Movie S8** shows peak count heatmaps for each analysis region of the impact recording without bicuculline. A brighter color indicates more peaks. White pixels indicate correlograms involving cells that did not fire in the given analysis region.

**Supplementary Movie S9** shows area left of zero heatmaps for each analysis region of the impact recording without bicuculline. For area left of zero, a value of 1 (red) indicates that the comparison cell (y-axis) always fired before the reference cell (x-axis). A value of 0 (blue) indicates that the comparison always fired after the reference. A value of 0.5 (green) indicates that there is no consistent firing order between the pair of cells. White pixels indicate correlograms involving cells that did not fire in the given analysis region.

# **Section S4: Literature Cited**

Atlan, L. S., & Margulies, S. S. (2019). Frequency-Dependent Changes in Resting State Electroencephalogram Functional Networks after Traumatic Brain Injury in Piglets. *Journal of Neurotrauma*, *36*(17), 2558–2578. https://doi.org/10.1089/neu.2017.5574

Gramowski, A., Stuewe, Simone, S., Jügelt, K., Schiffmann, D., Loock, J., Schroeder, O., Gross, G. W., & Weiss, D. G. (2006). *Detecting neurotoxicity through electrical activity changes of neuronal networks on multielectrode neurochips*. 410–415.

Gross, G. (1995). The use of neuronal networks on multielectrode arrays as biosensors. *Biosensors and Bioelectronics*, *10*(6–7), 553–567. https://doi.org/10.1016/0956-5663(95)96931-N

Gross, G. W., Harsch, A., Rhoades, B. K., & Göpel, W. (1997). Odor, Drug and Toxin Analysis with Neuronal Networks in Vitro: Extracellular Array Recording of Network Responses. *Biosensors and Bioelectronics*, *12*(5), 373–393. https://doi.org/10.1016/S0956-5663(97)00012-2

Ianof, J. N., & Anghinah, R. (2017). Traumatic brain injury: An EEG point of view. *Dementia & Neuropsychologia*, *11*(1), 3–5. https://doi.org/10.1590/1980-57642016dn11-010002

Lee, K. Y., Ratté, S., & Prescott, S. A. (2019). Excitatory neurons are more disinhibited than inhibitory neurons by chloride dysregulation in the spinal dorsal horn. *ELife*, *8*, e49753. https://doi.org/10.7554/eLife.49753

Parga Becerra, A., Logsdon, A. F., Banks, W. A., & Ransom, C. B. (2021). Traumatic Brain Injury Broadly Affects GABAergic Signaling in Dentate Gyrus Granule Cells. *Eneuro*, *8*(3), ENEURO.0055-20.2021. https://doi.org/10.1523/ENEURO.0055-20.2021

Rogers, E. A., Beauclair, T., Martinez, J., Mufti, S. J., Kim, D., Sun, S., Stingel, R. L., Dieterly, A. M., Krishnan, N., Crodian, J., & Shi, R. (2023). The contribution of initial concussive forces and resulting acrolein surge to β-amyloid accumulation and functional alterations in neuronal networks using a TBI-on-a-chip model. *Lab on a Chip*, *23*(15), 3388–3404. https://doi.org/10.1039/D3LC00248A

Rogers, E. A., Beauclair, T., Thyen, A., & Shi, R. (2022). Utilizing novel TBI-on-a-chip device to link physical impacts to neurodegeneration and decipher primary and secondary injury mechanisms. *Scientific Reports*, *12*(1), 11838. https://doi.org/10.1038/s41598-022-14937-w

Rogers, E. A., & Gross, G. W. (2019). Simultaneous electrophysiological and morphological assessment of functional damage to neural networks in vitro after 30–300 g impacts. *Scientific Reports*, *9*(1), 14994. https://doi.org/10.1038/s41598-019-51541-x
